# Physicochemical Properties and Route of Systemic Delivery Control the In Vivo Dynamics and Breakdown of Radiolabeled Gold Nanostars

**DOI:** 10.1002/smll.202204293

**Published:** 2023-03-25

**Authors:** Xiaona Wen, Luping Ou, Gabriel Cutshaw, Saji Uthaman, Yu-Chuan Ou, Tian Zhu, Sarah Szakas, Brandon Carney, Jacob Houghton, Alexander Gundlach-Graham, Marjan Rafat, Kai Yang, Rizia Bardhan

**Affiliations:** Department of Chemical and Biomolecular Engineering, Vanderbilt University, Nashville, TN 37235, USA; Nanovaccine Institute Iowa State University Ames, IA 50012, USA; Center for Soft Condensed Matter Physics and Interdisciplinary Research and School of Physical Science and Technology, Soochow University, Suzhou 215006, China; Nanovaccine Institute Iowa State University Ames, IA 50012, USA; Department of Chemical and Biological Engineering, Iowa State University Ames, IA 50012, USA; Nanovaccine Institute Iowa State University Ames, IA 50012, USA; Department of Chemical and Biological Engineering, Iowa State University Ames, IA 50012, USA; Department of Chemical and Biomolecular Engineering, Vanderbilt University, Nashville, TN 37235, USA; Department of Chemical and Biomolecular Engineering, Vanderbilt University, Nashville, TN 37235, USA; Department of Chemistry, Iowa State University Ames, IA 50011, USA; Department of Radiology Stony Brook University, Stony Brook, New York, NY 11794, USA; Department of Radiology Stony Brook University, Stony Brook, New York, NY 11794, USA; Department of Chemistry, Iowa State University Ames, IA 50011, USA; Department of Chemical and Biomolecular Engineering, Vanderbilt University, Nashville, TN 37235, USA; Center for Soft Condensed Matter Physics and Interdisciplinary Research and School of Physical Science and Technology, Soochow University, Suzhou 215006, China; Department of Chemical and Biological Engineering, Iowa State University Ames, IA 50012, USA; Nanovaccine Institute Iowa State University Ames, IA 50012, USA

**Keywords:** exocytosis, gold nanostars, in vivo breakdown, long-term biodistribution, Martini coarse-grained simulations, positron emission tomography (PET), protein corona

## Abstract

The in vivo dynamics of nanoparticles requires a mechanistic understanding of multiple factors. Here, for the first time, the surprising breakdown of functionalized gold nanostars (F-AuNSs) conjugated with antibodies and ^64^Cu radiolabels in vivo and in artificial lysosomal fluid ex vivo, is shown. The short-term biodistribution of F-AuNSs is driven by the route of systemic delivery (intravenous vs intraperitoneal) and long-term fate is controlled by the tissue type in vivo. In vitro studies including endocytosis pathways, intracellular trafficking, and opsonization, are combined with in vivo studies integrating a milieu of spectroscopy and microcopy techniques that show F-AuNSs dynamics is driven by their physicochemical properties and route of delivery. F-AuNSs break down into sub-20 nm broken nanoparticles as early as 7 days postinjection. Martini coarse-grained simulations are performed to support the in vivo findings. Simulations suggest that shape, size, and charge of the broken nanoparticles, and composition of the lipid membrane depicting various tissues govern the interaction of the nanoparticles with the membrane, and the rate of translocation across the membrane to ultimately enable tissue clearance. The fundamental study addresses critical gaps in the knowledge regarding the fate of nanoparticles in vivo that remain a bottleneck in their clinical translation.

## 1. Introduction

Engineered gold nanoparticles (NPs) have driven a paradigm shift in nanomedicine attributable to their straightforward synthesis, ease of surface functionalization, and high biocompatibility in vivo,^[1,2]^ and some of gold NPs (AuNPs) are being used in clinical pilot studies in humans.^[3]^ Gold nanostars (AuNSs), in particular, have propelled advances in imaging and treatment due to their anisotropic architecture which enables their utility as contrast agents with significant signal enhancement,^[4–6]^ and as photothermal agents with enhanced light to heat conversion.^[7–9]^ Yet, despite these innovations AuNSs have failed to transition to the clinic due to gaps in our understanding of their short- and long-term fate in vivo. Many critical parameters that are essential to AuNSs clinical translation remain underexplored thus far. These parameters include 1) AuNSs disintegration in vivo and the role of shape and surface properties in the breakdown process, 2) the impact of route of delivery on AuNSs in vivo accumulation and breakdown, 3) uptake by organs of the mononuclear phagocyte system (MPS), and long-term toxicity of AuNSs, and 4) transcellular transport of AuNSs and in vivo clearance. An in-depth understanding of these foundational parameters will ultimately enable the design of clinically translatable AuNP probes that are not only excellent diagnostic and therapeutic agents but also have rapid clearance to minimize long-term adverse effects. This in vivo dynamics is correlated to the adsorption of serum proteins (opsonization) from the biological milieu on the NP surface to form a protein corona. The opsonization process is governed by the physicochemical properties of NPs that include the shape, size, charge, and surface ligands, and control both the quantity and composition of proteins adsorbed.^[10–12]^ Opsonization of NPs subsequently controls the uptake, endocytosis pathway, intracellular trafficking, and exocytosis of NPs from tissue. Whereas the utility of polymeric coatings such as polyethylene glycol (PEG) has been implemented to reduce protein corona formation, PEG-coated NPs have only shown modest improvement in minimizing opsonization.^[13]^ Therefore, we contend that understanding the aforementioned fundamental parameters is key to addressing the deficiencies in NP-based diagnostics and therapeutics, and facilitates successful preclinical development and clinical translation of AuNSs.

With this goal, our study aimed to show the global impact of shape, surface properties, opsonization, and the route of systemic delivery including intravenous (IV) and intraperitoneal (IP) of NPs functionalized with various surface moieties on their long-term (1 to 90 days) in vivo fate. PEG-coated NPs have been extensively studied in the literature^[14,15]^ enabling passive accumulation at the diseased site, and many PEG-coated NPs are already in clinical use (e.g., Doxil). However, many in vivo studies in recent years have used AuNPs with multiple payloads for imaging and therapy, and have conjugated ligands, such as antibodies, on their surface for targeted delivery at the diseased site.^[16–18]^ To guide this expansive community of researchers, we aimed to ensure that our study can serve as a blueprint for the systematic and longitudinal investigation of the biodistribution of AuNPs, and to understand what experiments would be feasible and what may not be recommended. The goal of our fundamental work was not to focus on a specific disease model (e.g., tumor models or colitis models would not be possible for 90 days) or even a specific disease marker. To mimic the characteristics of how an antibody functionalized NP would behave in vivo we conjugated nonspecific IgG antibodies on the AuNSs. Whereas the IgG antibodies will not target specific receptors, its presence on the AuNS surface will control the fluid dynamics of the NP in the blood stream including laminar flow and Brownian motion, similar to any other targeting antibodies. In addition, antibodies not only control the “molecular characteristics” of NPs (i.e., actively target a protein) but also govern the “mechanical characteristics” of NPs (i.e., the overall stiffness). These properties direct cellular uptake and are important in the overall biodistribution of NPs, and are distinct for antibody-coated versus PEG-coated NPs. Therefore, the intent of our study was to represent a generalizable NP that has all of the components (i.e., targeting ligands, diagnostic, and therapeutic functionality) being explored by many researchers in the field of nanomedicine. Our overarching goal here is to capture many of the aforementioned phenomena that NPs encounter in vivo. Therefore, we evaluated the in vivo dynamics of functionalized gold nanostars (F-AuNSs) conjugated with IgG antibodies, and chelated with ^64^Cu radiolabels. This study also built upon our previous work where we demonstrated AuNSs functionalized with antibodies, Raman tags, and radiolabels circulated in vivo and tracked multiple immunomarkers in response to immunotherapy.^[4]^

In this work, we employed positron emission tomography and computed tomography (PET/CT) imaging and gamma counts of F-AuNSs, and in conjunction with transmission electron micrograph (TEM) and inductively coupled plasma mass spectrometry (ICP-MS). We showed that the route of systemic administration had a significant impact on F-AuNSs accumulation. Our results indicated that F-AuNSs had minimum toxicity and did not invoke any immune response in vivo even after 90 days. We also showed for the first time, the surprising breakdown of F-AuNSs in vivo as early as 7 days postdelivery. By integrating Martini coarse-grain (CG) simulations we highlighted that after breakdown of F-AuNSs, the translocation of the resulting broken NPs was controlled by their size, shape, surface charge, and unexpectedly also the composition of the lipid bilayer membranes representing the different tissues where F-AuNSs were accumulated (i.e., liver, spleen, etc.). To understand how the physicochemical properties of F-AuNSs drove their in vivo behavior, we examined their cellular-level endocytosis and intracellular trafficking, and interaction with serum proteins. We concluded that functionalization of AuNSs and the resulting surface properties not only minimized opsonization but also altered the composition of the absorbed proteins which promoted longer circulation and uptake in vivo. This comprehensive mechanistic study revealed that physicochemical properties, protein corona formation, and route of systemic delivery are not independent parameters but simultaneously play a role in long-term in vivo fate of NPs and ultimate clinical translation.

## 2. Results and Discussion

**Figure 1.**
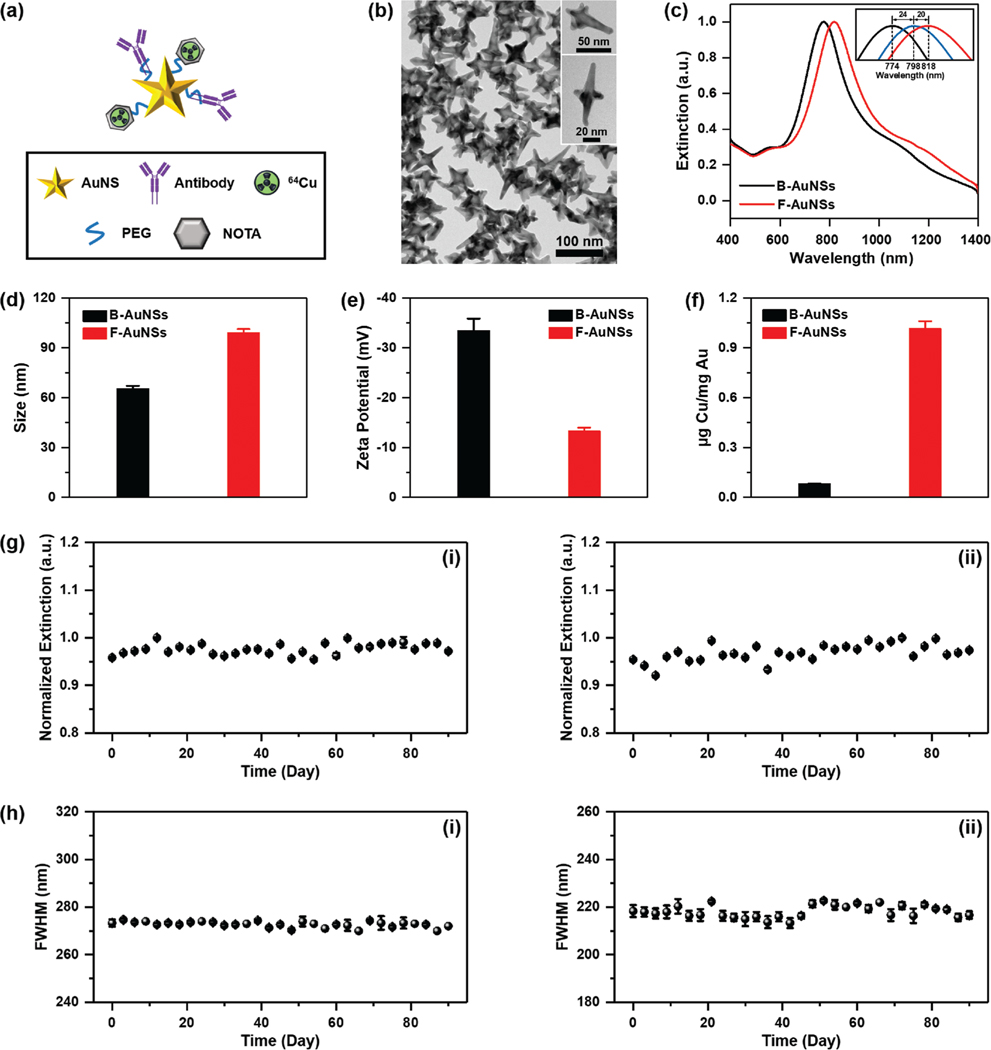
Design and characterization of B-AuNSs and F-AuNSs. a) Schematic illustration showing F-AuNSs were conjugated with IgG2a antibodies and chelator NOTA via a bifunctional linker followed by chelation with ^64^Cu radioisotopes. b) Transmission electron image of B-AuNSs showing their anisotropic structure. c) Extinction spectra of B-AuNSs and F-AuNSs. The inset showed peak shifts when B-AuNSs (black) were bound with antibodies (blue) and further ^64^Cu-NOTA complex to form F-AuNSs (red). d) Hydrodynamic size and e) zeta potential of B-AuNSs and F-AuNSs from dynamic light scattering. f) Quantification of cold Cu chelation to F-AuNSs with ICP-MS. g) Normalized extinction and h) FWHM of concentrated F-AuNSs that were dispersed in (i) water and (ii) media and measured every three days over 90 days. F-AuNSs were stored at 4 °C between measurements. All data were represented as mean ± standard deviation (*n* = 3).

Bare gold nanostars (B-AuNSs) were synthesized through a one-step seedless method with a biological buffer, 4-(2-hydroxyethyl)-1-piperazine-ethanesulfonic acid (HEPES) as described in our previously published work.^[4,6,19]^ The low binding affinity of HEPES on gold surface facilitated straightforward surface modification enabling us to design the F-AuNSs. F-AuNSs were synthesized by conjugating antibodies and chelators to AuNS surfaces via a bifunctional orthopyridyl disulphide-poly(ethylene glycol)-succinimidyl valerate (OPSS-PEG-SVA) linker. The thiols on the OPSS group were covalently bound to AuNSs and SVA esters reacted with amines on antibodies or chelators to form a stable amide bond (Figure 1a). We chose a generic IgG antibody to explore untargeted biodistribution of F-AuNSs, as antibody-nanoparticle conjugates have prolonged blood circulation, enhanced nanoparticle-cellular interactions, and longer residence time in the tissues.^[20]^ The PEG ligands on the linker hindered protein adsorption and subsequent clearance by the MPS.^[21]^ The diagnostic capability of F-AuNSs to provide anatomical information in vivo was enabled by the utility of ^64^Cu radiolabel chelated to 2-S-(4-aminobenzyl)-1,4,7-triazacyclononane-1,4,7-triacetic acid) (p-NH_2_-Bn-NOTA) and conjugated to AuNSs via the same linker. We chose ^64^Cu-NOTA complex for F-AuNSs enabled PET/CT imaging due to its high labeling yield,^[22–24]^ and clinical significance (clinical trial #NCT04167969).^[25]^ B-AuNSs were overall 60–80 nm (Figure 1b), a size regime enabling longitudinal imaging.^[21]^ The functionalization of AuNSs with antibodies and ^64^Cu-NOTA resulted in a ≈40 nm shift in the plasmon resonance (Figure 1c) indicating an increase in particle size and change in refractive index of the media. Consistent with UV–vis results, the size of B-AuNSs and F-AuNSs measured using dynamic light scattering showed similar trends (Figure 1d). F-AuNSs also showed a near-neutral surface charge (Figure 1e) attributable to the PEG chains in the linker, which is desirable to minimize opsonization and promote longer circulation half-life. We further confirmed Cu chelation to NOTA conjugated AuNSs with ICP-MS using “cold” nonradioactive Cu. ICP-MS showed 1.02 ± 0.044 μg Cu/mg Au for F-AuNSs relative to 0.084 ± 0.0010 μg Cu/mg Au for B-AuNSs control indicating successful radiolabeling (Figure 1f). The F-AuNSs demonstrated high stability in various media including water, phosphate buffered saline (PBS), and cell culture media (supplemented with and without serum) for up to 90 days (Figure 1g,h; Figure S1, Supporting Information). Minimal changes were observed in the intensity and full width at half maximum (FWHM) of the extinction spectra during this time span indicating long shelf-life of F-AuNSs.

**Figure 2.**
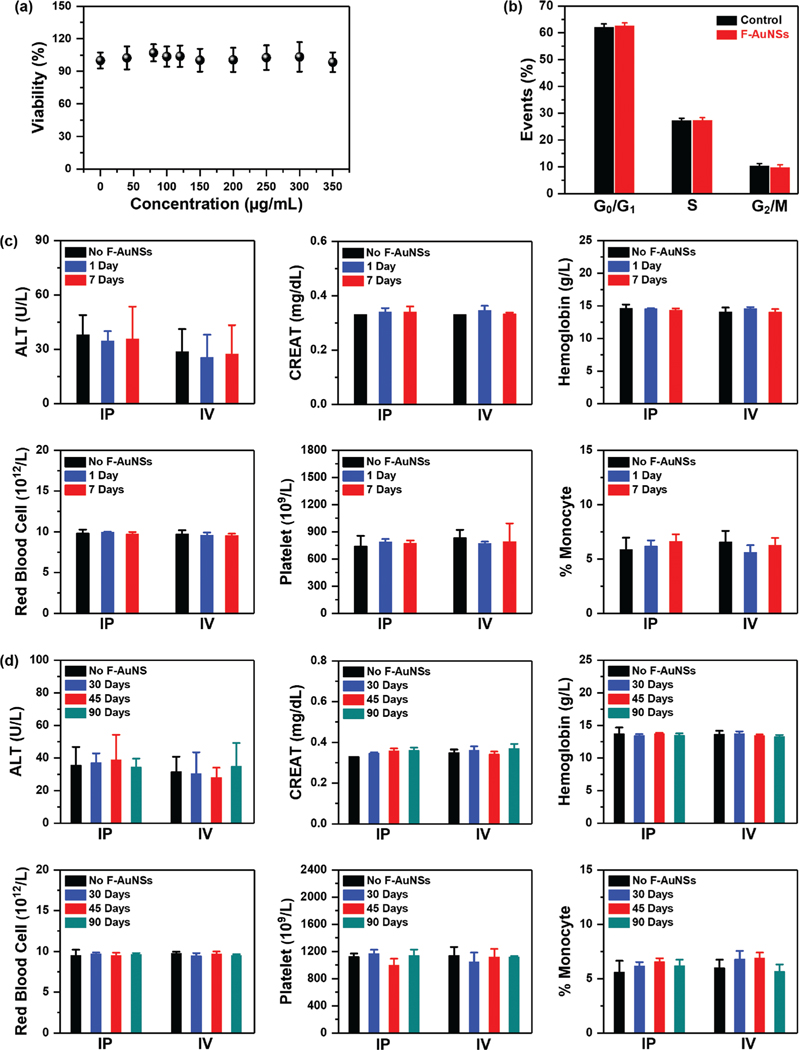
Toxicity evaluation of F-AuNSs in vitro and in vivo. a) MTT cell viability assay of J774A.1 cells incubated for 24 h with F-AuNSs at 0–350 μg mL^−1^ concentrations (*n* = 5 per concentration and *N* = 3 independent experiments). Cell viability was measured at 540 nm. All data were represented as mean ± standard deviation. b) Cell cycle analysis of J774A.1 cells upon incubation with F-AuNSs (100 μg mL^−1^) for 24 h. No significant changes were observed in the different cell cycle phases compared to control cells that did not receive F-AuNSs in both macrophage cell lines. All data were represented as mean ± standard deviation (*n* = 6 per group and *N* = 3 independent experiments). Serum inflammatory markers and complete blood count of mice received F-AuNSs IP and IV were compared to control mice which received PBS at both short- and long-terms. c) Short-term measurements included 1- and 7-days postdelivery results. d) Long-term was defined as 30-, 45-, and 90-days postinjection results. Irrespective of the route of delivery, no significant abnormalities based on the unpaired two-sided Student’s *t*-tests in hepatic, renal, and hematological functions were observed, indicating high biocompatibility of F-AuNSs. All data were represented as mean ± standard deviation (*n* = 4).

Next, we studied the biocompatibility of AuNSs both in vitro and in vivo. We first studied viability of F-AuNSs in vitro in murine macrophage cell lines J774A.1 and RAW 264.7 and observed minimal changes in the viability of both cell lines indicating F-AuNSs had high in vitro biocompatibility (Figure 2a; Figure S2a, Supporting Information). Macrophage cells are accurate representation of the in vivo microenvironment since NPs are endocytosed by spleen- and liver-resident macrophages.^[26–28]^ We also studied the impact of F-AuNSs on cell cycle since studies have shown NPs that are deemed nontoxic from classical toxicity assays often result in severe cell cycle disruption and DNA damage.^[29]^ Cell cycle includes four phases (G_1_, S, G_2_, and M) of cell division and replication where the activation of each phase is governed by the progression and completion of the previous phase.^[30,31]^ A cell cycle starts with the G_1_ phase where cells increase their size, followed by DNA synthesis during the S phase, and protein synthesis needed for cell division in the G_2_ phase. In the final M phase, cells divide forming two daughter cells. We evaluated the effect of F-AuNSs on cell cycle via flow cytometry, which showed minimal alterations in cell cycle stages in both cell lines (Figure 2b; Figure S2b, Supporting Information). Literature evidence suggests that the grafting density of polymers and other moieties on NP surface are directly correlated to alterations in cell cycle.^[32]^ Our results implied that optimal grafting density of functional groups was achieved in F-AuNS synthesis.

We then examined the biocompatibility of F-AuNSs in vivo and performed systematic evaluation of toxicology in healthy C57BL/6 mice by measuring standard serum inflammatory markers. Our goal was to correlate the toxicity profile to route of delivery as well as breakdown and transcytosis from tissues. Mice received F-AuNSs at a dose of 0.04 mg g^−1^ mouse weight IP or IV, and sera were collected from a parallel cohort of mice at 1-, 7-, 30-, 45-, and 90-days postdelivery to evaluate both short- and long-term response. This dosage of F-AuNSs is comparable to other studies utilizing gold nanostars.^[4,33]^ Alanine aminotransferase (ALT), aspartate aminotransferase (AST), and total bilirubin (TBIL) are measures of hepatic toxicity; high levels of these proteins are indicative of liver damage.^[34,35]^ Creatinine (CREAT) and blood urea nitrogen (BUN) are markers of kidney function. CREAT results from muscle metabolism and its concentration in sera correlates to NP glomerular filtration rate.^[36]^ BUN is derived from proteins and amino acid catabolism and filtered out via glomeruli. We also measured complete blood count in mouse sera, including hemoglobin, red blood cells, white blood cells, platelet concentration, monocyte counts, and lymphocyte counts. Our results showed mice that received F-AuNSs had comparable serum marker levels to control mice that received PBS both in the short- (1- and 7-days) and long-term (30-, 45-, and 90-days) indicative of minimal toxicity induced by F-AuNSs (Figure 2c,d; Figure S3, Supporting Information). The route of systemic delivery also did not induce any significant differences in these serum markers. We further confirmed these observations with hematoxylin and eosin (H&E) staining of major organs (Figures S4 and S5, Supporting Information) and observed no noticeable histopathological changes irrespective of route of administration.

**Figure 3.**
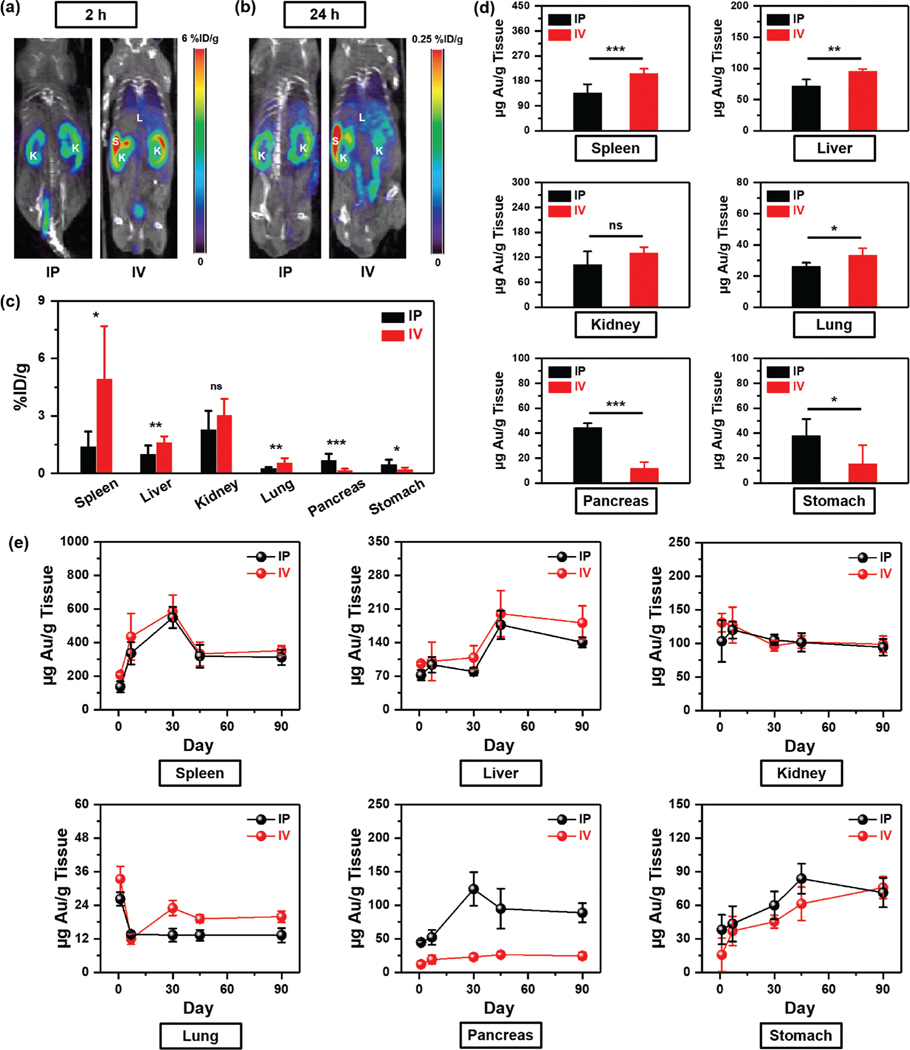
Biodistribution of F-AuNSs in vivo. Whole-body PET/CT images of mouse at a) 2 and b) 24 h post F-AuNSs delivery via IP or IV injection. *K* represents kidneys, *S* represents spleen, and *L* represents liver. c) Biodistribution of F-AuNSs from harvested organs via gamma counter after IP or IV injection at 24 h postinjection (*n* = 5). d) Quantitative ICP-MS analysis of biodistribution of F-AuNSs in mice 24 h post F-AuNSs delivery (*n* = 4). e) Biodistribution and clearance of F-AuNSs of major organs at 1-, 7-, 30-, 45-, and 90-days postdelivery (*n* = 4). Here, all data were represented as mean ± standard deviation. * indicates *p* < 0.05, ** indicates *p* < 0.01, *** indicates *p* < 0.001, and ns indicates not significant.

While few studies have demonstrated that the route of administration in vivo controls NP biodistribution,^[37,38]^ these studies are lacking longitudinal analysis elucidating the short- and long-term impact of route of administration on NP accumulation in various tissues. Here, we showed a correlative study combining PET/CT imaging, biodistribution via gamma counts of ^64^Cu radiolabeled F-AuNSs, and ICP-MS analysis of gold in tissues to assess the fate of F-AuNSs up to 90 days postinjection. PET/CT provides depth-resolved whole-body images of the localization of radiolabeled F-AuNSs in vivo in the first 24 h postinjection as a function of route of administration. Here, healthy C57BL/6 mice were injected 0.04 mg g^−1^ F-AuNSs via either IP or IV with ≈800 μCi of ^64^Cu radioactivity. PET images acquired at 2 and 24 h postinjection showed that F-AuNSs when delivered via IP were retained within the peritoneal cavity at 2 h time-point, and showed a preferential accumulation in the kidneys (Figure 3a,b). By contrast, mice that received F-AuNSs via IV had immediate localization in spleen, kidneys, and liver at 2 h time-point and further accumulation in these tissues over 24 h. Gamma count of harvested tissues at 24 h postinjection quantified a significantly higher uptake of F-AuNSs within spleen, liver, and lungs when delivered via IV (Figure 3c). IV delivery of F-AuNSs also showed higher uptake in heart, brain, bone, and muscle (Figure S6, Supporting Information) demonstrating that IV delivery resulted in NP accumulation in most organs. F-AuNSs delivered via IP had higher uptake in the pancreas and stomach likely due to the proximity of these organs to peritoneal cavity. Interestingly, route of administration had minimal difference in F-AuNSs accumulation in the kidneys and intestines (Figure 3c; Figure S6, Supporting Information). We further confirmed that the activity detected from gamma counts accurately represented ^64^Cu bound to F-AuNSs (not free ^64^Cu) by comparing with quantitative ICP-MS analysis of gold content in these organs retrieved 24 h post IP and IV delivery of F-AuNSs (Figure 3d; Figure S7a, Supporting Information). The results were consistent with findings from gamma count suggesting that ^64^Cu was appropriately chelated to F-AuNSs, and supporting our hypothesis that the route of administration had significant impact on tissue accumulation within the first 24 h postinjection. The ICP-MS analysis of gold content in blood showed a higher circulation of F-AuNSs at 24 h postinjection when delivered via IP (Figure S8a, Supporting Information). The results demonstrated that F-AuNSs had slower transport from peritoneal cavity to bloodstream when injected through IP delivery. In the case of delivery through IV injection, by 24 h time-point the F-AuNSs were likely already cleared from the blood and distributed to other organs. The route of delivery had no difference in F-AuNSs accumulation in the urine and feces at 24 h postinjection (Figure S8a, Supporting Information).

**Figure 4.**
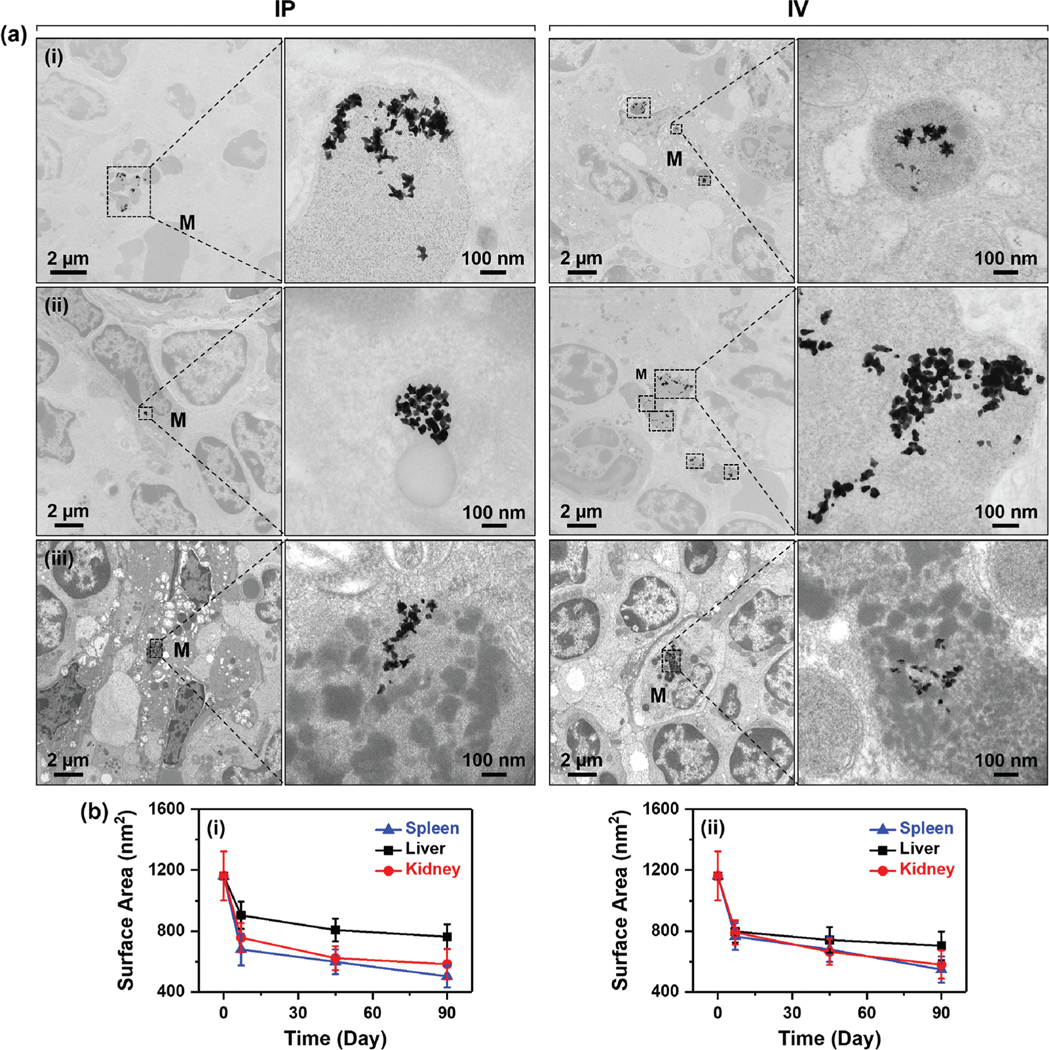
a) Representative TEM images of spleen of mice taken (i) 7-, (ii) 45-, and (iii) 90-days after IP injection shown in left, and IV injection shown in right of F-AuNSs. Here, *M* represents macrophage. b) Quantification of surface area of F-AuNSs from TEM images. Day zero represents as-synthesized F-AuNSs before they were delivered in mice. The TEM images used for surface area calculation included that of spleen, liver, and kidneys harvested 7-, 45-, and 90-days after (i) IP and (ii) IV delivery of F-AuNSs. The error bars were calculated by counting the average surface area of >100 single particles per group from TEM images.

Next, we assessed the long-term impact of F-AuNSs by determining Au content in various organs with ICP-MS retrieved at 1-, 7-, 30-, 45-, and 90-days after a single IP or IV injection (Figure 3e; Figures S7b and S8b, Supporting Information). Our results showed the maximum accumulation time point of F-AuNSs varied in different organs, and both IP and IV delivery showed similar trends (except in the pancreas) indicating the route of administration had minimal impact in the long-term biodistribution. In the spleen, maximum accumulation of F-AuNSs occurred at 30 days followed by rapid clearance but some retention at 90 days. In the liver, maximum accumulation of F-AuNSs was found at 45 days followed by slow clearance up to 90 days. Our results showed that i) the conjugation of various ligands including PEG resulted in prolonged circulation of F-AuNSs and ultimate accumulation in the MPS organs, and ii) F-AuNSs were cleared through the MPS via hepatobiliary excretion and not through recirculation into blood, consistent with previous observations in the literature.^[39,40]^ We found significant accumulation of F-AuNSs in the kidneys comparable to that in the liver likely attributable to multiple factors: first, the densely packed PEG chains and well functionalized surface of F-AuNSs improved in vivo stability and blood circulation that enabled entry in the kidneys. Second, the ≈95 nm size of F-AuNSs resulted in accumulation in the mesangium via glomerular endothelial fenestrae and promoted kidney accumulation.^[41]^ This finding is consistent with the literature that supports NPs undergoing renal filtration must pass through 1) glomerulus endothelium containing pores (fenestra) of 80–100 nm, 2) the negatively charged glomerular basement membrane with pores of 5–8 nm, and 3) a slit diaphragm of podocytes with pores of ≈15 nm.^[42,43]^ We noted that F-AuNSs did break down into smaller gold NPs in major organs (see Figure 4) but elimination through the glomeruli remained slow and thus kidney accumulation was observed even at 90 days.

Biodistribution of F-AuNSs in the lungs and heart showed higher accumulation when delivered IV consistent with literature findings,^[44]^ and significant Au content within 24 h postinjection when F-AuNSs were in active circulation. These findings supported our toxicity analysis (Figure 2) indicating F-AuNSs should have minimal lung toxicity and caused no adverse cardiovascular effects critical to translation of these NPs. We also observed IP delivery of F-AuNSs resulted in a significantly higher accumulation in the pancreas relative to IV delivery, which peaked at 30 day postdelivery and then slowly cleared. High distribution of NPs in pancreas via IP administration likely arose from intraperitoneal circulation and uptake by tissue-resident and peritoneal macrophages which honed to the pancreas.^[45]^ F-AuNSs also showed higher uptake in the stomach via IP delivery indicating they penetrated via the mucus layer and were absorbed by gastrointestinal epithelial cells and stable enough to be retained against rapid gastric emptying. High stomach content of Au may also be contributed by mice engaging in coprophagia of excreted F-AuNSs. Additionally, brain, bone, and muscles had minimal Au, which decreased over time although at different rates (Figure S7b, Supporting Information). These results were expected as NPs > 10 nm cannot pass through the blood-brain-barrier and have limited transvacular transport through the small fenestrations in bone and muscle. Uptake of F-AuNSs in the intestines could be through enterocytes in the upper small intestine or through passive uptake during cell turnover.^[46,47]^ Further, biodistribution of F-AuNSs in the blood for both injection routes showed rapid clearance from 1 day to 7 days followed by a steady and slow decrease beyond 7 days (Figure S8b, Supporting Information). The results indicated that F-AuNSs were gradually transported from the blood to other organs after 7 days postdelivery. We also observed that in the urine F-AuNSs increased over time which may be contributed by the breakdown of F-AuNSs and steady clearance through the kidneys. The maximum clearance of F-AuNSs through feces occurred at 7 days postinjection for both routes of delivery, followed by a slower clearance over time (Figure S8b, Supporting Information). Since our results showed that F-AuNSs are present both in the feces and the urine, data suggested that F-AuNSs were most likely cleared through both the MPS via hepatobiliary excretion and the kidneys through urinary excretion.

**Scheme 1.**
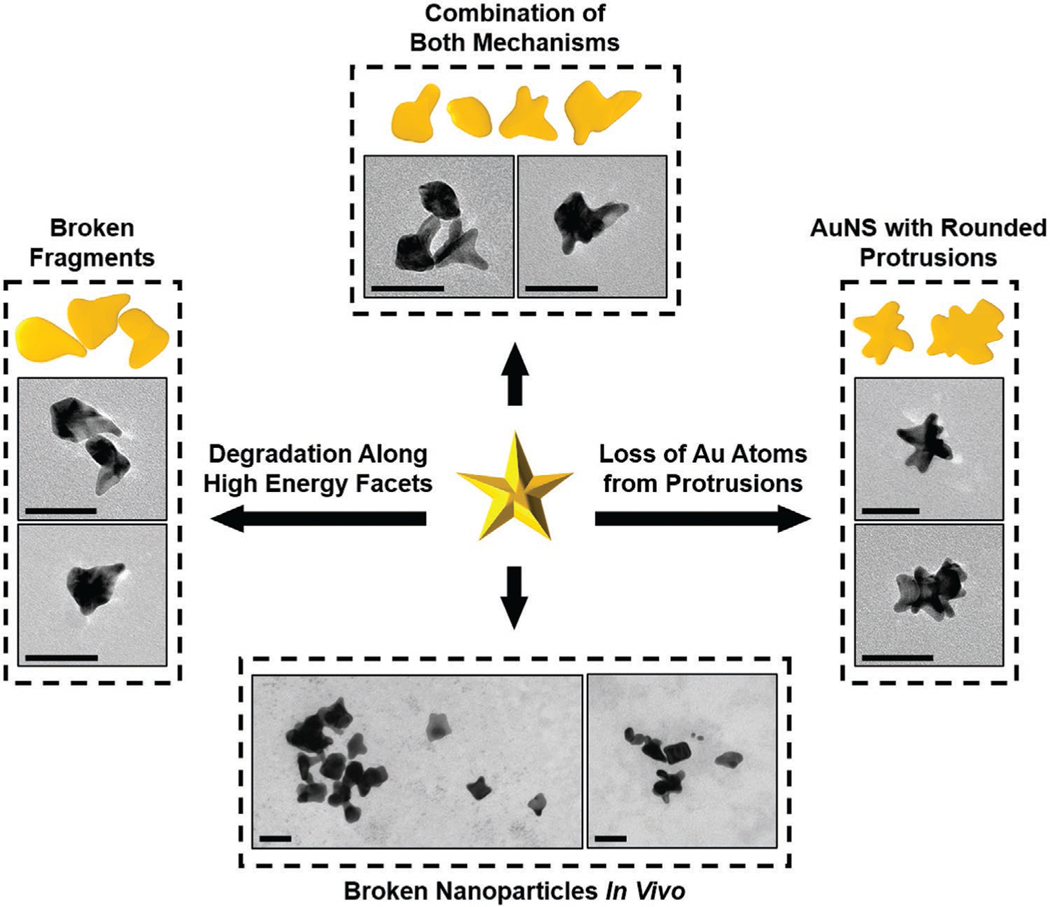
Schematic illustration showing mechanistic evolution of F-AuNSs degradation in ALF and in vivo. Scale bar is 50 nm.

We then examined the morphology of F-AuNSs from 7 to 90 days in tissues to determine cellular transcytosis and in vivo clearance in the long-term. TEM of spleen of mice that received F-AuNSs (0.04 mg g^−1^) via IP and IV (Figure 4a) showed for the first time the surprising breakdown of F-AuNSs in vivo as early as 7 days postinjection. The breakdown of F-AuNSs delivered via IP began with nanostar protrusions breaking off which ultimately transformed into smaller sphere-like NPs with fewer short branches (Figure 4a). The reshaping of F-AuNSs from star to sphere-like shape was expected given the higher thermodynamic stability of spherical morphology. This result suggested that for a given size, anisotropic nonspherical NPs should have a rapid breakdown in vivo enabling faster clearance. F-AuNSs were primarily found in splenic macrophages located in the endosome/lysosome indicating that F-AuNSs were endocytosed consistent for gold NPs.^[48,49]^ The morphological transformation of F-AuNSs was likely catalyzed by the acidic and enzymatic degradation in endosomes/lysosomes. F-AuNSs delivered via IV demonstrated similar shape deformation in splenic macrophages but the degradation started after the 7-day time point (Figure 4a) suggesting that F-AuNSs stayed in circulation longer when delivered via IV. To further confirm that the morphological transformation of F-AuNSs was triggered by the acidic and enzymatic environment in endosomes/lysosomes, we conducted a series of ex vivo degradation experiments by exposing F-AuNSs to artificial lysosomal fluid (ALF) for up to 21 days and then examining changes in optical properties (via UV–vis spectroscopy) and morphology (via TEM). ALF contains 13 inorganic and organic components and has been previously used to simulate the endosomes/lysosomes environment in terms of pH, ionic strength, multiple components, and viscosity.^[50–52]^ The F-AuNSs in ALF solution was placed on a thermomixer at 60 rpm and 37 °C and maintained at that temperature during the entire study. We observed that the intensity in the extinction spectra of F-AuNSs decreased over time (Figure S9a, Supporting Information) but the FWHM peaked at 5 days and then slowly decreased over time (Figure S9b, Supporting Information). Changes in optical properties of F-AuNSs in ALF suggested alterations in shape and size as a function of time. Therefore, we evaluated the morphological changes of F-AuNSs in ALF at 7, 14, and 21 days with TEM (Figure S10, Supporting Information). TEM images suggested deformation and disintegration of F-AuNSs and a drastic decrease in surface area with longer incubation time in ALF (Figure S11, Supporting Information). Based on these TEM images (i.e., decrease in surface area, and images obtained in vivo), we hypothesize that the breakdown of F-AuNSs likely occurred through a combination of two mechanistic pathways. These pathways include 1) degradation along high energy facets of F-AuNSs where the longest protrusions, which were thermodynamically least stable, fragmented off over time resulting in irregular-shaped F-AuNSs particles, and 2) the loss of Au atoms from the protrusions such that the protrusions lost their “sharpness” resulting in gradual rounding of the protrusions. Scheme 1 shows an illustration and supporting TEM images of the breakdown of F-AuNSs. Collectively, these longitudinal analyses in ALF confirmed our in vivo findings that the degradation of F-AuNSs was likely triggered by the acidic and enzymatic environment in endosomes/lysosomes.

We also investigated the morphological changes of F-AuNSs in the excised liver where independent of the route of delivery, F-AuNSs primarily accumulated in Kupffer cells, the liver-resident macrophages, consistent with our previous findings of AuNSs^[49]^ (Figure S12, Supporting Information). The number of Kupffer cells and their corresponding function vary in different zones of the hepatic lobules where Kupffer cells found in the periportal zones have a higher phagocytic activity.^[53,54]^ Whereas prior studies have indicated spherical gold NPs are cleared via endothelial cells and hepatocytes of liver,^[40]^ we did not observe F-AuNSs in either of these cell types suggesting both shape and surface properties determined where they accumulated and how they exocytosed. Unexpectedly, the breakdown of F-AuNSs in the liver was much slower than the spleen suggesting Kupffer cells had a slower turnover than splenic macrophages. Similar to liver, in the kidneys F-AuNSs showed slow breakdown independent of the route of administration and gradually reshaped into sphere-like NPs between 7 and 45 days postdelivery (Figure S13, Supporting Information). F-AuNSs were primarily located in the glomeruli or interstitial cells around the proximal convoluted tubules indicating intact F-AuNSs (≈95 nm) permeated through the 80–100 nm pores of the fenestrated glomerular endothelia but did not pass through pores of glomerular basement membrane (5–8 nm) and slit diaphragm (≈15 nm). A few nonclustered F-AuNSs did transiently enter the mesangium at earlier time-points but retention over longer timescales was not observed due to lack of phagocytosis by mesangial cells. However, after breakdown smaller broken F-AuNSs were found in interstitial cells (90-day time point, Figure S13, Supporting Information). These findings supported our ICP-MS results, which clarified the presence of F-AuNSs in the kidneys long-term. To confirm that the observed particles in TEM images of tissues were AuNSs, we measured the diffraction pattern that showed polycrystalline Au planes (Figure S14, Supporting Information). To quantify the breakdown of F-AuNSs as a function of the route of administration in the major organs, we measured the surface area of >100 single particles in TEM images at each time point with ImageJ and compared that to as-prepared F-AuNSs before injection (Figure 4b). Our results showed a dramatic decrease in the surface area of F-AuNSs as they broke down to smaller pieces in vivo in spleen, liver, and kidneys independent of the route of delivery but the rate of breakdown varied in each tissue.

Collectively, the biodistribution studies and morphological analysis suggested that in vivo uptake, localization, and clearance of F-AuNSs were likely controlled by three factors: intracellular trafficking, blood opsonization, and transcytosis of the “broken” NPs that resulted from the degradation of F-AuNSs. We performed endocytosis and intracellular trafficking in vitro studies to explain the accumulation F-AuNSs in specific organelles such as the presence of F-AuNSs in endosome/lysosome of macrophages in TEM images of spleen. Since the bioavailability of nanoparticles is directly governed by their endocytosis, understanding if functionalization of AuNSs activates a specific pathway over others could ultimately explain patterns in the in vivo biodistribution. Further, we performed ex vivo mechanistic studies of F-AuNSs interaction with serum proteins and protein corona formation, since blood opsonization is an important parameter in controlling the in vivo fate of nanoparticles. Finally, by observing the breakdown of F-AuNSs in TEM images in vivo, we questioned what the fate of the “broken” NPs would be and what role their shape and surface properties would play in transcytosis across membranes and ultimately clearance of F-AuNSs. These questions involve complex and expansive experiments that are beyond the scope of this work. Therefore, we chose to do Martini CG simulations to mimic the experimental conditions and understand how the properties of these broken particles will control the fate of F-AuNSs long term.

**Figure 5.**
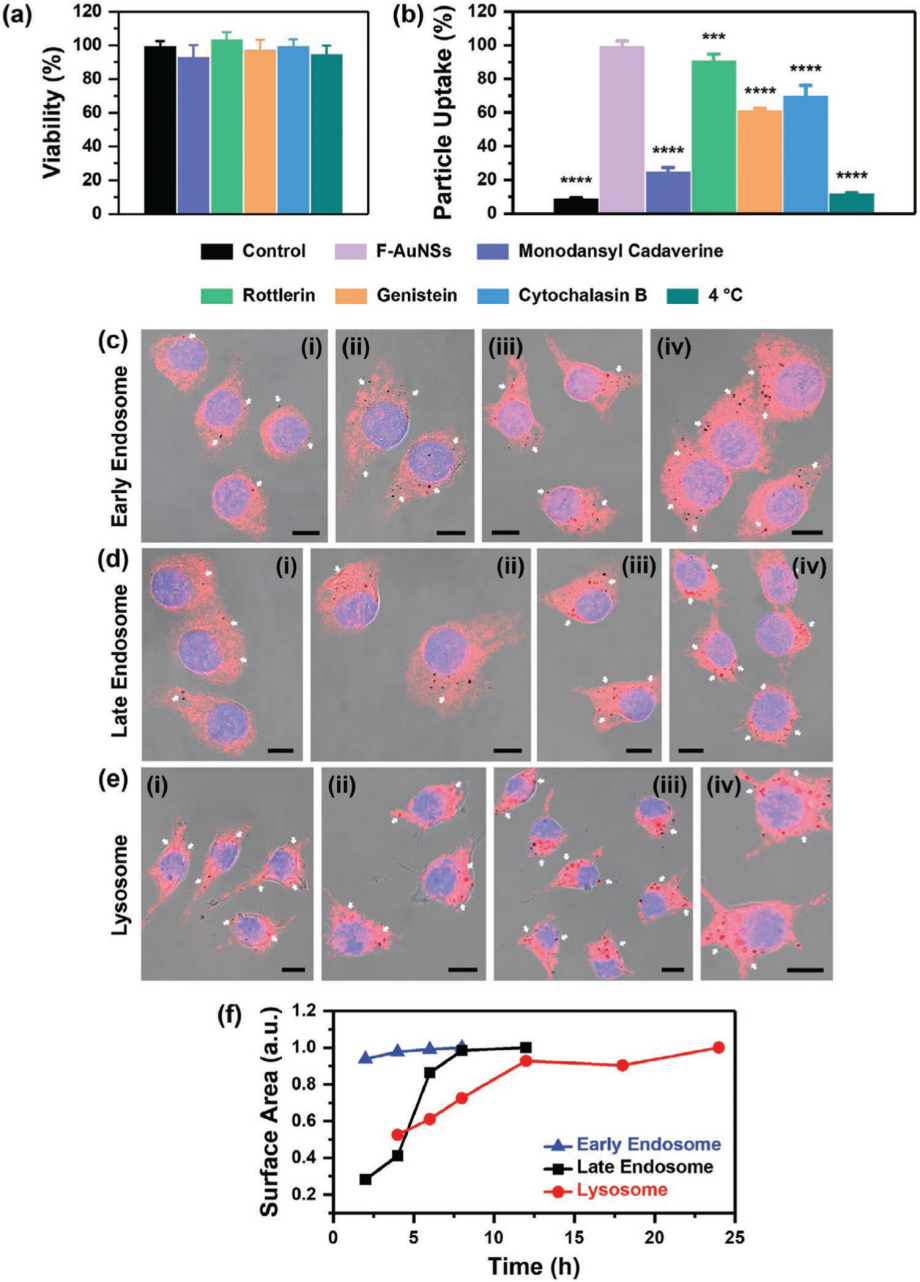
Endocytosis pathway dependence and intracellular trafficking of F-AuNSs in vitro. a) MTT cell viability assay of J774A.1 incubated for 8 h with inhibitors of the different endocytosis pathways including monodansyl cadaverine (200 μm), rottlerin (2 μm), genistein (200 μm), cytochalasin B (10 μg mL^−1^), and cells incubated at 4 °C. These cells did not receive any F-AuNSs. Black bar represents control cells that did not receive any inhibitors. Cell viability was measured at 540 nm. All data were presented as mean ± standard deviation (*n* = 5 per concentration and *N* = 2 independent experiments). b) Endocytosis of F-AuNSs in J774A.1 incubated with different inhibitors including monodansyl cadaverine (200 μm, 10 min) for clathrin-mediated endocytosis, rottlerin (2 μm, 30 min) for macropinocytosis, genistein (200 μm, 1 h) for caveolae-mediated endocytosis, cytochalasin B (10 μg mL^−1^, 2 h) for phagocytosis and 4 °C (1 h) for all energy-dependent uptake pathways, respectively. Cells without F-AuNSs and without inhibitor were denoted as “Control” (negative control), and incubated with only F-AuNSs and without any inhibitors at 37 °C are marked as “F-AuNSs” (positive control). Here, all data were represented as mean ± standard deviation (*n* = 4 per group and *N* = 2 independent experiments). * indicates *p* < 0.05, *** indicates *p* < 0.001, **** indicates *p* < 0.0001 versus “F-AuNSs” (positive control). Colocalization of F-AuNSs with c) early endosome shown as red fluorescence for (i) 2, (ii) 4, (iii) 6, and (iv) 8 h, d) late endosome shown as red fluorescence for (i) 2, (ii) 4, (iii) 6, and (iv) 8 h, and e) lysosome shown as pseudocolored red for (i) 4, (ii) 8, (iii) 18, and (iv) 24 h in J774A.1. The nucleus in each cell was stained with DAPI (blue). Scale bar is 10 μm. White arrows point to particles. f) Normalized total surface area of particles (black dots, quantified with ImageJ) colocalized with early endosome, late endosome, and lysosome in 50 cells.

It is well known that in vivo fate of NPs is controlled by their cellular-level endocytosis pathway broadly described into clathrin-mediated endocytosis, macropinocytosis, caveolae-mediated endocytosis, and phagocytosis.^[55–58]^ Briefly, i) clathrin-mediated endocytosis dominates most receptor–ligand binding and traffics NPs from endosomes to lysosomes through clathrin-coated pits; ii) macropinocytosis is a nonspecific pathway and transports NPs to macropinosomes; iii) caveolae-mediated pathway translocates NPs to Golgi apparatus or endoplasmic reticulum; and iv) phagocytosis, the primary mechanism of engulfment by macrophages and other phagocytes, is initiated after protein corona formation and opsonization of NPs. Here, we assessed the uptake mechanism of F-AuNSs in two macrophage cell lines (J774A.1 and RAW 264.7) where cells were preincubated with inhibitors that blocked the different pathways prior to incubating with F-AuNSs. Monodansyl cadaverine inhibited clathrin-mediated endocytosis,^[59]^ macropinocytosis was inhibited with rottlerin,^[60]^ Genistein inhibited caveolae-mediated endocytosis,^[61]^ and cytochalasin B was used to inhibit phagocytosis.^[62]^ Cells were also exposed to 4 °C but without inhibitors to determine whether F-AuNSs uptake followed an energy-dependent process. MTT cell viability assay indicated that inhibitors did not have toxic effect on cells at the concentrations and incubation time used (Figure 5a; Figure S15a, Supporting Information). Quantitative analysis using flow cytometry showed that F-AuNSs uptake was dominated by clathrin-mediated endocytosis in both cell lines, and followed energy-dependent internalization (Figure 5b; Figure S15b, Supporting Information) consistent with literature findings where AuNPs were primarily taken up by clathrin-mediated pathway.^[63]^ However, we found that other pathways also contributed to F-AuNSs uptake in cells suggesting that unlike PEG-coated NPs, the characteristics of the ligands functionalized on F-AuNSs likely promoted high in vitro uptake via multiple intracellular pathways. To further understand the uptake mechanism of F-AuNSs, we treated J774A.1 cells with transferrin to block clathrin-mediated endocytosis pathway, with dextran to block macropinocytosis, and with cholera toxin subunit B (CTB) to block caveolae-mediated endocytosis pathway (Figure S16, Supporting Information). We hypothesized that inhibiting cell surface markers of endocytosis with these proteins will not only impact those specific pathways but also alter cellular uptake through other pathways as the cells try to compensate for the loss of endocytosis capacity through the pathways that were blocked.^[64]^ We observed that F-AuNSs colocalized with cells treated with dextran but minimal with transferrin and CTB confirming the trends in Figure 5b that F-AuNSs were primarily uptaken by clathrin-mediated endocytosis as well as by caveolae-mediated endocytosis, but minimal uptake occurred through macropinocytosis. The high uptake of F-AuNSs observed when macropinocytosis pathway was blocked with dextran also supported our hypothesis that cellular uptake was transformed when this pathway was blocked as cells tried to compensate for loss of endocytosis capacity. We then investigated the longitudinal intracellular trafficking of F-AuNSs in different cellular organelles including early endosomes, late-endosomes, and lysosomes. Cells were incubated with F-AuNSs for 24 h followed by labeling with anti-EEA1 antibody and anti-RAB7 antibody for early and late endosome staining, respectively. F-AuNSs colocalized with early endosome near the cellular membrane (Figure 5c; Figures S17a and S18a, Supporting Information) while with late endosome F-AuNSs were distributed in the cytoplasm and around the nuclei (Figure 5d; Figures S17b and S18b, Supporting Information). Cells stained with a selective lysosomal dye, BioTracker NIR 633, showed that F-AuNSs colocalized with lysosome primarily around the perinuclear region, which suggested F-AuNSs were eventually transported to lysosomes (Figure 5e; Figures S17c and S18c, Supporting Information). We also quantified the total surface area of particles accumulated in 50 cells (using ImageJ) that were colocalized with each of these organelles in both macrophage cells lines as a function of time (Figure 5f; Figure S19, Supporting Information). Our longitudinal study revealed that F-AuNSs colocalized with early endosomes within the first 4 h of incubation, then transported to late endosome in the 6–12 h regime followed by migration to lysosomes which peaked at ≈12 h postincubation and remained there until 24 h. These compelling in vitro results supported our in vivo findings where F-AuNSs were observed in endosomal/lysosomal compartments in splenic macrophages and Kupffer cells in the liver.

**Figure 6.**
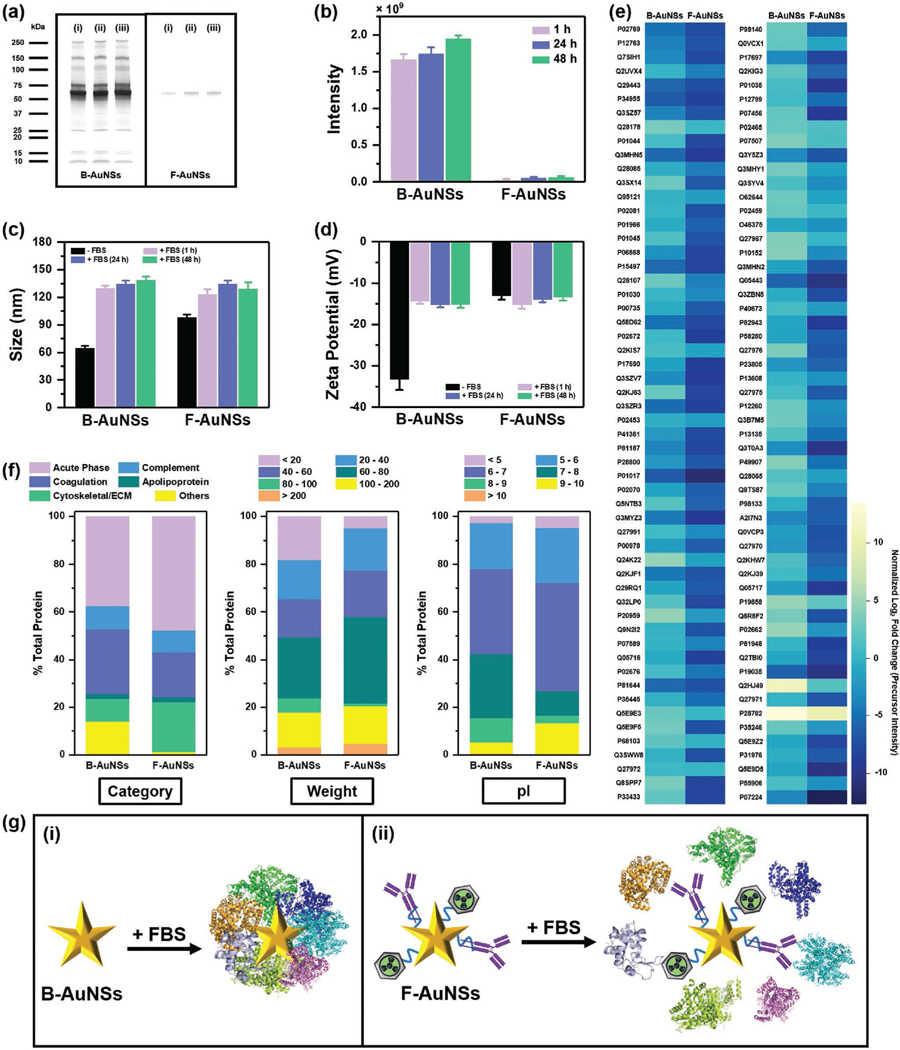
Proteomic study of surface protein corona. a) B-AuNSs and F-AuNSs were treated with 60% FBS for (i) 1, (ii) 24, and (iii) 48 h and the serum proteins immobilized on the surface of nanoparticles were determined by SDS-PAGE. b) Quantification of band intensity. c) Size and d) surface charge analysis of B-AuNSs and F-AuNSs after incubation with 60% FBS. All data were represented as mean ± standard deviation (*n* = 4). e) Classification of protein corona components identified by quantitative LC-MS/MS. A total of 213 proteins were identified and the 112 most abundant proteins were presented in the heat map. f) Proteins adsorbed on B-AuNSs and F-AuNSs were classified by category, molecular weight (kDa) and pI. g) Schematic representation of (i) B-AuNSs and (ii) F-AuNSs with serum proteins.

Next, we wanted to understand if the functionalization of AuNSs minimized opsonization and protein corona (PC) formation, which ultimately impacts their clearance in vivo. PC formation modulates nanoparticles’ physicochemical properties and compromises their transport, targeting, and endocytosis in vivo.^[65,66]^ Therefore, strategies are now explored to minimize opsonization via surface functionalization enabling nanoparticle “cloaking” from macrophages in MPS and promoting colloidal stability and prolonged blood circulation in vivo.^[10,11,67]^ Here, we incubated both B-AuNSs and F-AuNSs with 60% fetal bovine serum (FBS), to represent serum proteins in vivo, for 1, 24, and 48 h at 37 °C followed by thorough purification to remove unbound proteins. Sodium dodecyl sulfate polyacrylamide gel electrophoresis (SDS-PAGE) visualized by silver staining confirmed minimal free proteins in the supernatant after purification (Figure S20a–c, Supporting Information). SDS-PAGE images and quantification of band intensities (Figure 6a,b) confirmed significant PC formation on negatively charged B-AuNSs, within 1 h of incubation, relative to near neutrally charged F-AuNSs indicating that the properties and density of surface ligands on F-AuNSs minimized adsorption of serum proteins. PC formation on B-AuNSs manifested as a significant increase in size by ≈105% and an increase in surface charge from −35 to −15 mV (Figure 6c,d; Figure S20d,e, Supporting Information). Comparatively, F-AuNSs had ≈30% size increase and minimal change in surface charge postincubation with FBS supporting the SDS-PAGE results. We also incubated both B-AuNSs and F-AuNSs for 24 h at 37 °C with 60% mouse serum obtained from C57BL/6 mice (Figure S21, Supporting Information). Both size and surface charge of B-AuNSs and F-AuNSs in mouse serum showed similar trends as that observed in 60% FBS indicating that FBS effectively serves as a “model serum” to characterize serum proteins and protein corona formation on NPs. Further, we investigated the composition of serum proteins adsorbed on B-AuNSs and F-AuNSs by using liquid chromatography tandem mass spectrometry (LC-MS/MS). A total of 213 proteins were identified and the 112 most abundant proteins were represented as normalized relative abundance for each type of AuNSs showing significantly fewer proteins were adsorbed on F-AuNSs (Figure 6e). We further classified the adsorbed proteins into the type of proteins, molecular weight (MW), and isoelectric point (pI), and noted that F-AuNSs had high adsorption of acute-phase proteins and lower coagulation proteins relative to B-AuNSs (Figure 6f). These proteins directly impacted in vivo circulation of nanoparticles and uptake by MPS^[68]^ and this observation supported our biodistribution results that F-AuNSs had prolonged blood circulation with slower uptake in spleen and liver that peak ≈30–45 days. We also observed high cytoskeletal and extracellular matrix (ECM) proteins adsorbed on F-AuNSs; ECM proteins such as collagen and fibronectin regulate cell adhesion, proliferation, and migration. Nanoparticles preadsorbed with ECM proteins enhanced cell adhesion and migration,^[69]^ which supported our endocytosis results (Figure 5) that F-AuNSs were highly efficient in intracellular uptake and trafficking relative to B-AuNSs. Our results also showed that B-AuNSs with size of ≈65 nm had a strong affinity for low MW proteins <20 kDa, which is consistent with literature where 40 nm AuNSs have shown high interactions with low MW proteins.^[70]^ F-AuNSs, with larger size of ≈95 nm, adsorbed a greater fraction of higher MW proteins, a trend supported by other literature findings.^[12]^ Lastly, classification of proteins based on pI showed that B-AuNSs adsorbed approximately threefold proteins with a pI between 7 and 9 compared to F-AuNSs, whereas F-AuNSs adsorbed approximately threefold proteins with a pI between 9 and 10 relative to B-AuNSs. These results demonstrated that anionic B-AuNSs preferentially adsorbed positively charged proteins, and near-neutral F-AuNSs adsorbed negatively charged proteins (i.e., protein absorption was mediated by electrostatic interactions on AuNSs). The % protein adsorption of each type is presented in Table S1 of the Supporting Information. Collectively, our analysis showed that the ligands functionalized on F-AuNSs minimized overall PC formation (Figure 6g), and the proteins that were adsorbed mediated in vivo blood circulation and intracellular uptake supporting our long-term biodistribution results.

**Figure 7.**
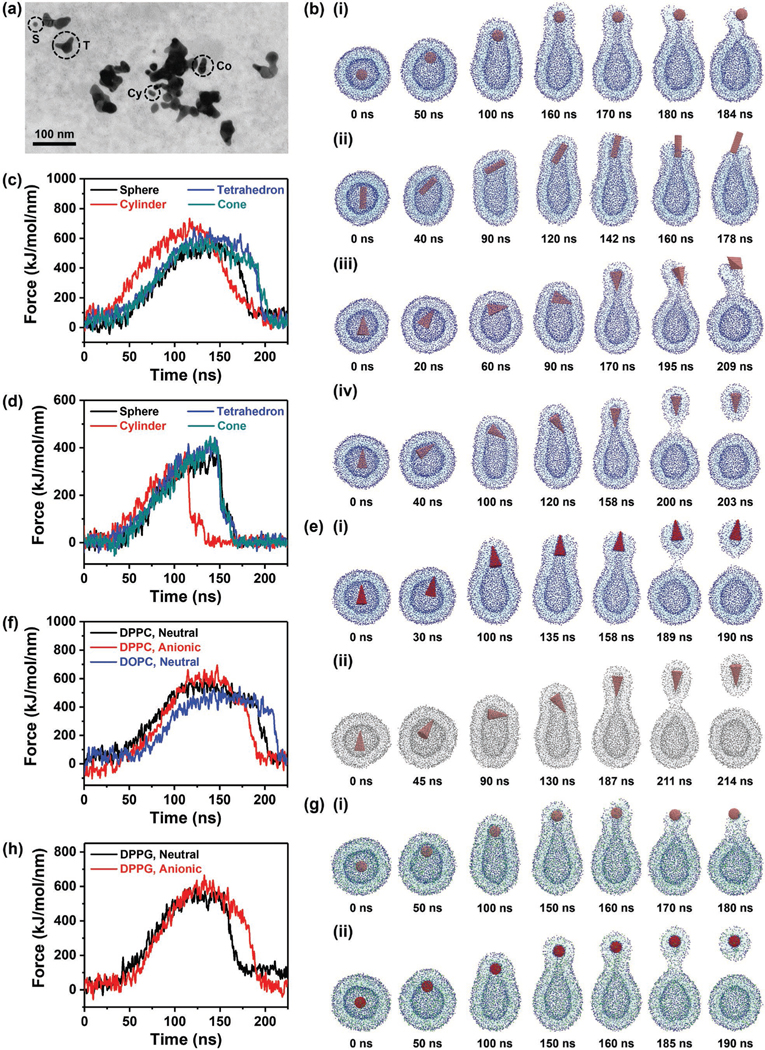
Martini CG simulation of nanoparticle translocation across membrane. a) Representative TEM images of broken NPs. Here, *S*, *T*, *Cγ*, and *Co* represent sphere, tetrahedron, cylinder, and cone, respectively. b) Snapshots of NPs (volume ≈ 35 nm^3^) that represent the “broken NPs” that resulted from F-AuNSs disintegration in vivo with varying shapes, and their translocation across the model DPPC lipid membranes. The interaction force between c) large- (volume ≈ 35 nm^3^) or d) small-sized (volume ≈ 4.5 nm^3^) NPs with different shapes and DPPC lipid membrane as a function of time. e) Snapshots of translocation of (i) anionic cone-shaped NPs across the model cell membranes composed of DPPC lipids and (ii) neutral cone-shaped NPs across the cell membrane composed of DOPC lipids. f) The interaction force between the neutral (black) or anionic (red) NPs and DPPC membrane as a function of time, and between the neutral NPs and membrane composed of DPPC (black) or DOPC (blue) as a function of time. g) Translocation of (i) neutral and (ii) anionic spherical NPs (volume ≈ 35 nm^3^) across the model cell membrane composed of DPPC mixed with DPPG lipids at a ratio of 3:1. h) The interaction force between the neutral or anionic spherical NPs and mixed DPPC–DPPG lipid membranes as a function of time.

Our in vivo results showed that F-AuNSs disintegrated to broken NPs (Figures 4a and 7a), and the physicochemical properties of these NPs ultimately controlled the clearance of F-AuNSs in vivo. To understand the transcytosis of these broken NPs across the cell membrane that would ultimately result in cellular exocytosis and clearance in vivo, we simulated the interaction between the NPs and membrane lipid bilayer using the Martini CG method. This model features four main types of CG bead: polar (P), intermediately polar (N), nonpolar (C), and charged (Q).^[71]^ Here, each CG bead is represented by four heavy atoms and associated hydrogens. By decreasing the degrees of freedom of the system through atom grouping, our simulations were performed at longer timescales to mimic physiological phenomena,^[72]^ as a function of shape, size, and charge of NPs. In these simulations, four different shapes with an equivalent volume (≈35 nm^3^) were employed including sphere, cylinder, tetrahedron, and cone (Figure S22a, Supporting Information); and these shapes matched those of the broken NPs seen in the TEM images (Figures 4a and 7a). To understand the timescale of transcytosis in the model, external force was applied to the center-of-mass of NPs in the *z* direction (Figure 7b). Our model lipid membrane was composed of zwitterionic dipalmitoylphosphatidylcholine (DPPC),^[73–75]^ representing phospholipids in cell membranes. During the translocation, the membranes conformed to the shape of NPs where isotropic NPs (sphere) did not show orientation-dependence but anisotropic NPs were oriented with the flat side toward the membrane to induce less membrane deformation, which was thermodynamically favorable for transcellular transport.^[76,77]^ At longer timescales, the NPs reoriented until the long axis was perpendicular to the membrane in preparation for membrane transcytosis. The interaction force between the NPs and membrane as a function of time revealed that cylindrical NPs translocated faster than other shapes (Figure 7c) likely due to the small surface area, 4.5 nm^2^, along the short axis of cylinders that induced small adhesion forces on the membranes.^[76]^ Cones and tetrahedrons with larger surface area along the short axis, 12.5 and 13.0 nm^2^, respectively, induced higher adhesion, bending, and tension forces on the membrane requiring longer timescales.^[78,79]^ In addition to the shape, transcytosis was impacted by the NP size. Here, the size of all four shapes was varied from larger (35 nm^3^) to smaller (4.5 nm^3^) dimensions while keeping the equivalent volume for each shape similar (Figure S22b, Supporting Information). As expected, smaller NPs exerted less force on the membrane (≈600 kJ mol^−1^ nm^−1^ for large-sized vs 400 kJ mol^−1^ nm^−1^ for small-sized NPs) and had faster transcytosis likely attributable to translocation via pores, and minimal interactions with the lipid membrane consistent with literature findings (Figure 7d).^[80,81]^ Cylindrical smaller NPs displayed fastest transcellular transport (Figure S23, Supporting Information) attributed to twofold less number of contact with the membrane relative to other shapes, and faster timescales in achieving the membrane contact (Figure S24, Supporting Information). These simulations suggested that NPs modulated the membrane deformation dynamics based on their morphology and directly controlled membrane interactions and the likelihood of rapid in vivo clearance.

We also hypothesized that the disintegration of F-AuNSs likely altered the surface charge of the resulting broken NPs such that they were no longer near-neutral as F-AuNSs but anionic as B-AuNSs (Figure 1e) as we expected the ligands to likely be stripped off the surface. To understand the effect of surface charge, we simulated larger-sized (35 nm^3^ volume) NPs with cone shape featuring neutral and anionic surface charges (Figure 7b–iv,e–i). Simulations showed that cone-shaped NPs proceeded through a laying-down-then-standing-up sequence. Further, we observed that a small neck was created when the long axis of anionic NPs underwent transcytosis. The simulation proceeded through formation of membrane-bound vesicles which budded off the cell membrane followed by NP entrapment in the vesicles, and finally NP wrapped vesicles separated from the parent cells and translocated out of the membrane. This observation was unique to cone-shaped NPs (Figure 7b) as other shapes did not leverage membrane-bound vesicles to enable transcellular transport for the DPPC model lipid membrane. The simulations showed two salient features: i) relative to anionic cone NPs, neutral NPs changed their orientation by 180° during transcytosis, and ii) neutral cone NPs required longer timescale to exit out of the membrane (Figure 7f). These results suggested that when F-AuNSs disintegrated in vivo, if the resulting broken cone-shapes retained PEG and other ligands on their surfaces, transcellular transport of such neutral NPs was determined by their specific orientation. In the case that ligands were stripped-off from the broken cone-shapes during in vivo dynamics, such anionic NPs may undergo faster clearance facilitated by the sharp corner of the cone which had fewer number of contacts with the membrane and can propel the vesicle to separate from the parent cell.^[79]^ We also examined the impact of the composition of the membrane in NP transcytosis since membrane lipid composition is tissue-specific^[82]^ and may explain our in vivo results where breakdown and clearance of AuNPs was slower in the liver relative to the spleen (Figure 4a; Figure S12, Supporting Information). Here, translocation of neutral cone-shaped AuNPs (35 nm^3^) across DPPC lipid membrane (Figure 7b–iv) was compared to dioleoylphosphatidylcholine (DOPC) membrane, which showed slower transcytosis across the DOPC membrane (Figure 7e–ii,f). DOPC has higher molecular weight and is softer than DPPC with two double bonds on the fatty acid tail (Figure S25, Supporting Information). Our simulations suggested that the stiffness of the DOPC membrane impacted the interaction forces with neutral NPs (540 kJ mol^−1^ nm^−1^ for DOPC vs 600 kJ mol^−1^ nm^−1^ for DPPC) requiring longer timescale for the NPs to change orientation and enable the vesicles to bud off the membrane. Additionally, considering the natural negative charge of cell membranes, we simulated a membrane composed of DPPC and dipamitoylphosphatidylglycerol (DPPG) at a 3:1 ratio with a net negative surface charge. DPPG is an anionic lipid with identical structure as DPPC except a glycerol end-group instead of choline end-group (Figure S25, Supporting Information). Neutral and anionic spherical NPs (≈35 nm^3^ volume) translocation across the DPPC–DPPG membrane showed that anionic NPs were entrapped in membrane-bound vesicles and had slow transcellular transport attributable to the electrostatic repulsion with negatively charged membrane (Figure 7g,h). These results were also translated in 4 nm^3^ smaller spherical NPs (Figure S26, Supporting Information). Finally, since NPs tend to form aggregates upon exposure to biological environment, we evaluated the effect of aggregation in NP transcytosis by constructing an aggregated NP which comprised of five sphere AuNPs with a diameter of 2 nm each and connected together by covalent bonds resulting in an overall diameter of 4 nm (Figure S27, Supporting Information). We featured both neutral and anionic surface charge for these aggregated NP constructs. We compared the transcytosis of aggregated NP that has similar size and volume as a monodisperse single spherical NP (4 nm diameter, and ≈35 nm^3^ volume) using DPPC or DPPC–DPPG lipid membranes. Specifically, we compared the following four scenarios: 1) aggregated versus single spherical AuNPs with a neutral surface charge in the DPPC lipid membrane, 2) aggregated versus single spherical AuNPs with an anionic surface charge in the DPPC lipid membrane, 3) aggregated versus single spherical AuNPs with a neutral surface charge in the mixed DPPC–DPPG lipid membrane, and 4) aggregated versus single spherical AuNPs with an anionic surface charge in the mixed DPPC–DPPG lipid membrane. Our results showed that irrespective of NP surface charge or lipid membrane composition, aggregated and single spherical AuNPs showed similar transcytosis behavior. These results indicated that if the broken NPs form aggregates in vivo and the size and overall volume of the aggregates are similar to those of intact monodisperse NPs, then aggregation would not alter the transcytosis process (Figures S28 and S29, Supporting Information).

## 3. Conclusion

In summary, this work addresses a long-standing question in nanomedicine on the long-term fate of gold NPs in vivo and how morphology, surface properties, and route of administration simultaneously contribute to in vivo breakdown and ultimate clearance. Here, we evaluated both the in vitro characteristics and in vivo fate of F-AuNSs up to 90 days post IP and IV delivery. Our findings have several implications. 1) The route of administration had significant impact on the tissue-specific biodistribution of F-AuNSs with maximum difference in the first 24 h postinjection but also observable up to 60 days after delivery. We concluded that whereas IV delivery was preferred for targeting most organs, IP delivery was ideal for delivery to the pancreas (and stomach) that may be leveraged for treating disorders specific to these organs. 2) We also concluded that both the anisotropic star shape and surface ligands directed splenic clearance for F-AuNSs. This finding is important as compared to AuNSs, spherical AuNPs have 3× higher accumulation in liver.^[83–85]^ Further, F-AuNSs retention in the mesangium of kidneys may enable glomerular targeting, longitudinal imaging, and image-guided therapies in the kidneys. 3) Our in vitro results indicated that the nanoparticle surface property was a driving factor in reducing opsonization and the type of proteins adsorbed, and in enabling multiple endocytosis pathways for ultimate trafficking in endosomes/lysosomes where F-AuNSs were disintegrated in the acidic environment. Finally, 4) we showed for the first time that the anisotropic shape of F-AuNSs propelled their breakdown in vivo which starts as early as 7 days postinjection into sub-20 nm broken nanoparticles. Martini CG simulations suggested that transcytosis of broken nanoparticles was size-, shape-, and surface charge-dependent, and directed by the composition of lipid membranes, which supported the in vivo differences we observed in liver versus spleen. We envision that our findings will ultimately guide researchers in designing advanced nanomaterials where multiple physicochemical properties and route of in vivo delivery must be simultaneously manipulated to direct tissue-specific targeting.

## 4. Experimental Section

### Materials:

Gold(III) chloride trihydrate (HAuCl_4_·3H_2_O), HEPES, FBS, dimethyl sulfoxide (DMSO), sodium chloride, sodium hydroxide, citric acid, calcium chloride, glycine, sodium citrate dihydrate, cytochalasin B, genistein, rottlerin and monodansyl cadaverine, dithiothreitol (DTT), and *β*-mercaptoethanol were purchased from Sigma-Aldrich. The Milli-Q water (18 MΩ) was obtained from a Milli-Q Direct-Q 3UV system. OPSS-PEG-SVA-2000 and NH_2_-PEG-SH-5000 were purchased from Laysan Bio. IgG2a antibodies (BE0089, Clone 2A3) were purchased from Bio X cell. Sodium bicarbonate (NaHCO_3_), hydrochloric acid (HCl), nitric acid (HNO_3_), PBS, sodium phosphate heptahydrate, sodium sulfate, magnesium chloride hexahydrate, sodium tartrate dihydrate, sodium lactate, sodium pyruvate, 3-(4,5-dimethylthiazol-2-yl)-2,5-diphenyltetrazolium bromide (MTT), Alexa Fluor 488 NHS ester, transferrin Alexa Fluor 680 conjugate, dextran Alexa Fluor 680, cholera toxin subunit B Alexa Fluor 647 conjugate, formaldehyde, normal goat serum, Triton X-100, antifade mountant with DAPI, and Novex tris-glycine SDS running buffer were purchased from Thermo Fisher Scientific. 2-S-(4-aminobenzyl)-1,4,7-triazacyclononane-1,4,7-triacetic acid) (p-NH_2_-Bn-NOTA) was purchased from Macrocyclics. Dulbecco’s modified Eagle’s medium (DMEM), penicillin/streptomycin, Dulbecco’s phosphate-buffered saline (DPBS), and Hank’s balanced salt solution (HBSS) were purchased from Gibco. Laemmli sample buffer, protein standards, and mini-protein gel were purchased from Bio-Rad. ^64^Cu was purchased from the MIR Cyclotron Facility in Washington University School of Medicine in St. Louis.

### Synthesis of B-AuNSs:

B-AuNSs were synthesized by the one-step and seedless-mediated method.^[4,6,19]^ Briefly, 18 mL of Milli-Q water at 18 MΩ was mixed with 12 mL of 200 mm HEPES at pH 7.4 ± 0.2. Next, 300 μL of 20 mm HAuCl_4_·3H_2_O was added. The solution was mixed by gentle inversion and reacted at room temperature for 75 min.

### Functionalization of AuNSs:

To functionalize B-AuNSs with antibodies, bifunctional linkers OPSS-PEG-SVA-2000 were first reacted with IgG2a antibodies. Briefly, 72 μL of 1 mg mL^−1^ IgG2a antibody reconstituted in 100 mm NaHCO_3_ buffer (pH 8.4 ± 0.2) was mixed with 8 μL of 80 mg mL^−1^ OPSS-PEG-SVA-2000 solution and allowed to react on an inverter (4 rpm) at 4 °C for 24 h. Afterward, 80 μL of OPSS-PEG-anti-IgG2a was added to 6 mL of B-AuNSs at 1.14 mg mL^−1^. The solution was then mixed on an inverter at 4 °C for another 24 h. Post AuNSs-anti-IgG2a reaction, the chelator, NOTA, was conjugated to the gold. Briefly, OPSS-PEG-SVA-2000 linkers were reacted with NOTA at 1:1 molar ratio at 4 °C for 10 h. OPSS-PEG-NOTA was then reacted with AuNSs-anti-IgG2a at 4 °C for 12 h. Then the functionalized AuNSs (AuNSs-anti-IgG2a-NOTA) were centrifuged at 4000 rpm for 10 min and resuspended with Milli-Q water at a concentration of 5 mg mL^−1^. Lastly, AuNSs-anti-IgG2a-NOTA nanoparticles were radiolabeled with ^64^Cu followed by 75 min incubation at room temperature with gentle shaking every 15 min. PD-10 desalting columns were used to remove nonchelated copper.

### Characterization of Nanoparticles:

The plasmon resonance of B-AuNSs and F-AuNSs was measured with a Varian Cary 5000 UV–vis NIR spectrophotometer. The hydrodynamic size and zeta potential of nanoparticles were measured with a Malvern Nano ZS dynamic light scattering apparatus. The size and shape of AuNSs were visualized with an Osiris TEM at 200 keV.

### Cell Viability:

RAW 264.7 and J774A.1 cells were cultured in DMEM supplemented with 10% FBS and 1% penicillin/streptomycin. Cell cultures were maintained at 37 °C in a humidified 5% CO_2_ atmosphere. Cells were seeded at 1 × 10^4^ cells per well in a 96-well plate and treated with different concentrations of F-AuNSs for 24 h. Following incubation, old media in each well were removed, and 100 μL of fresh media mixed with 10 μL of 12 mm MTT was added to each well. After 2 h of incubation, 85 μL of media solution was removed and 50 μL of DMSO was added to solubilize the precipitated formazan crystals. The plates were incubated at 37 °C for 10 min, and the absorbance was measured at 540 nm using a BIO-TEK Synergy H1 plate reader.

### Cell Cycle Analysis:

Cells were seeded at 3 × 10^5^ cells per well in a 6-well plate and then treated with F-AuNSs (100 μg mL^−1^) for 24 h. After incubation, cells were harvested and centrifuged at 1200 rpm for 10 min, and the supernatant was discarded. The cell pellets were fixed with 66% ethanol and stored at −20 °C until analysis. The fixed cell suspensions were washed three times with PBS by centrifugation at 1500 rpm for 10 min. The cells were resuspended in 0.1% Triton X-100. The cell pellets were further treated with the mixture of propidium iodide (50 μg mL^−1^) and RNase A (550 U mL^−1^) at 37 °C in the dark for 30 min. Samples were then subjected to a flow cytometer (BD FACSCanto), and data were analyzed using BD FACSDiva (version 8.0.1).

### Degradation of F-AuNSs in Artificial Lysosomal Fluid:

ALF was prepared by mixing 10 mL of Milli-Q water at 18 MΩ with 64.2 mg of sodium chloride, 120 mg of sodium hydroxide, 416 mg of citric acid, 19.4 mg of calcium chloride, 3.58 mg of sodium phosphate heptahydrate, 0.78 mg of sodium sulfate, 2.12 mg of magnesium chloride hexahydrate, 1.18 mg of glycine, 1.54 mg of sodium citrate dihydrate, 1.8 mg of sodium tartrate dihydrate, 1.7 mg of sodium lactate, 1.72 mg of sodium pyruvate, and 1 μL of 4% formaldehyde solution as an antibacterial agent. The mixture was then pH corrected to a pH of 4.5 with 1 m sodium hydroxide. F-AuNSs and additional Milli-Q were added to the mixed solution to obtain a 0.5 mg mL^−1^ F-AuNSs and 50% diluted ALF solution. The F-AuNSs-ALF solution was placed on a thermomixer at 60 rpm and 37 °C. Solutions were taken for plasmon resonance measurements at the following time points: 0.5 h, 1 h, 3 h, 6 h, 18 h, 1 day, 2 days, 3 days, 5 days, and 14 days. The TEM images of F-AuNSs were performed at 7, 14, and 21 days.

### In Vivo Toxicity Study of F-AuNSs:

Female 10-week-old mouse (C57BL/6, Jackson laboratory) was injected IP or IV with 0.8 mg of F-AuNSs suspended in 100 μL PBS. Mice were sacrificed at 1-, 7-, 30-, 45- or 90-days postinjection. Cardiac blood (≈600 μL per mouse) was collected for both complete blood count and serum liver/kidneys metabolite studies. Complete blood counts were performed using the forcyte veterinary hematology analyzer (Oxford Science), and blood chemistries were measured by the Vet Axcel chemistry analyzer (Alfa Wassermann). Additionally, liver, spleen, kidneys, heart, and lungs of each mouse were retrieved and fixed in 6% formalin for H&E staining. H&E images were captured using Leica DMi8.

### PET Imaging and Biodistribution:

Mice were injected IP or IV with 0.8 mg of F-AuNSs suspended in 100 μL PBS that had 800 μCi of ^64^Cu activity. Afterward, mice were placed in a small animal imaging PET/CT machine (Siemens Inveon) and imaged at 2 and 24 h postinjection. The mice were anesthetized with 2% isoflurane during imaging. All PET data sets were reconstructed using the MAP algorithm into 128 × 128 × 95 slices with a voxel size of 0.095 × 0.095 × 0.08 cm^3^ at a beta value of 0.01. The PET images were normalized to the injected dose. After imaging (24 h postinjection), mice were euthanized by cervical dislocation under deep isoflurane anesthesia. Tissues were then harvested, weighted, and placed in scintillation vials for gamma counting using Hidex AMG automatic gamma counter.

### Inductively Coupled Plasma Mass Spectrometry:

Mice were injected IP or IV with 0.8 mg of F-AuNSs suspended in 100 μL PBS. For each mouse, the liver, spleen, kidneys, heart, lungs, stomach, brain, muscle, bone, pancreas, and intestines were retrieved at 1-, 7-, 30-, 45- or 90-days postinjection. Tissues were first snap frozen in liquid nitrogen. Tissues were then dried with a lyophilizer (Labconco), weighted, and then placed in 80% trace-metal grade aqua regia for 72 h. Afterward, aqua regia was boiled off and the tissue samples were reconstituted with 10 mL of 2% nitric acid. Filters with 0.45 μm diameter were used to remove any impurities in the samples prior to ICP-MS measurements. The ICP-MS instrument (Perkin Elmer NexION 2000) was operated at 1.5 kW radio frequency power, 15 L min^−1^ argon plasma flow, 0.9 L min^−1^ nebulizer flow, and 1 s integration time for 3 replicates. A six-point calibration curve in the range of 0.5 and 1000 μg L^−1^ was performed for gold. Analytical blanks and standards (10 μg L^−1^) were measured for every 3–5 samples to ensure the readings were within 15% of the specified value. Blood, urine, and feces at 1-, 7-, 30-, and 45-days postinjection were digested for 72 h in 80% trace-metal grade aqua regia, filtered, diluted to 4% acid, and analyzed via ICP-MS (Tofwerk icpTOF-S2) equipped with an autosampler (Elemental Scientific, microFAST MC). Iridium (5 μg L^−1^) was used as the internal standard to monitor instrument response and correct for drift. Samples were introduced into the ICP at a liquid flow rate of 110 μL min^−1^ and the ICP was operated at a power of 1.5 kW with 15 L min^−1^ argon cooling gas flow, 1 L min^−1^ argon auxiliary flow, and 1 L min^−1^ nebulizer flow. Gold was quantified using a six-point calibration curve from standard solutions with concentrations from 0.1 to 100 μg L^−1^; and five replicates of each sample were analyzed (30 s spectral collection period per replicate). Standards (5 or 10 μg L^−1^) were run every five samples to ensure values were within 15%.

### Transmission Electron Microscope Imaging of Tissues:

Mice were injected IP or IV with 0.8 mg of F-AuNSs suspended in 100 μL PBS . The mice were sacrificed at 7-, 45- or 90-days postinjection. Liver, spleen, and kidneys were then retrieved. All tissues were dissected into 1 mm by 1 mm pieces with razor blades and immediately immersed in 2.5% glutaraldehyde in 0.1 m cacodylate buffer (pH 7.4 ± 0.1) for 24 h at 4 °C. The specimens were further fixed with 1% osmium tetroxide for 1 h and enblock stained with 1% uranyl acetate for 30 min. The samples were dehydrated with a graded ethanol series and then infiltrated with Epon 812 resin (Electron Microscopy Sciences) using propylene oxide as a transition solvent. The Epon 812 was polymerized at 60 °C for 48 h, sectioned at 70 nm using a Leica EM UC7 Ultramicrotome and collected on 300 mesh nickel grids. The sections were stained with 2% uranyl acetate and lead citrate. TEM imaging was performed on a Tecnai T12 at 100 kV using an AMT CCD camera. The surface area of >100 single particles in TEM images at each time point was measured with ImageJ. The average surface area of those particles at each time point was compared that to as-prepared F-AuNSs before injection.

### Nanoparticle Uptake Studies:

To investigate the mechanism of nanoparticle uptake, the synthesis of particles was slightly modified. Post AuNSs-anti-IgG2a reaction, particles were fluorescent labeled instead of conjugation with NOTA. For fluorescent labeling, 25 mg mL^−1^ of NH_2_-PEG-SH-5000 was first mixed with 1 mg mL^−1^ of Alexa Fluor 488 NHS ester at room temperature for 2 h on an inverter. The mixture was then reacted with AuNSs-anti-IgG2a at 4 °C for 3 h. The functionalized AuNSs (AuNSs-anti-IgG2a-488) was centrifuged at 4000 rpm for 10 min to remove excess free dyes and resuspended with Milli-Q water at a concentration of 5 mg mL^−1^.

For the uptake studies, cells were seeded at 3 × 10^5^ cells per well in a 6-well plate overnight. Prior to the exposure to nanoparticles, cells were first pretreated at 37 °C with different pathway inhibitors: cytochalasin B (10 μg mL^−1^, 2 h), genistein (200 μm, 1 h), rottlerin (2 μm, 30 min), and monodansyl cadaverine (200 μm, 10 min), respectively. To investigate the energy-dependence of uptake mechanism, cells were preincubated at 4 °C for 1 h. Afterward, F-AuNSs (100 μg mL^−1^) were added and incubated for 6 h. Negative controls (i.e., cells without the presence of inhibitors and nanoparticles) were also included. The mechanism of nanoparticle uptake was determined by flow cytometry.

### Fluorescence Imaging:

J774A.1 cells (1000 cells per well) were seeded in 8-well chamber slides (Lab-Tek) and incubated at 37 °C and 5% CO_2_ overnight. Subsequently, media were removed, and fluorescently labeled F-AuNSs were coincubated with different endocytic markers: transferrin Alexa Fluor 680 (0.05 mg mL^−1^), dextran Alexa Fluor 680 (0.5 mg mL^−1^), and cholera toxin subunit B Alexa Fluor 647 (0.002 mg mL^−1^). After 4 h, incubation media were aspirated and washed twice with 1× DPBS and then supplemented with 0.5 mL of HBSS. Fluorescence microscope (ECHO, Revolve 4) with a 20× objective was employed to visualize the fluorescence images from samples.

### Confocal Imaging:

Cells (5000 cells per well) were seeded in 8-well chamber slides (Lab-Tek) and incubated overnight. Cells were then exposed to F-AuNSs (100 μg per mL) at different time points (2, 4, 6, 8, 12, 18, and 24 h), fixed with 4% formaldehyde solution for 10 min, permeabilized with 0.1% Triton X-100 for 1 h, and blocked with 10% normal goat serum solution at room temperature for 1 h. Cells were incubated overnight at 4 °C with anti-EEA1 antibody (Abcam, ab109110, 1/250) for early endosome staining or anti-RAB7 antibody (Abcam, ab126712, 1/50) for late endosome staining, and washed three times followed by staining with Alexa Fluor 594-conjugated anti-rabbit IgG (Abcam, ab150080, 1/500). For lysosome imaging, cells were stained with BioTracker NIR 633 (Sigma, 1/500) according to the manufacturer’s instructions. Slides were mounted with DAPI nuclear dye and visualized under a Leica SP5 X MP confocal microscope. Images were captured utilizing a 63× objective.

### ImageJ Analysis:

ImageJ was used to measure the total surface area of particles, i.e., clusters of gold nanostars (represented as black dots in cell images) colocalized with early endosome, late endosome, and lysosome, respectively, in 50 cells. The normalized surface area is represented in Figure 5.

### Proteins Immobilized on Nanoparticles:

To study the protein corona on B-AuNSs and F-AuNSs, the nanoparticle dispersion was incubated with 60% FBS at 37 °C for 1, 24, and 48 h under constant agitation or 60% mouse serum obtained from C57BL/6 mice at 37 °C for 24 h. Particles were separated from the supernatant by centrifugation at 6000 rpm and 4 °C for 20 min. The pellets were resuspended in PBS and washed three times by centrifugation at 4000 rpm and 4 °C for 10 min to remove unbound proteins.

### SDS-PAGE Electrophoresis:

Proteins immobilized on particles (5 mg mL^−1^) was mixed with Laemmli sample buffer (2×) containing freshly added 5% *β*-mercaptoethanol at a ratio of 1:1 and boiled at 95 °C for 10 min. Treated samples were then loaded in a 4–20% mini-protein gel. The gels were run for 30 min at 200 V in Novex tris-glycine SDS running buffer (1×). Silver staining of gels was performed according to the manufacturer-provided procedures (Fujifilm Wako, 291–50301). Gels were visualized by UMAX PowerLook 1100 scanner. Proteins adsorbed onto particles were quantified using ImageJ.

### Proteomic Studies of Surface Protein Corona:

Crude protein extracts were reduced with DTT. The cysteines were modified with iodoacetamide and then digested overnight with trypsin/Lys-C at an enzyme to protein ratio of 1:25. Formic acid was added to stop digestion. Samples were then desalted using a C18 MicroSpin columns (Nest Group) before drying in a speedvac concentrator. The peptides were then separated using an EASY-nLC 1200 UHPLC system coupled to Nanospray Flex ion source (Thermo Fisher Scientific). The Q Exactive Hybrid QuadrupoleOrbitrap mass spectrometer with an HCD fragmentation cell (Thermo Fisher Scientific) was used for mass spectrometric analysis. Raw files were analyzed using Proteome Discoverer (Thermo Fisher Scientific, version 2.4). MS/MS spectra were searched with Mascot against Sprot-all.

### Martini Coarse-Grained Simulation:

All simulations were performed using the Martini CG force field (version 2.2)^[86]^ with the GROMACS 5.1.4 package.^[87]^ The Martini model is based on a four-to-one mapping indicating that on average four heavy atoms and associated hydrogens are represented by one CG bead.^[86]^ Nonbonded interactions between neutral beads of Martini are described by Lennard-Jones potentials, while charged beads also include Coulombic interactions.^[71]^ The welldefined interaction strength of the Lennard-Jones potential allows for discrimination between subtly different levels of polarity of the CG beads. Both van der Waals and electrostatic interactions had a cutoff of 1.1 nm. The isotropic Parrinello-Rahman pressure coupling scheme was coupled to control the pressures at 1 bar and the compressibility at 3 × 10^−4^ bar^−1^.^[88]^ The system temperature was maintained at 310 K by v-rescale coupling scheme with a time constant of *τ* = 1 ps.^[89]^ Periodic boundary conditions were imposed in all three *x*-, *y*-, and *z*-directions. The AuNP was placed approximately at the center of the vesicle. Initially, the AuNP was fixed to perform a 100 ns balance simulation. To gain the insights into the complete transcellular transport process of AuNP from the membrane, pull simulations were applied in the *z* direction by using a constant force 1000 kJ mol^−1^ nm^−2^ to the center of mass of the AuNP. All systems were simulated for 300 ns with a time step of 20 fs. Data visualization was performed with visual molecular dynamics.^[90]^ Four different shapes of AuNPs were examined including sphere, cylinder, cone and tetrahedron. Two different sizes of AuNPs were studied including 4.5 and 35 nm^3^. The nanocrystalline structure of AuNPs was characterized by the face-centered cubic structure with a lattice constant of 0.408 nm. The aggregated AuNPs consist of five sphere AuNPs with a diameter of 2 nm each, and the pairs are connected together by covalent bonds resulting in an overall 4 nm in diameter. For the membrane vesicles, the initial structure was constructed online by CHARMM-GUI Martini Maker.^[91]^ To evaluate the effect of membrane composition on the AuNP translocation, three different types of vesicles with an initial diameter of 17 nm were constructed: purely composed of DPPC, purely composed of DOPC, and DPPC mixed with DPPG at a 3:1 ratio. The DPPC lipid membrane with fully saturated chains contained a total of 2550 lipid molecules. The DOPC lipid membrane was unsaturated with one double bond in each tail containing a total of 2394 lipid molecules. The membrane composed of both DPPC and DPPG had a total of 2564 lipid molecules. The initial size of the simulation box was 26 × 26 × 80 nm^3^. Water molecules and counterions (Na^+^ and Cl^−^) were added in the simulation box to neutralize the system.

### Statistical Analysis:

All data were presented as mean ± standard deviation. The sample sizes were estimated based on our previously published work. Statistical significance was determined by GraphPad Prism 8 with unpaired two-sided Student’s *t*-tests for the calculation of *p* values. Here, * indicates *p* < 0.05, ** indicates *p* < 0.01, *** indicates *p* < 0.001, and **** indicates *p* < 0.0001.

## Supplementary Material

Supplementary Information

## Data Availability

The data that support the findings of this study are available from the corresponding author upon reasonable request.
