# Supplementary material for: Physicochemical Properties and Route of Systemic Delivery Control the In Vivo Dynamics and Breakdown of Radiolabeled Gold Nanostars
Source: Small. Author manuscript; Available in PMC 2023 Sep 28. (PMC10518372; doi:10.1002/smll.202204293)
Supplement: Supplementary Information [file NIHMS1891693-supplement-Supplementary_Information.docx]

**SUPPORTING INFORMATION**

**Physicochemical Properties and Route of Systemic Delivery Control the *in vivo* Dynamics and Breakdown of Radiolabeled Gold Nanostars**

Xiaona Wen^†,#^, Luping Ou^‡^, Gabriel Cutshaw^#,⊥^, Saji Uthaman^#,⊥^, Yu-Chuan Ou^†^, Tian Zhu^†^, Sarah Szakas^||^, Brandon Carney^§^, Jacob Houghton^§^, Alexander Gundlach-Graham^||^, Marjan Rafat^†^, Kai Yang^‡^, and Rizia Bardhan^#,^^⊥,^*

^†^Department of Chemical and Biomolecular Engineering, Vanderbilt University, Nashville, Tennessee 37235, USA.

^#^Nanovaccine Institute, Iowa State University, Ames, IA 50012, USA.

^‡^Center for Soft Condensed Matter Physics and Interdisciplinary Research & School of Physical Science and Technology, Soochow University, Suzhou 215006, China.

^||^Department of Chemistry, Iowa State University, Ames, IA 50011, USA.

^§^Department of Radiology, Stony Brook University, Stony Brook, New York 11794, USA.

^⊥^Department of Chemical and Biological Engineering, Iowa State University, Ames, IA 50012, USA.

Corresponding Author

*****Email: [rbardhan@iastate.edu](mailto:rbardhan@iastate.edu)

**Table of Contents**

**p.S3 Figure S1.** Stability and shelf-life of F-AuNSs.

**p.S4 Figure S2.** Toxicity evaluation of F-AuNSs *in vitro*.

**p.S5 Figure S3.** Toxicity evaluation of F-AuNSs *in vivo*.

**p.S6 Figure S4.** H&E stain of major organs post IP delivery.

**p.S7 Figure S5.** H&E stain of major organs post IV delivery.

**p.S8 Figure S6.** Biodistribution of F-AuNSs *in vivo* using ^64^Cu gamma count.

**p.S9 Figure S7.** Biodistribution of F-AuNSs in major organs *in vivo* using ICP-MS.

**p.S10 Figure S8.** Biodistribution of F-AuNSs in blood, urine, and feces *in vivo* using ICP-MS.

**p.S11 Figure S9.** Degradation of F-AuNSs in artificial lysosomal fluid.

**p.S12 Figure S10.** TEM micrographs of F-AuNSs in artificial lysosomal fluid.

**p.S13 Figure S11.** Quantification of surface area of F-AuNSs from TEM images.

**p.S14 Figure S12.** TEM micrographs of liver after IP and IV injection of F-AuNSs.

**p.S15 Figure S13.** TEM micrographs of kidney after IP and IV injection of F-AuNSs.

**p.S16 Figure S14.** Diffraction pattern of TEM image.

**p.S17 Figure S15.** MTT cell viability assay and endocytosis of F-AuNSs in RAW 264.7.

**p.S18 Figure S16.** Fluorescence microscopy images of colocalization of F-AuNSs in J774A.1 incubated with different endocytic markers.

**p.S19 Figure S17.** Brightfield images of F-AuNSs incubated with early endosome, late endosome and lysosome in J774A.1.

**p.S20 Figure S18.** Confocal images of F-AuNSs colocalized with early endosome, late endosome and lysosome in RAW 264.7.

**p.S21 Figure S19.** Total surface area of F-AuNSs normalized and colocalized with early endosome, late endosome and lysosome in RAW 264.7.

**p.S22 Figure S20.** Proteomic study of surface protein corona formation on F-AuNSs upon incubation with 60% FBS.

**p.S23 Figure S21.** Proteomic study of surface protein corona formation on F-AuNSs upon incubation with mouse serum.

**p.S24 Table S1.** Proteins adsorbed on B-AuNSs and F-AuNSs.

**p.S25 Figure S22.** Snapshots of small- and large-sized NPs with four different shapes.

**p.S26 Figure S23.** Snapshots of the translocation of small-sized NPs with four different shapes across the DPPC lipid membrane as a function of time.

**p.S27 Figure S24.** The number of contacts between the small-sized NPs with four different shapes and DPPC lipid membrane as a function of time.

**p.S28 Figure S25.** Chemical structures of DPPC, DOPC and DPPG lipids.

**p.S29 Figure S26.** Effect of lipid composition on transcytosis.

**p.S29 Figure S27.** Snapshots of aggregated AuNPs with neutral and anionic surface charges.

**p.S30 Figure S28.** Snapshots of NPs for the evaluation of effect of aggregation on transcytosis.

**p.S31 Figure S29.** Interaction forces of NPs for the evaluation of effect of aggregation on transcytosis.


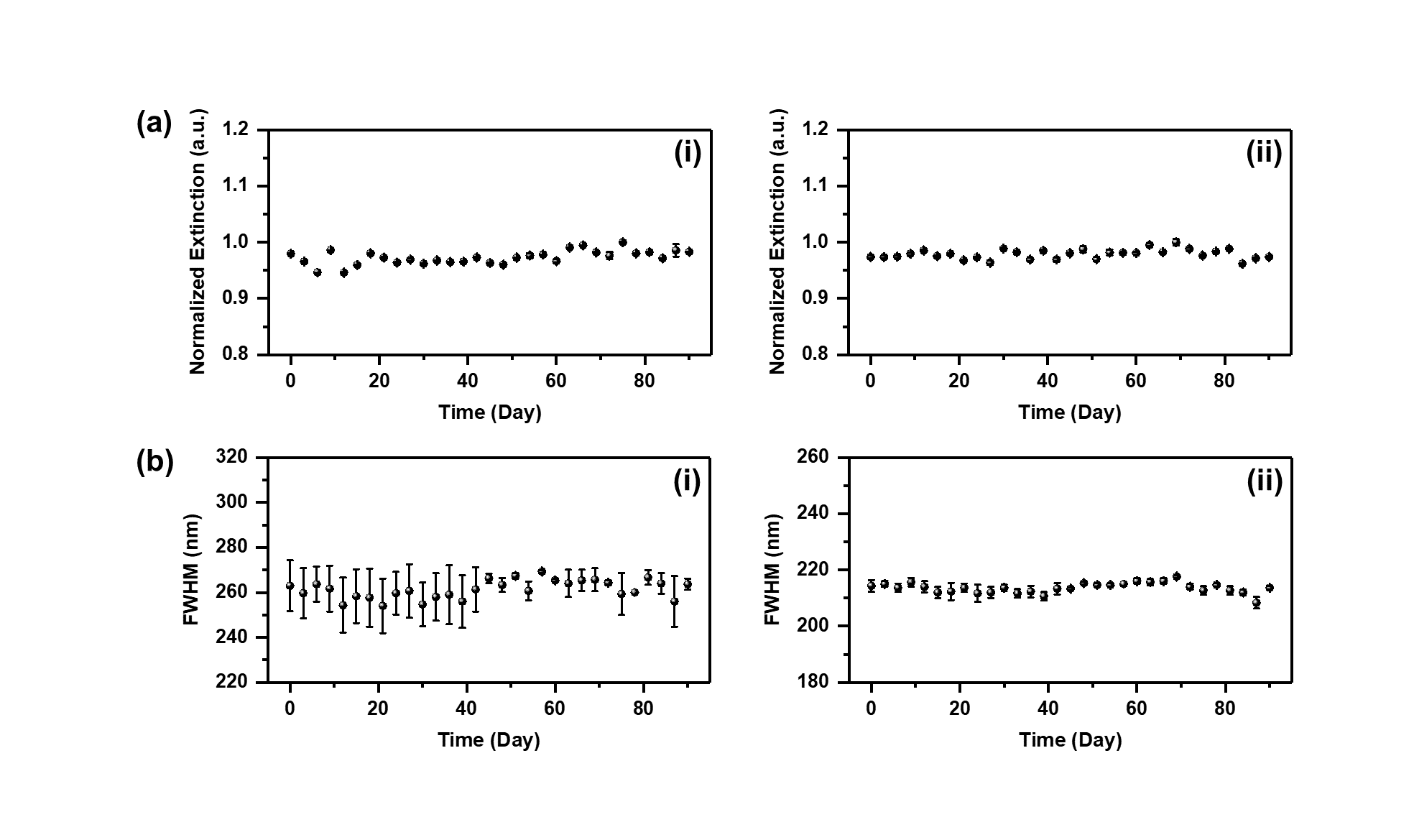


**Figure S1.** Stability and shelf-life of F-AuNSs. Aliquots of concentrated F-AuNSs were dispersed in (i) PBS and (ii) media supplemented with serum. (a) Normalized extinction and (b) FWHM were measured every three days over 90 days. F-AuNSs were stored at 4 °C between measurements. All data were represented as mean ± standard deviation (n = 3). As observed, the successful functionalization with stable ligands resulted in high stability of F-AuNSs for three months.

**
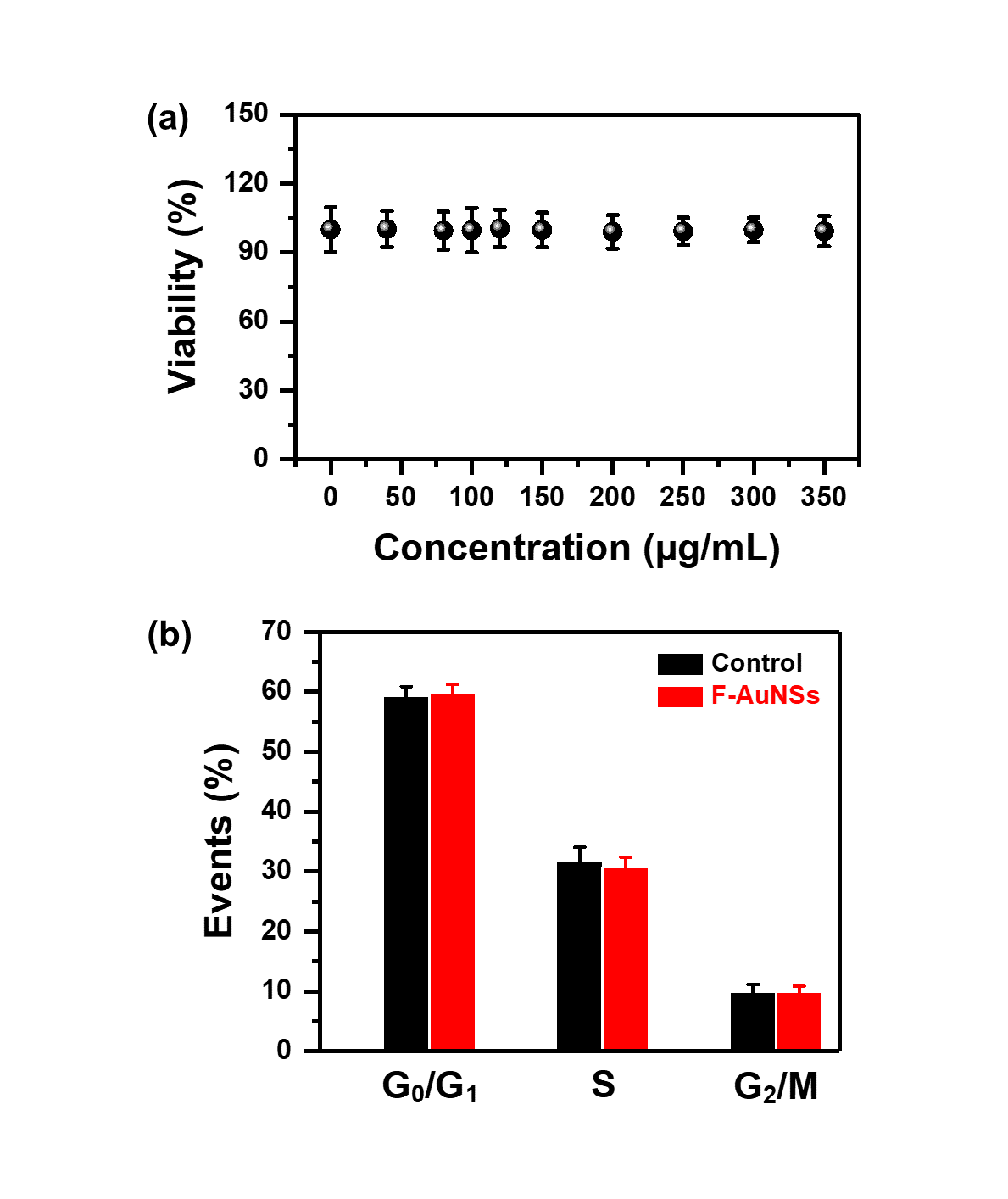
**

**Figure S2.** Toxicity evaluation of F-AuNSs *in vitro*. (a) MTT cell viability assay of RAW 264.7 cells incubated for 24 h with F-AuNSs at 0 – 350 µg/mL concentrations (n = 5 per concentration and N = 3 independent experiments). Cell viability was measured at 540 nm. All data were represented as mean ± standard deviation. (b) Cell cycle analysis of RAW 264.7 cells upon incubation with F-AuNSs (100 µg/mL) for 24 h. No significant changes were observed in the different cell cycle phases compared to control cells that did not receive F-AuNSs in both macrophage cell lines. All data were represented as mean ± standard deviation (n = 6 per group and N = 3 independent experiments).

**
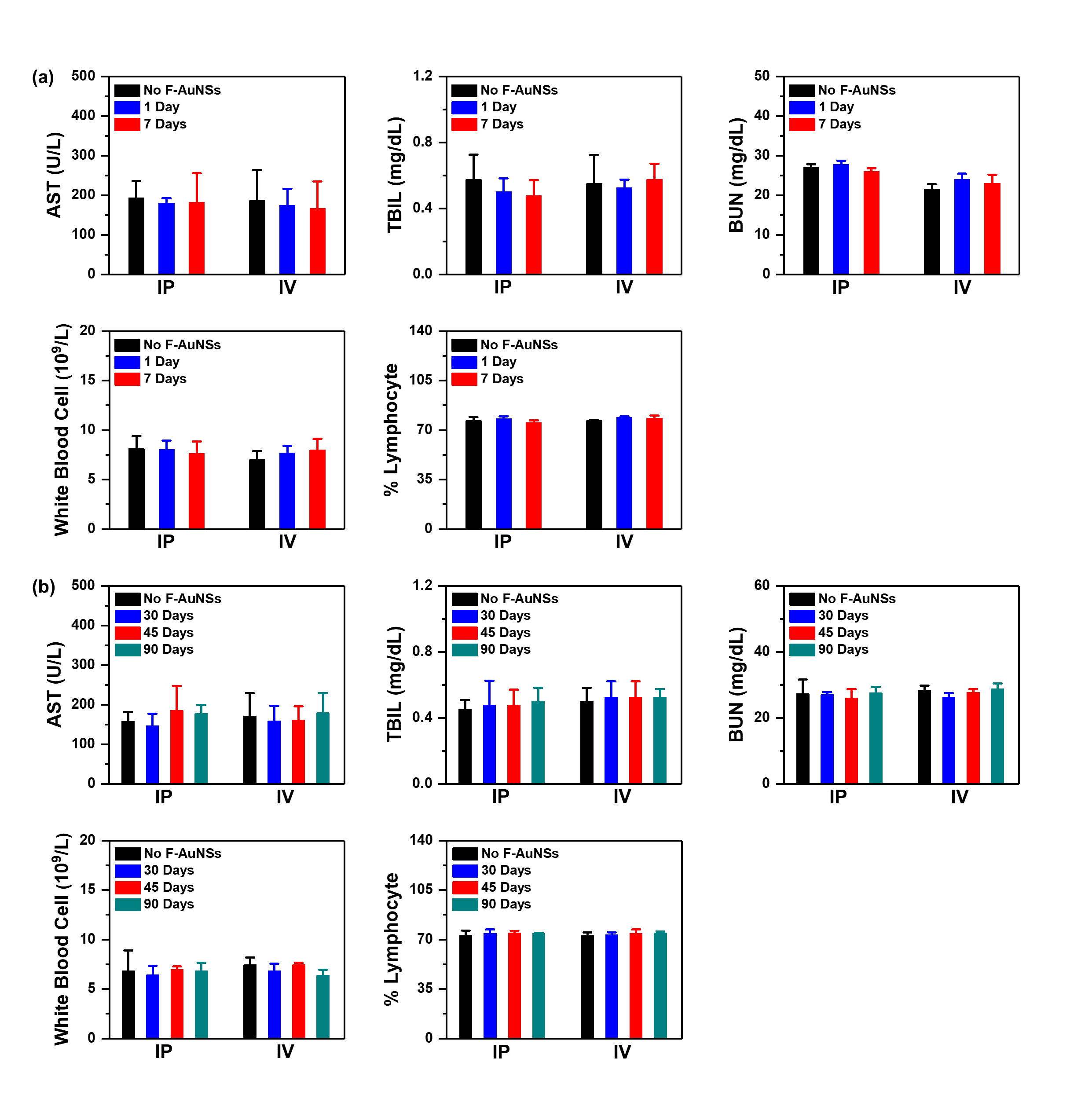
**

**Figure S3.** Toxicity evaluation of F-AuNSs *in vivo*. Serum inflammatory markers and complete blood count of mice that received F-AuNSs intraperitoneally (IP) and intravenously (IV) were compared to control mice which received PBS. (a) Short-term measurements included 1- and 7- days post-delivery. (b) Long-term measurements included 30-, 45- and 90-days post-injection. Irrespective of the route of delivery, no significant abnormalities based on the unpaired two-sided Student’s *t*-tests in hepatic, renal and hematological functions were observed, indicating F-AuNSs had high biocompatibility and minimal toxicity. All data were represented as mean ± standard deviation (n = 4).

**
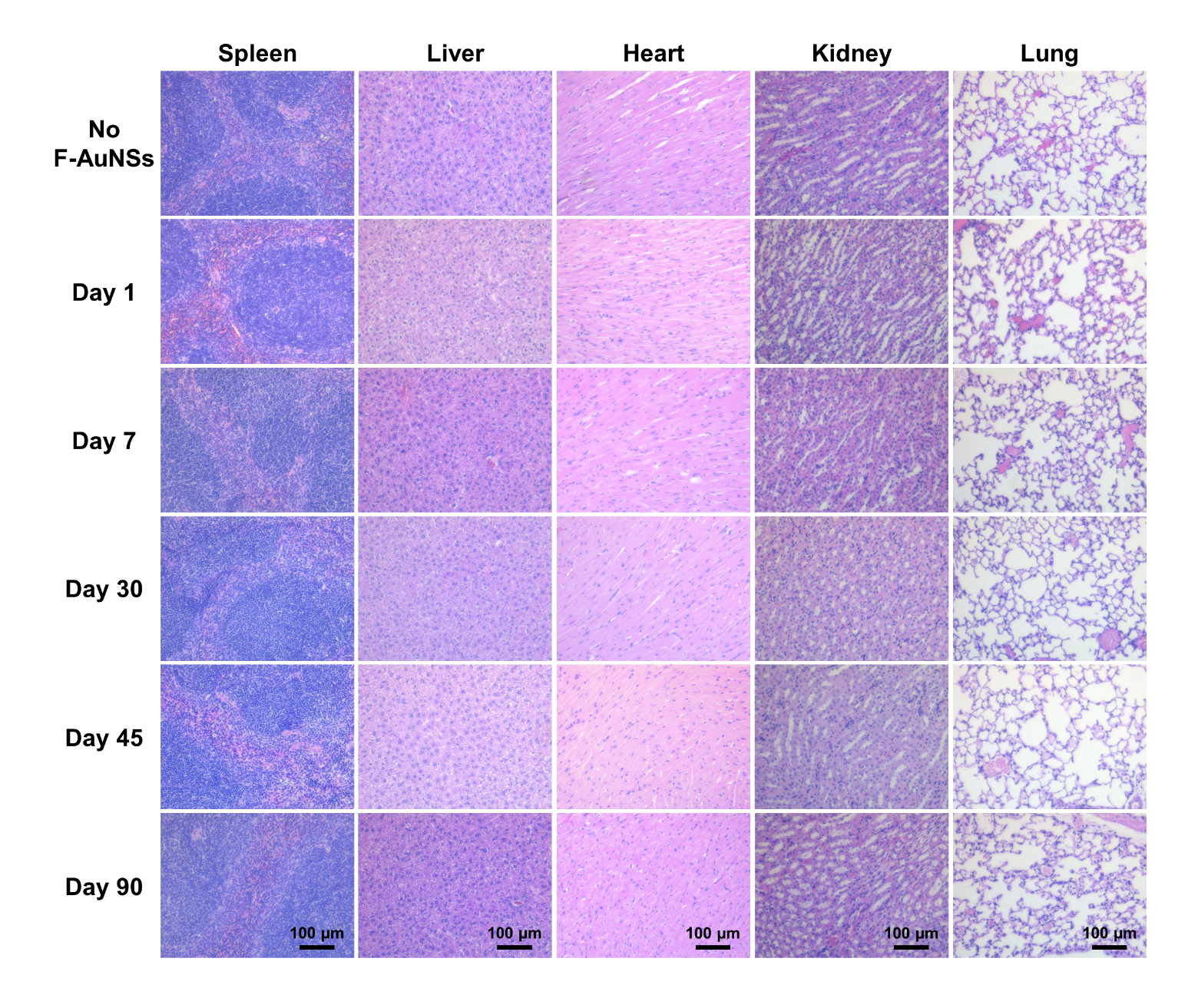
**

**Figure S4.** H&E stain of major organs post IP delivery of F-AuNSs in healthy C57BL/6 mice. Spleen, liver, heart, kidneys and lungs of mice were retrieved to evaluate the toxicity of F-AuNSs. Histological evaluation of tissues showed minimal toxicity by F-AuNSs up to 90 days post IP administration. Here, “No F-AuNSs” control group represents organs of mice that received PBS only. Histology was performed on n = 3 mice.


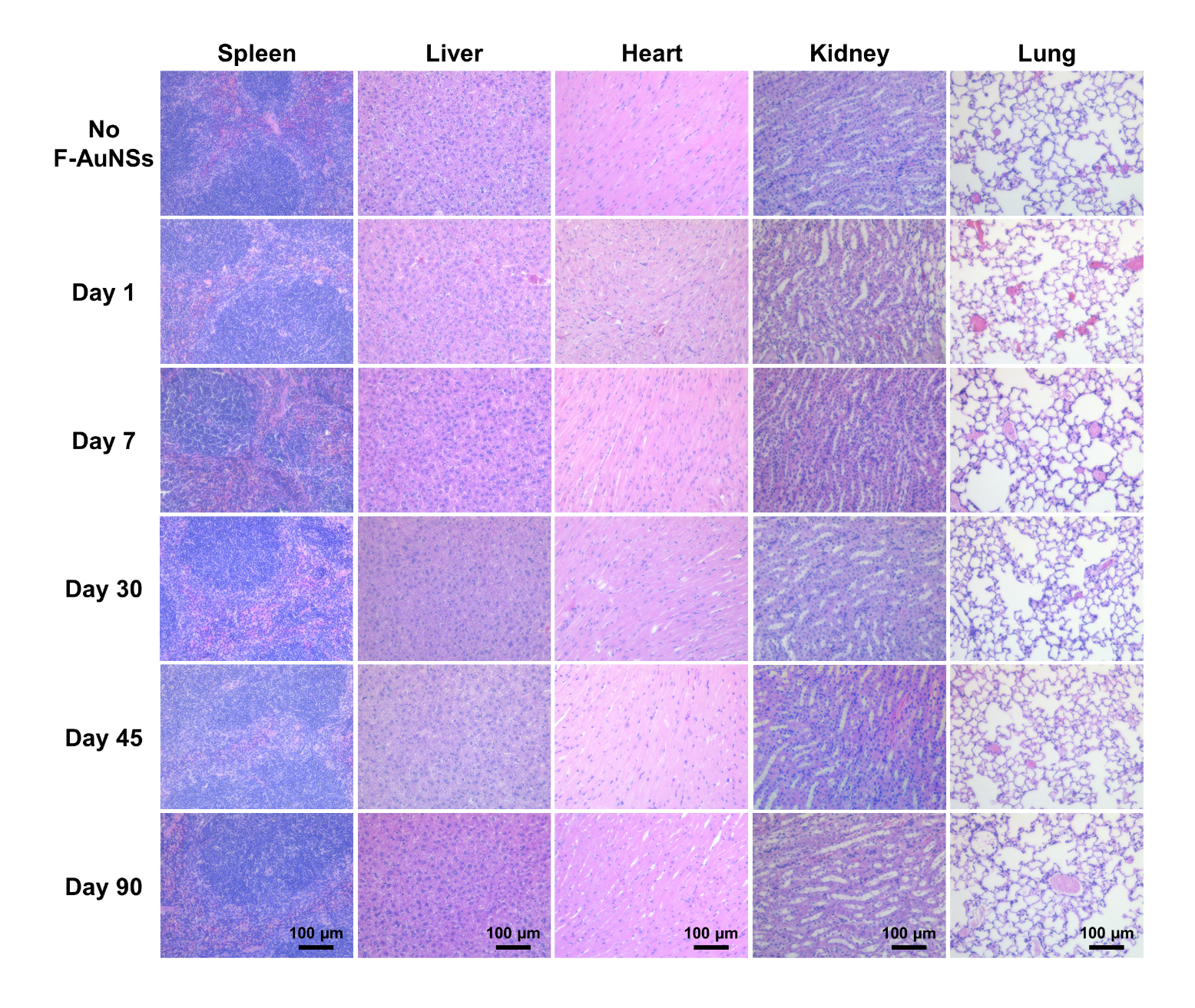


**Figure S5.** H&E stain of major organs post IV delivery of F-AuNSs in healthy C57BL/6 mice. Spleen, liver, heart, kidneys, and lungs of mice were retrieved to evaluate toxicity of F-AuNSs. Histological evaluation of tissues showed minimal toxicity by F-AuNSs up to 90 days post IV administration. Here, “No F-AuNSs” control group represents organs of mice that received PBS only. Histology was performed on n = 3 mice.


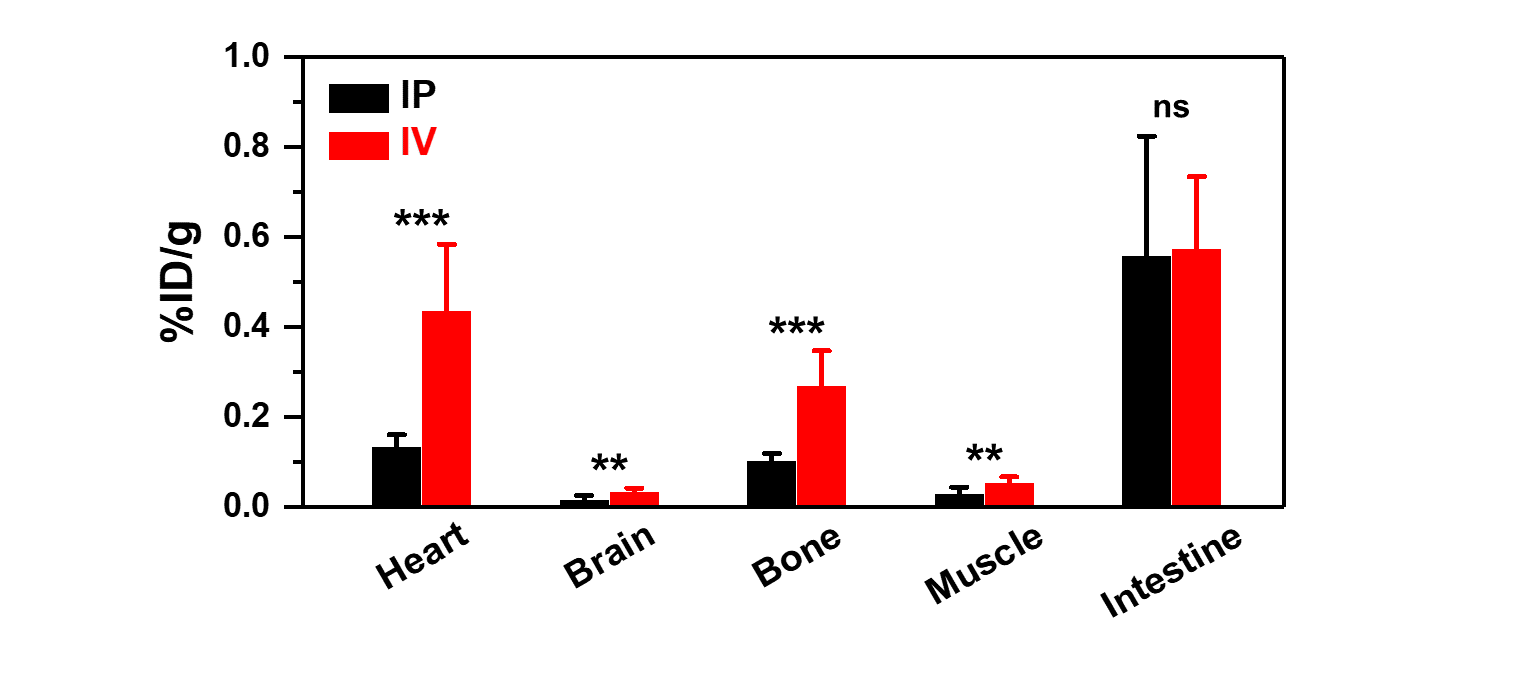


**Figure S6.** Biodistribution of F-AuNSs obtained with ^64^Cu gamma counts of organs harvested 24 h after either IP or IV delivery of F-AuNSs (n = 5). Here, ** indicates *p* < 0.01, *** indicates *p* < 0.001, and ns indicates not significant.


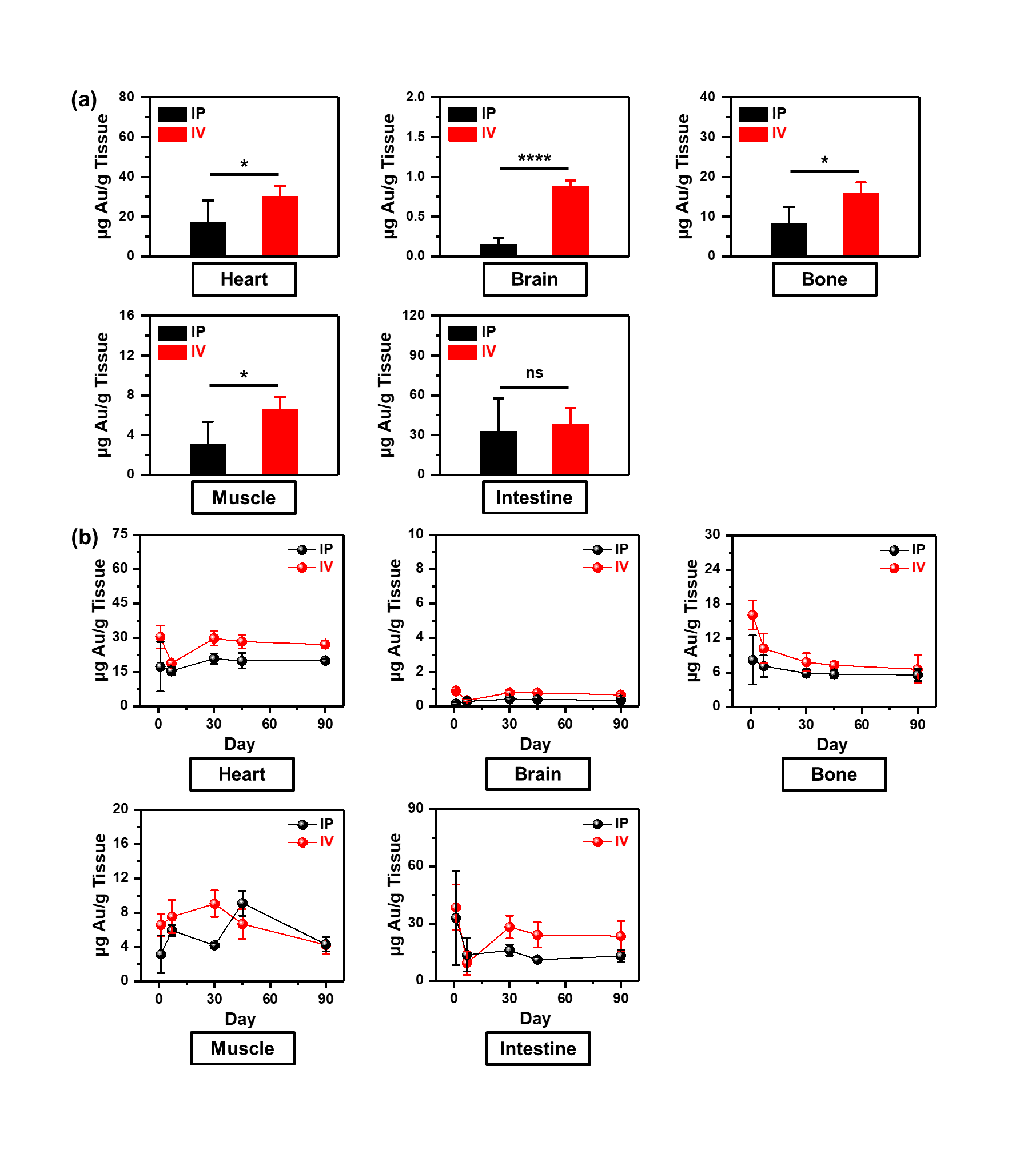


**Figure S7.** (a) Quantitative ICP-MS analysis of biodistribution of F-AuNSs in mice 24 h post particle delivery (n = 4) showing Au content in mice tissues. (b) Biodistribution and clearance of F-AuNSs of major organs at 1-, 7-, 30-, 45- and 90-days post-delivery (n = 4). Here, all data were represented as mean ± standard deviation. * indicates *p* < 0.05, **** indicates *p* < 0.0001, and ns indicates not significant.


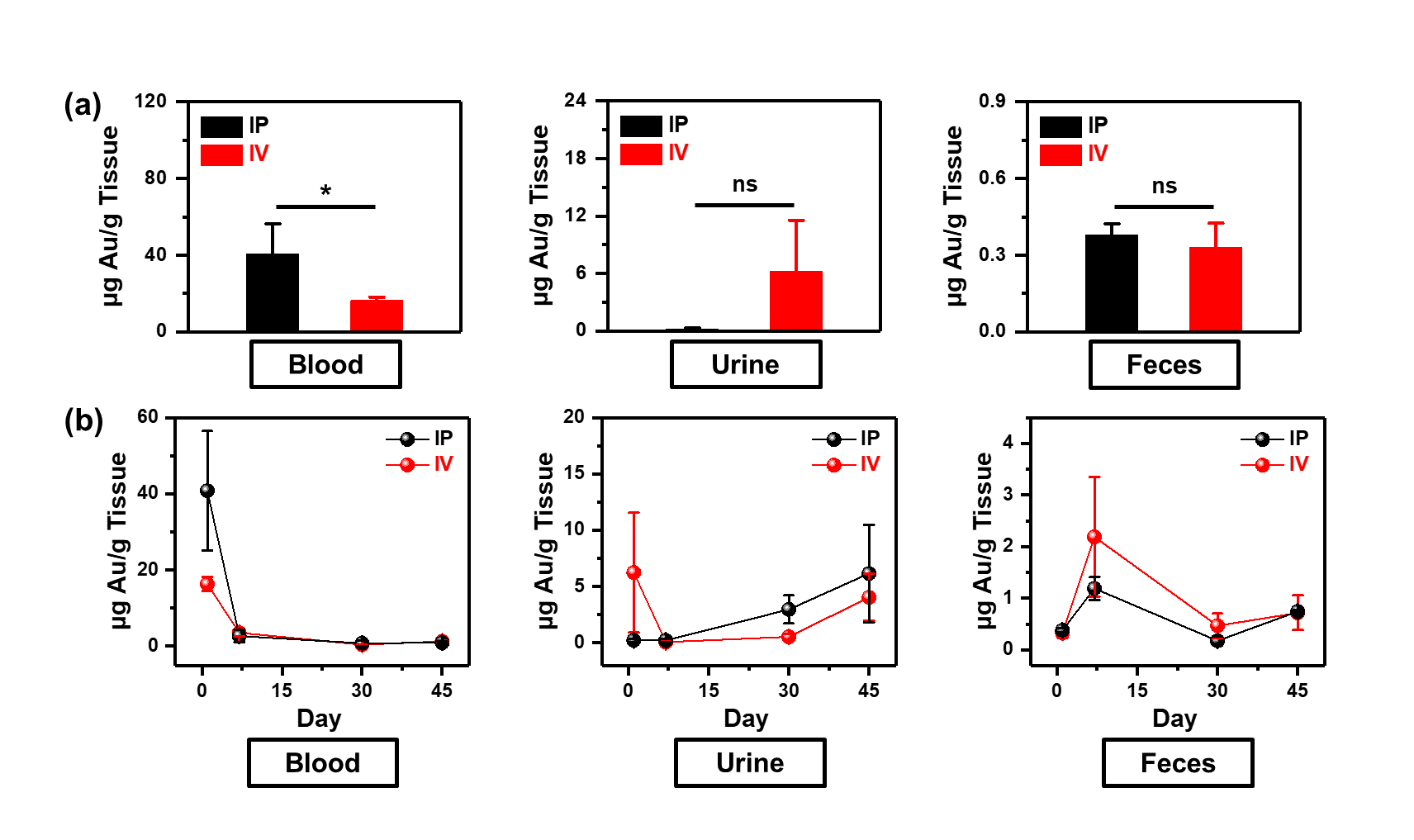


**Figure S8.** (a) Quantitative ICP-MS analysis of biodistribution of F-AuNSs in mice 24 h post–injection through both IP and IV delivery showing Au content in mice tissues (n = 3). (b) Biodistribution and clearance of F-AuNSs of major organs at 1-, 7-, 30- and 45-days post-delivery (n = 3). Here, all data were represented as mean ± standard deviation. * indicates *p* < 0.05 and ns indicates not significant. Our results demonstrated that F-AuNSs were most likely cleared through both hepatobiliary and urinary excretion.


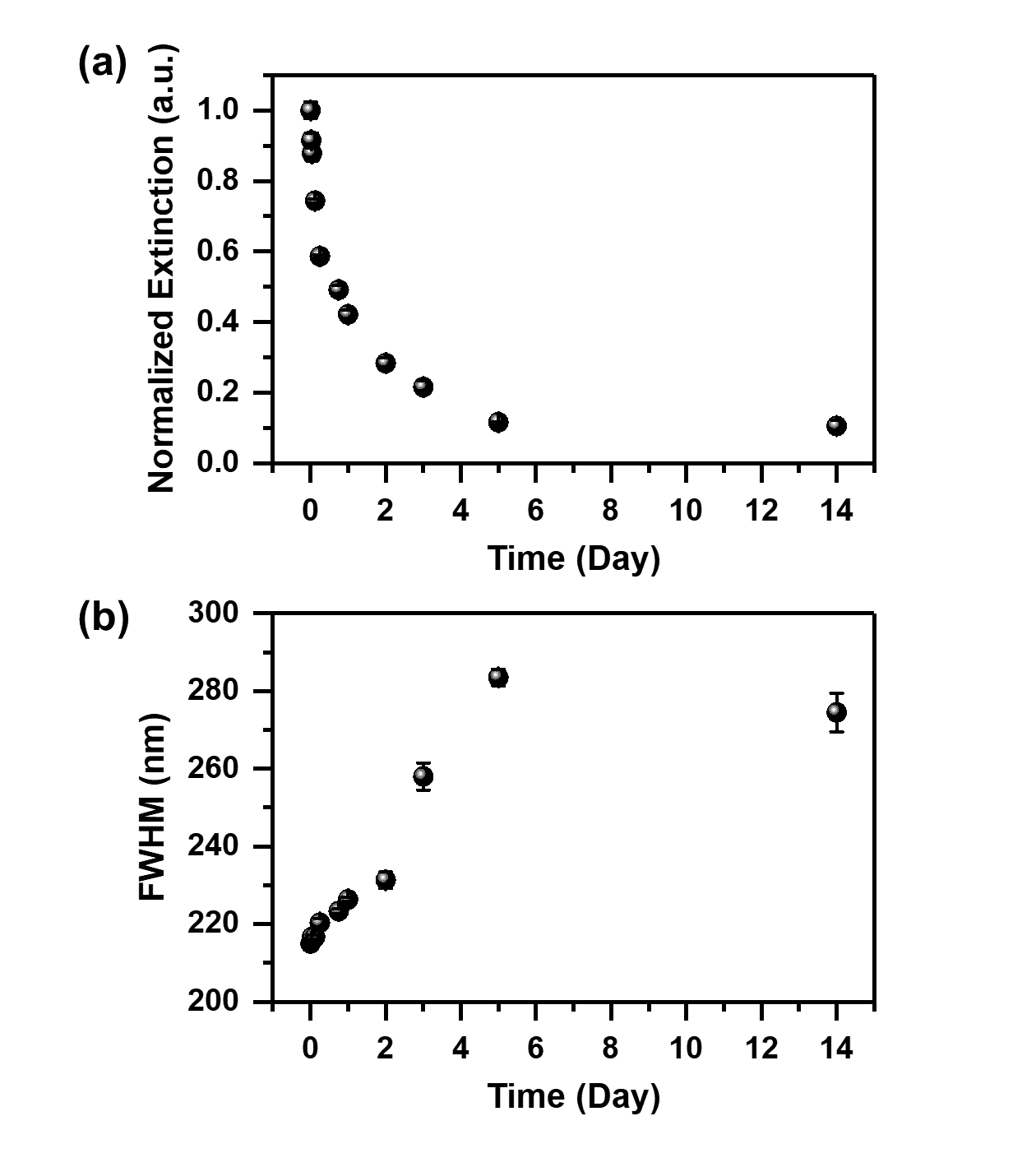


**Figure S9.** Stability and degradation of F-AuNSs in artificial lysosomal fluid. (a) Normalized extinction and FWHM were measured longitudinally. The F-AuNSs in ALF solution was placed on a thermomixer at 60 rpm and 37 °C and maintained at that temperature during the entire study. All data were represented as mean ± standard deviation (n = 3). The results indicated the degradation and/or aggregation of F-AuNSs in ALF.


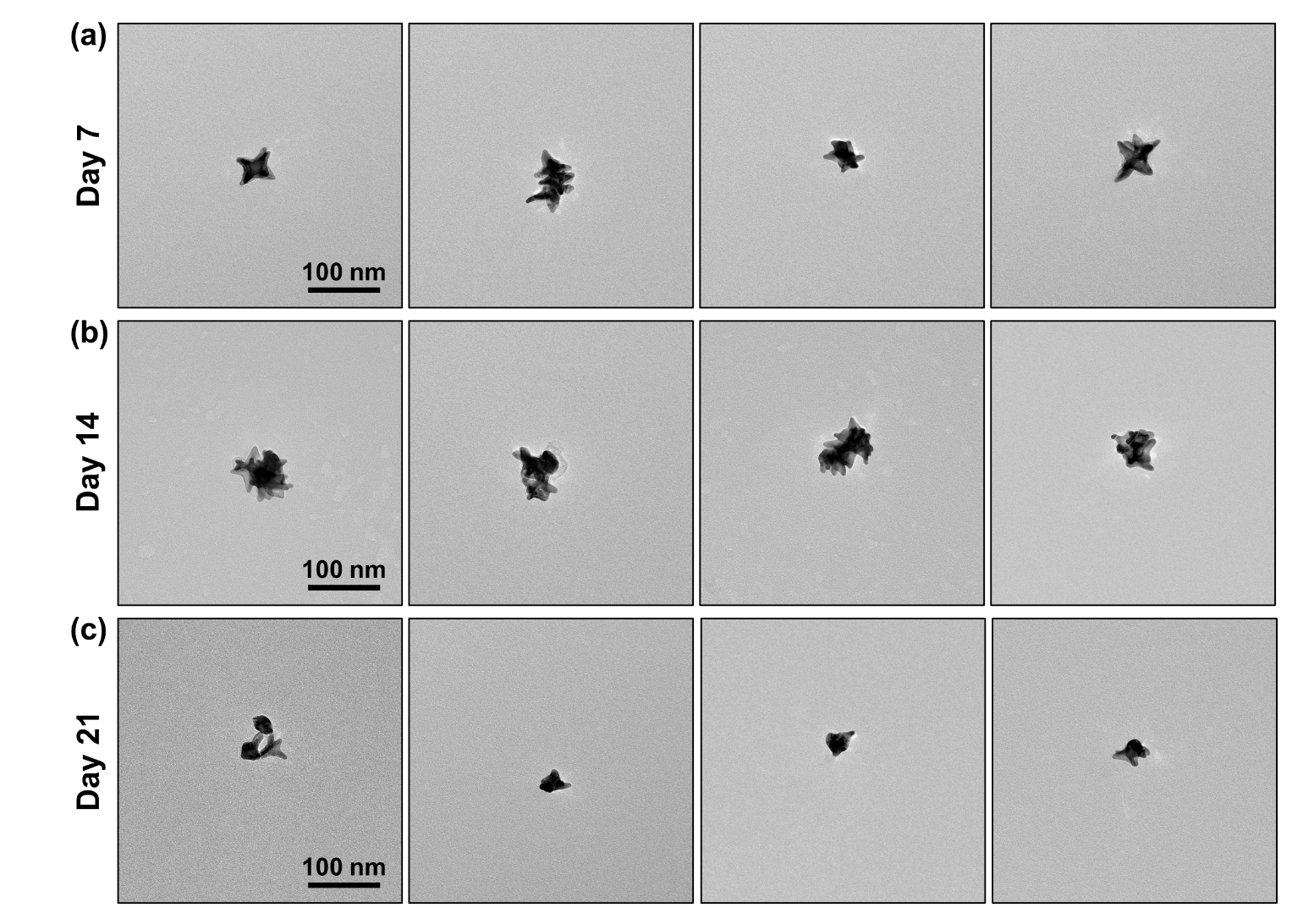


**Figure S10.** Representative TEM micrographs of F-AuNSs in artificial lysosomal fluid for (a) 7, (b) 14 and (c) 21 days.


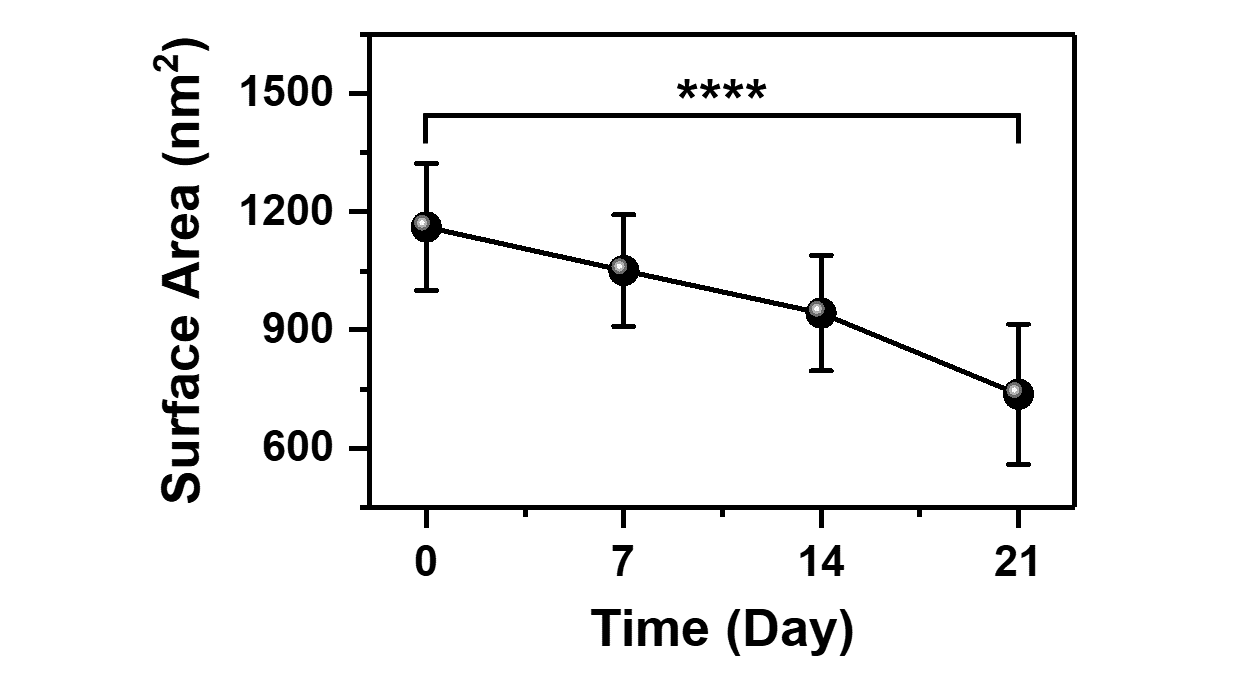


**Figure S11.** Quantification of surface area of F-AuNSs derived from TEM images where F-AuNSs were incubated in artificial lysosomal fluid (ALF) from 0 to 21 days. Day zero represents as-synthesized F-AuNSs before exposing to ALF. The error bars were calculated by counting the average surface area of 20 single particles per group from TEM images. Here, all data were represented as mean ± standard deviation. **** indicates *p* < 0.0001.


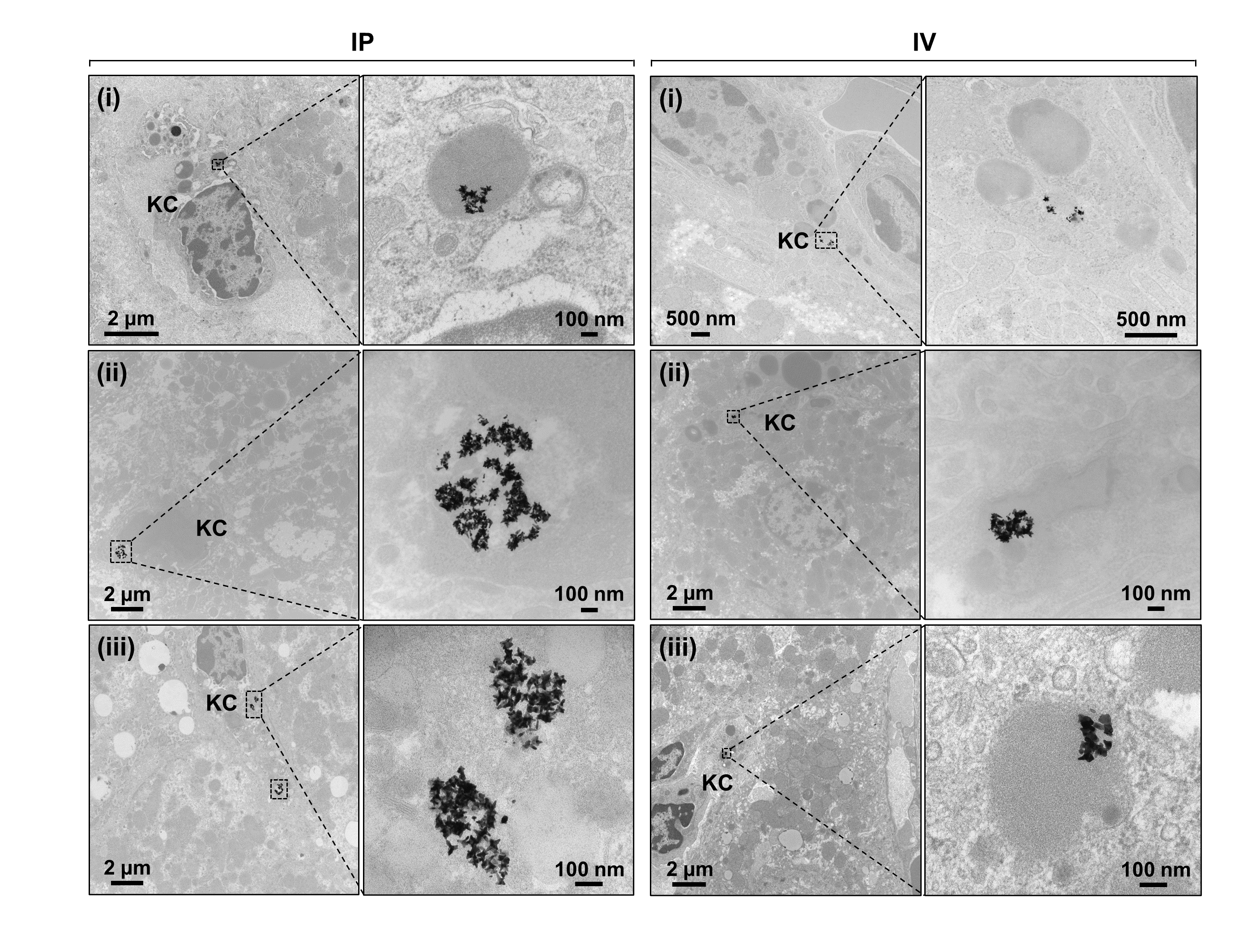


**Figure S12.** Representative TEM micrographs of liver harvested from mice (i) 7-, (ii) 45- and (iii) 90-days after IP delivery shown in left, and IV delivery shown in right of F-AuNSs. Here, *KC* represents Kupffer cells.


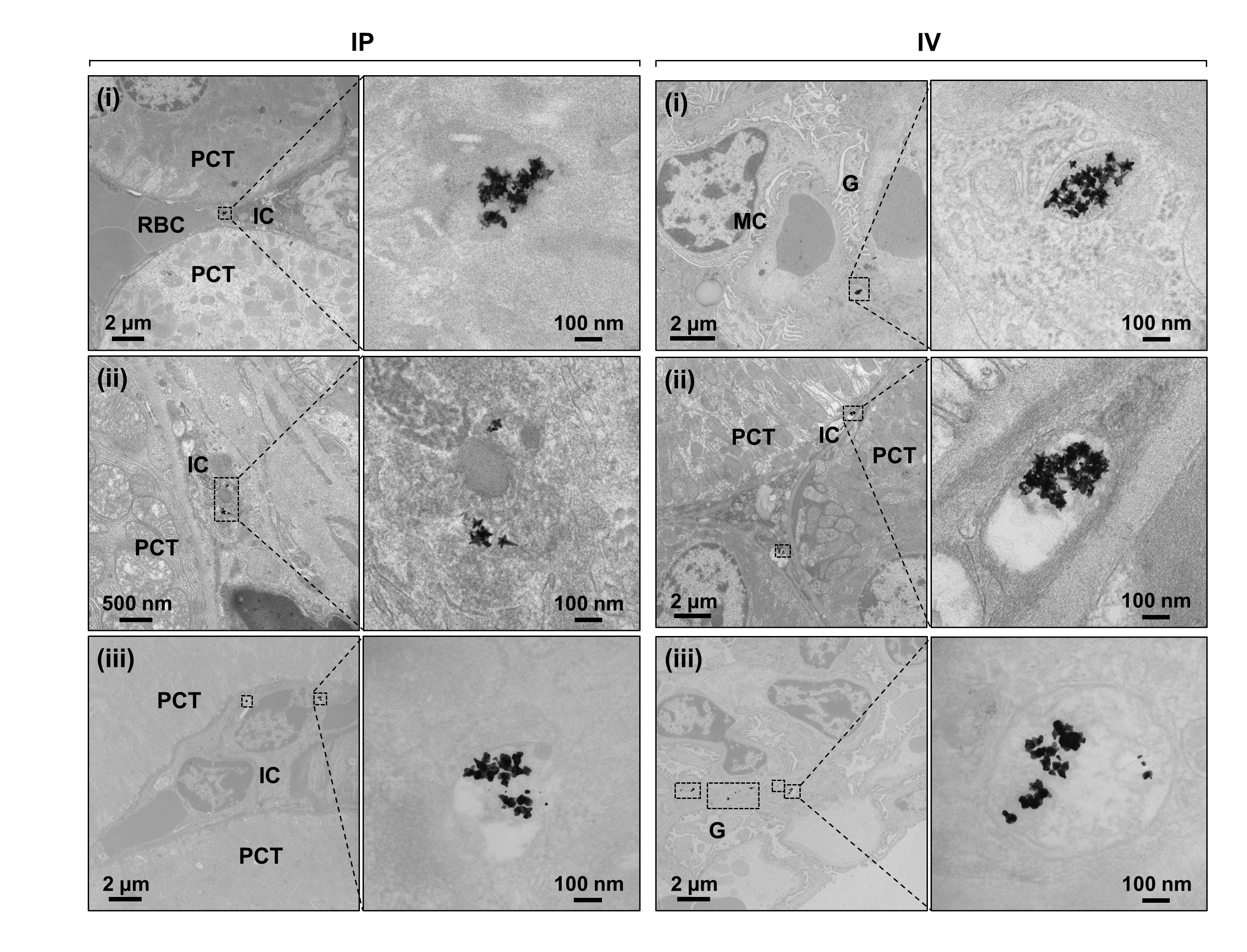


**Figure S13.** Representative TEM micrographs of kidney harvested from mice (i) 7-, (ii) 45- and (iii) 90-days after IP delivery shown in left, and IV delivery shown in right of F-AuNSs. Here, *G* represents glomeruli; *IC* represents interstitial cell; *MC* represents mesangial cell; *PCT* represents proximal convoluted tubule; and *RBC* represents red blood cell.


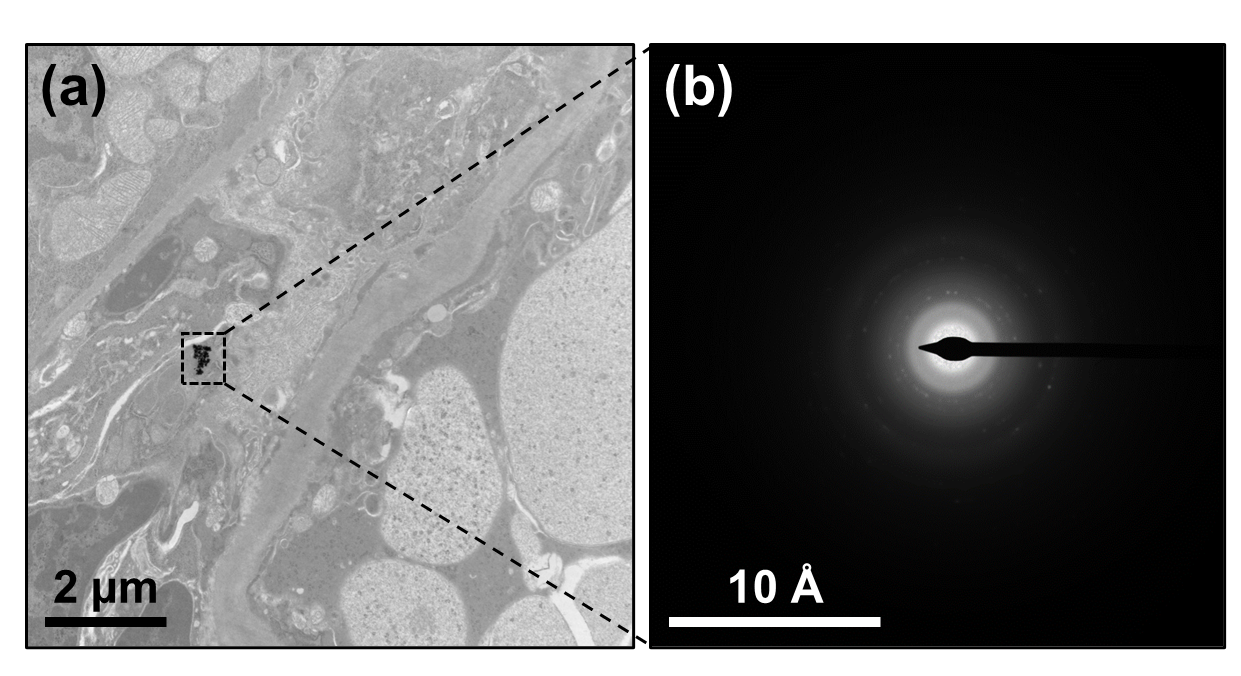


**Figure S14.** (a) TEM image of kidney showing F-AuNSs and (b) corresponding diffraction pattern of gold nanostars observed from the location indicated, which confirmed that the broken particles observed in the TEM image were indeed Au and not an artifact.

**
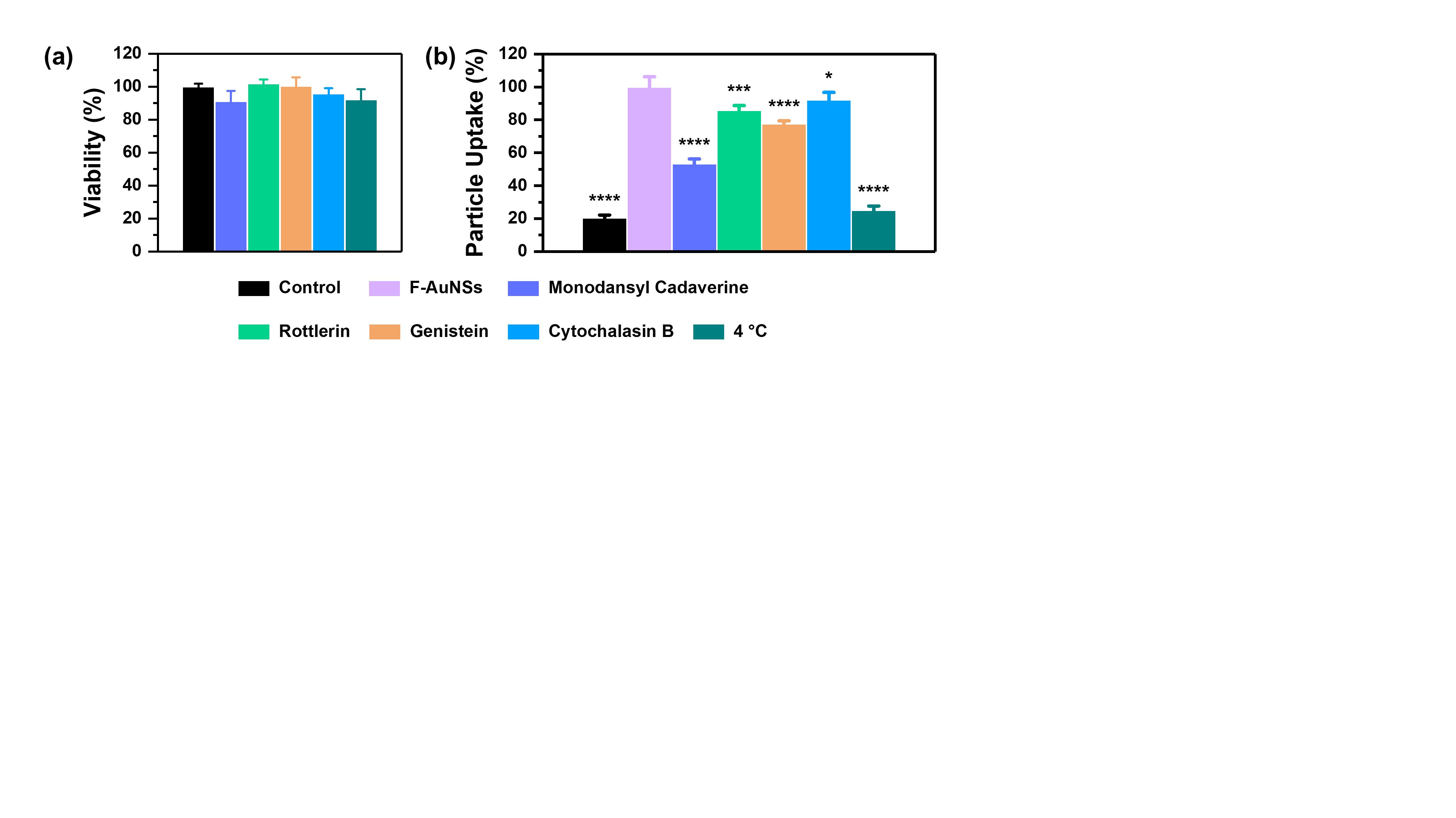
**

**Figure S15.** (a) MTT cell viability assay of RAW 264.7 incubated for 8 h with inhibitors of the different endocytosis pathways including monodansyl cadaverine (200 µM), rottlerin (2 µM), genistein (200 µM), cytochalasin B (10 µg/mL) and cells incubated at 4 °C. These cells did not receive any F-AuNSs. Black bar represents control cells that did not receive any inhibitors. Cell viability was measured at 540 nm. All data were presented as mean ± standard deviation (n = 5 per concentration and N = 2 independent experiments). (b) Endocytosis of F-AuNSs in RAW 264.7 incubated with different inhibitors including monodansyl cadaverine (200 µM, 10 min) for clathrin-mediated endocytosis, rottlerin (2 µM, 30 min) for macropinocytosis, genistein (200 µM, 1 h) for caveolae-mediated endocytosis, cytochalasin B (10 µg/mL, 2 h) for phagocytosis and 4 °C (1 h) for all energy-dependent uptake pathways, respectively. Cells without F-AuNSs and without inhibitor were denoted as ‘Control’ (negative control), and incubated with only F-AuNSs and without any inhibitors at 37 °C are marked as ‘F-AuNSs’ (positive control). Here, all data were represented as mean ± standard deviation (n = 4 per group and N = 2 independent experiments). * indicates *p* < 0.05, *** indicates *p* < 0.001, **** indicates *p* < 0.0001 *vs.* ‘F-AuNSs’ (positive control).


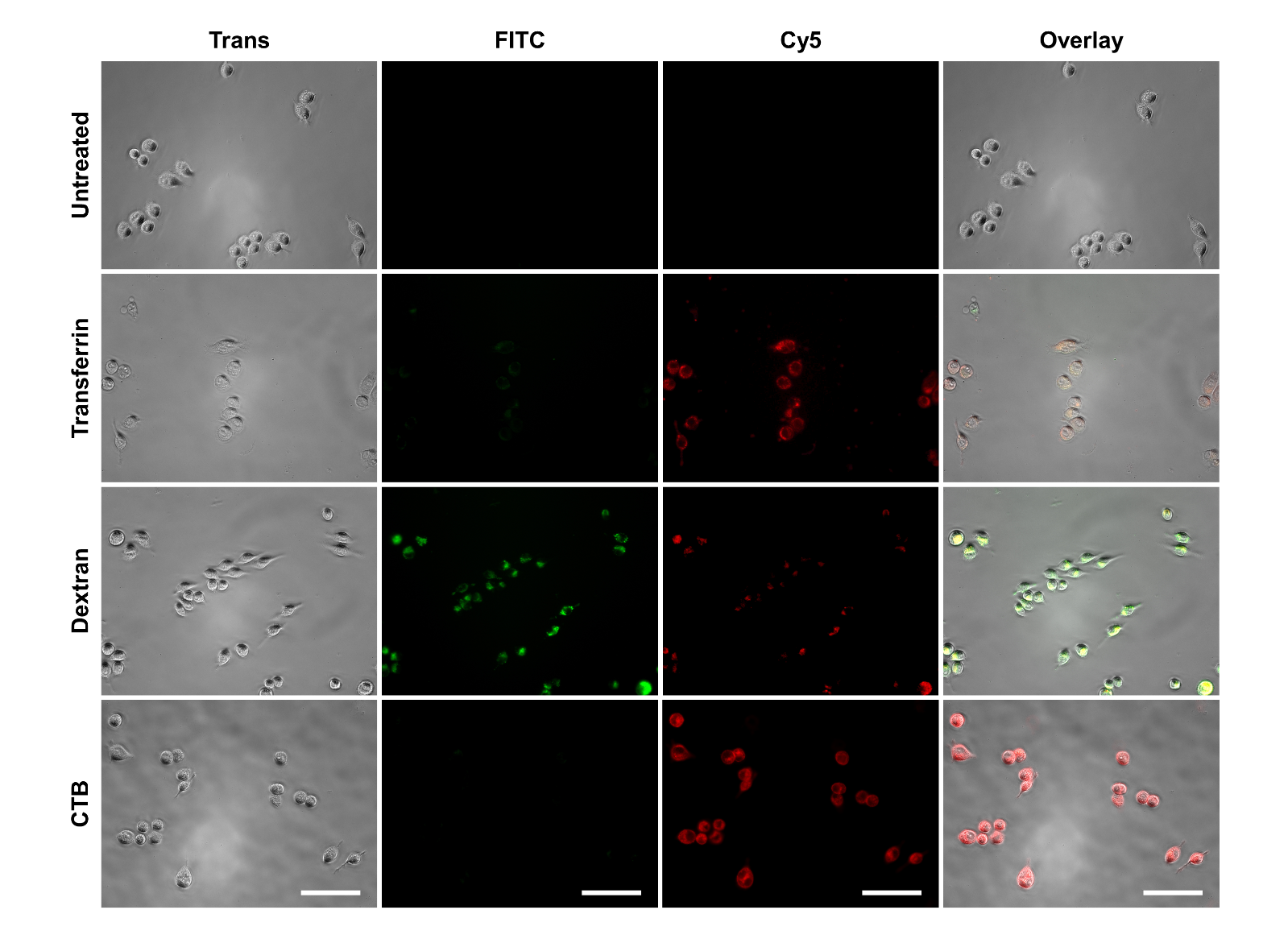


**Figure S16.** Fluorescence microscopy images of colocalization of F-AuNSs in J774A.1 incubated with different endocytic markers for 4 h including transferrin (0.05 mg/mL) for clathrin-mediated endocytosis, dextran (0.5 mg/mL) for macropinocytosis, and CTB (0.002 mg/mL) for caveolae-mediated endocytosis. Cells without F-AuNSs were denoted as ‘Untreated’. Fluorescently labeled F-AuNSs are shown as green, and endocytic markers are shown as red signal. Scale bar is 90 µm.


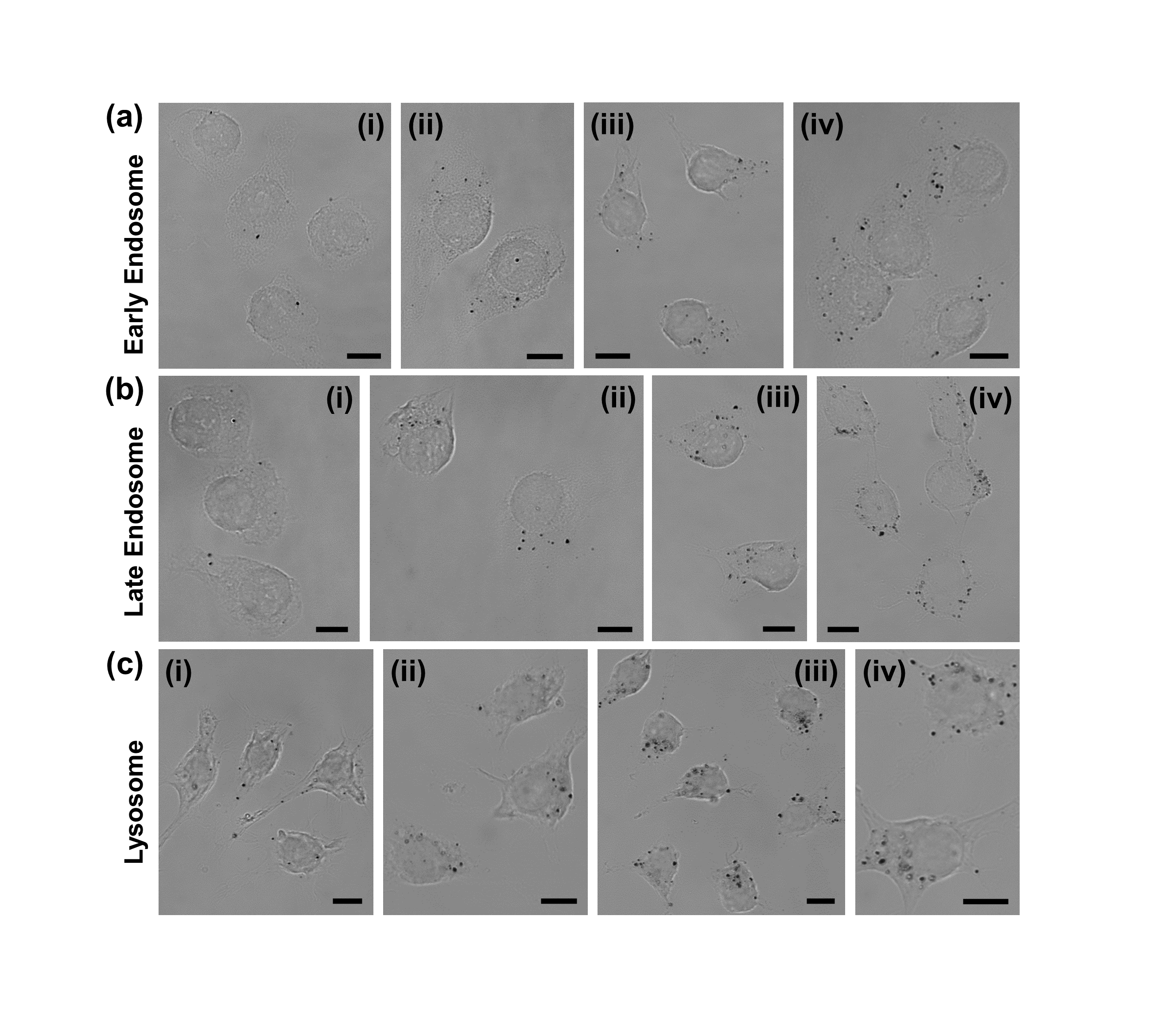


**Figure S17.** Brightfield images of F-AuNSs incubated with (a) early endosome for (i) 2, (ii) 4, (iii) 6 and (iv) 8 h, (b) late endosome for (i) 2, (ii) 4, (iii) 6 and (iv) 8 h, and (c) lysosome for (i) 4, (ii) 8, (iii) 18 and (iv) 24 h in J774A.1. Scale bar is 10 µm.


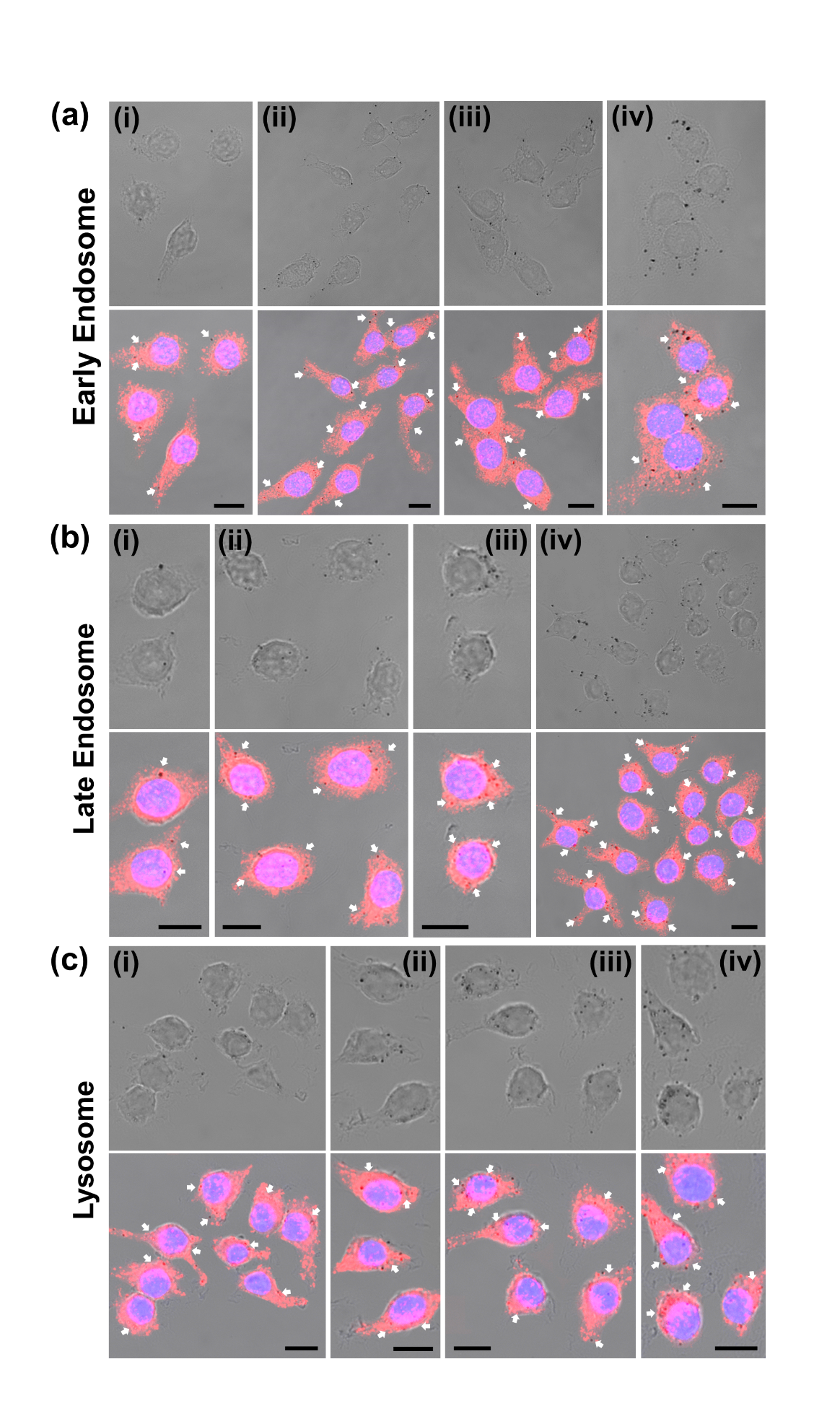


**Figure S18.** Colocalization of F-AuNSs with (a) early endosome shown as red fluorescence for (i) 2, (ii) 4, (iii) 6 and (iv) 8 h, (b) late endosome shown as red fluorescence for (i) 2, (ii) 4, (iii) 6 and (iv) 8 h, and (c) lysosome shown as pseudo-colored red for (i) 4, (ii) 8, (iii) 18 and (iv) 24 h in RAW 264.7. The nucleus in each cell was stained with DAPI (blue). Scale bar is 10 µm. White arrows point to particles.


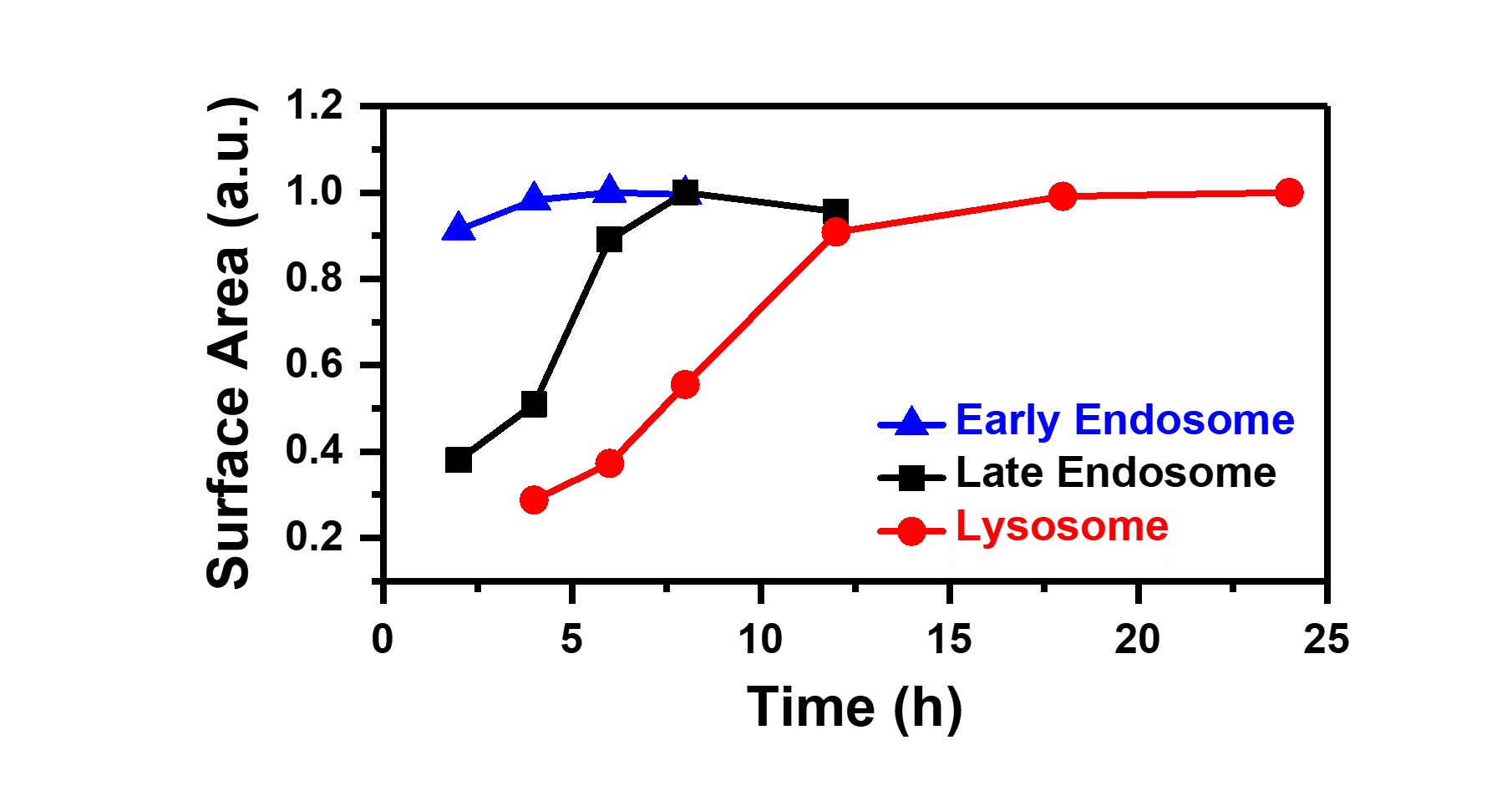


**Figure S19.** Total surface area of particles (black dots) normalized and colocalized with early endosome, late endosome, and lysosome in 50 RAW 264.7 cells.


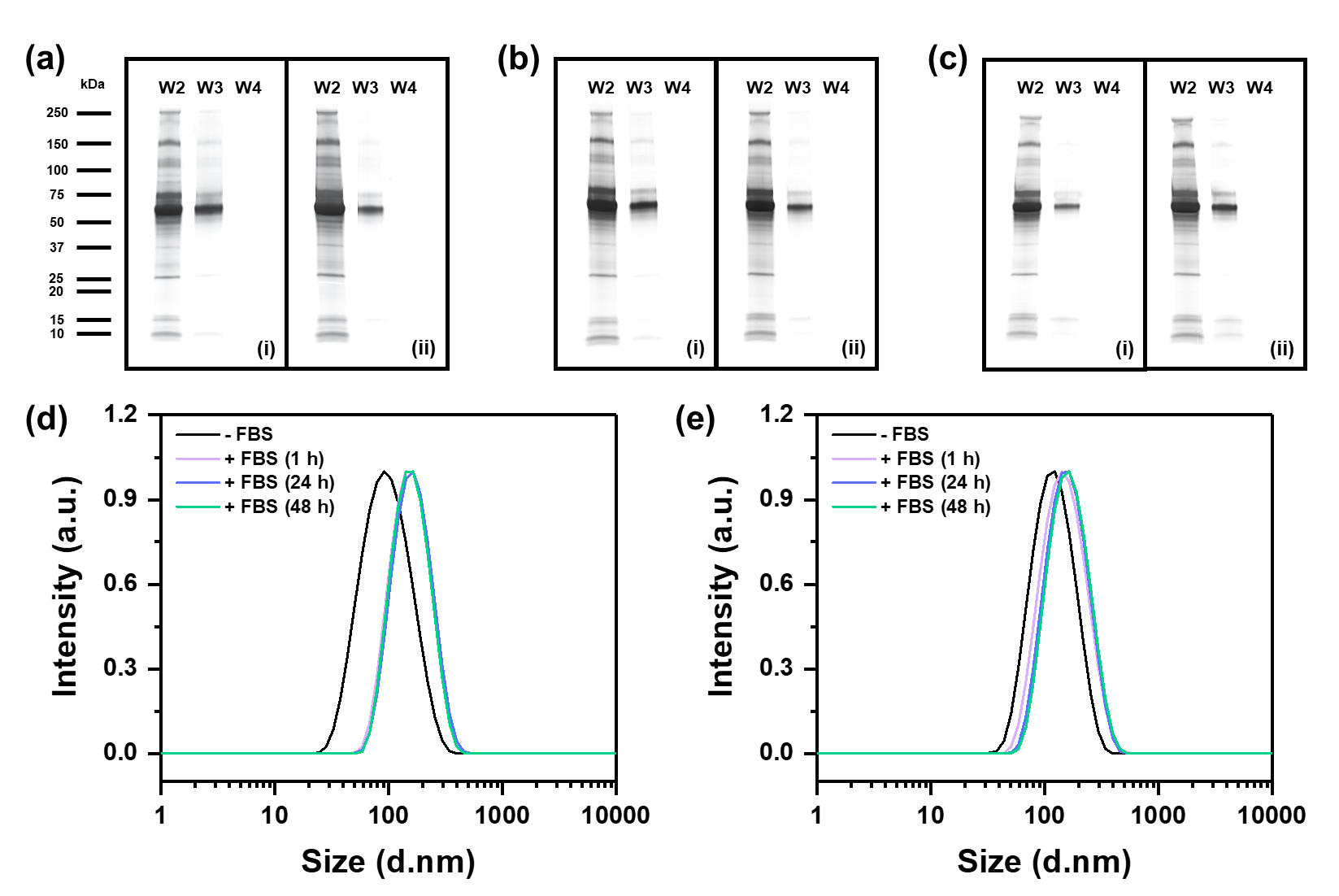


**Figure S20.** Proteomic study of surface protein corona formation on F-AuNSs. Silver-stained SDS-PAGE showed that large unbound and loosely bound proteins were effectively removed by repeated centrifugation and washing and no free proteins were detected in the last washing step for (i) B-AuNSs and (ii) F-AuNSs incubated with 60% FBS for (a) 1, (b) 24 and (c) 48 h. Here, *W2*, *W3* and *W4* represent washing step 2, 3 and 4. Hydrodynamic size of (d) B-AuNSs and (e) F-AuNSs after incubation with 60% FBS for 1, 24 and 48 h.


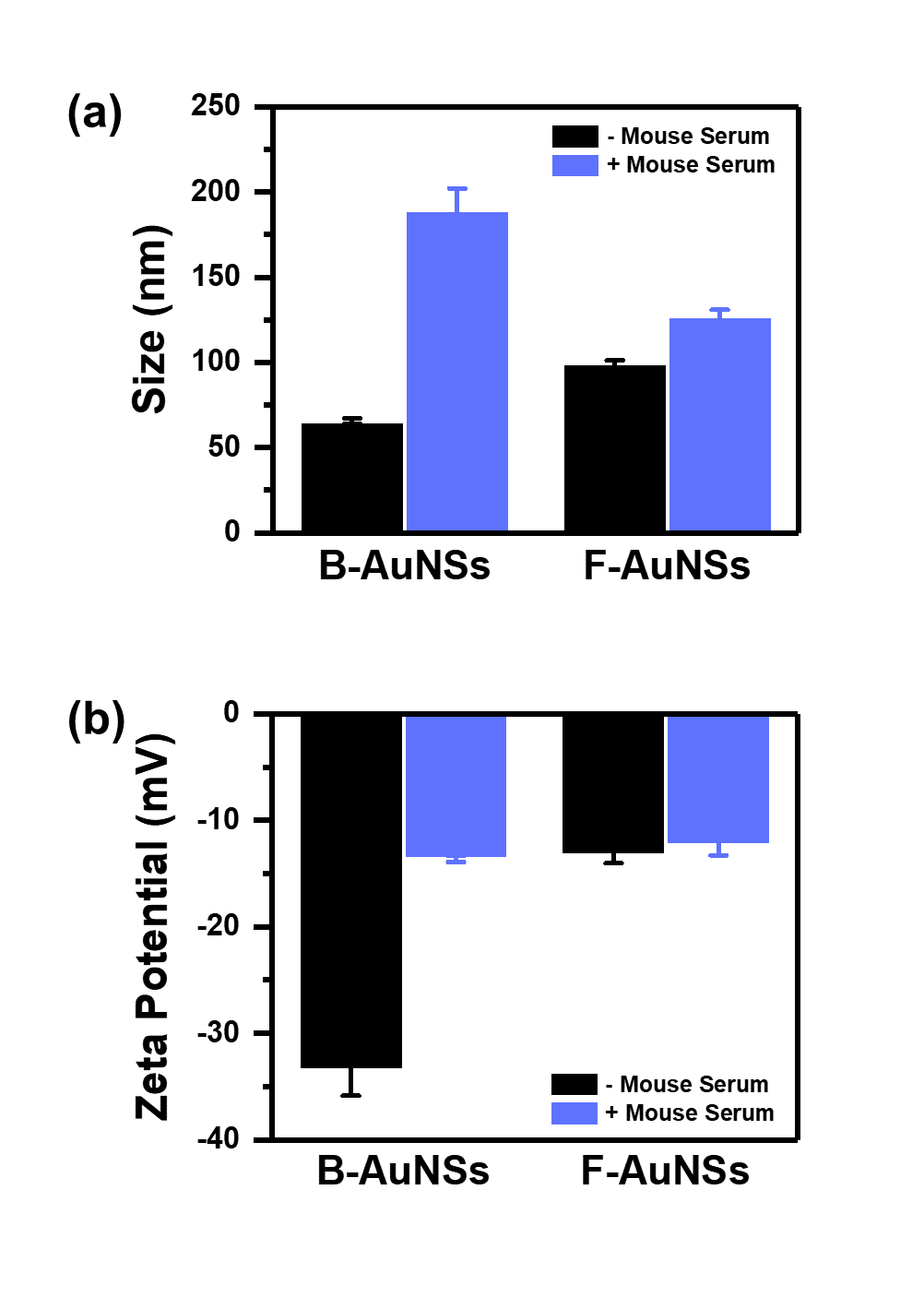


**Figure S21.** Proteomic study of surface protein corona formation. (a) Size and (b) surface charge analysis of B-AuNSs and F-AuNSs after incubation for 24 h with 60% serum derived from C57BL/6 mice. All data were represented as mean ± standard deviation (n = 3).

**Table S1.** Proteins adsorbed on B-AuNSs and F-AuNSs.

| **Category** | **B-AuNSs (%)** | **F-AuNSs (%)** |
| --- | --- | --- |
| Acute Phase | 37.4 | 47.7 |
| Coagulation | 27.1 | 18.9 |
| Cytoskeletal/ECM | 9.5 | 20.9 |
| Complement | 9.8 | 9.2 |
| Apolipoprotein | 2.1 | 2.1 |
| Others | 14.1 | 1.2 |

| **Molecular Weight** | **B-AuNSs (%)** | **F-AuNSs (%)** |
| --- | --- | --- |
| <20 | 18.1 | 5.0 |
| 20 – 40 | 16.5 | 17.6 |
| 40 – 60 | 16.0 | 19.4 |
| 60 – 80 | 25.7 | 36.3 |
| 80 – 100 | 5.8 | 1.2 |
| 100 – 200 | 14.6 | 15.8 |
| >200 | 3.3 | 4.7 |

| **pI** | **B-AuNSs (%)** | **F-AuNSs (%)** |
| --- | --- | --- |
| <5 | 2.7 | 4.7 |
| 5 – 6 | 19.1 | 23.0 |
| 6 – 7 | 35.7 | 45.6 |
| 7 – 8 | 26.9 | 10.1 |
| 8 – 9 | 10.2 | 3.3 |
| 9 – 10 | 4.9 | 13.1 |
| >10 | 0.5 | 0.2 |


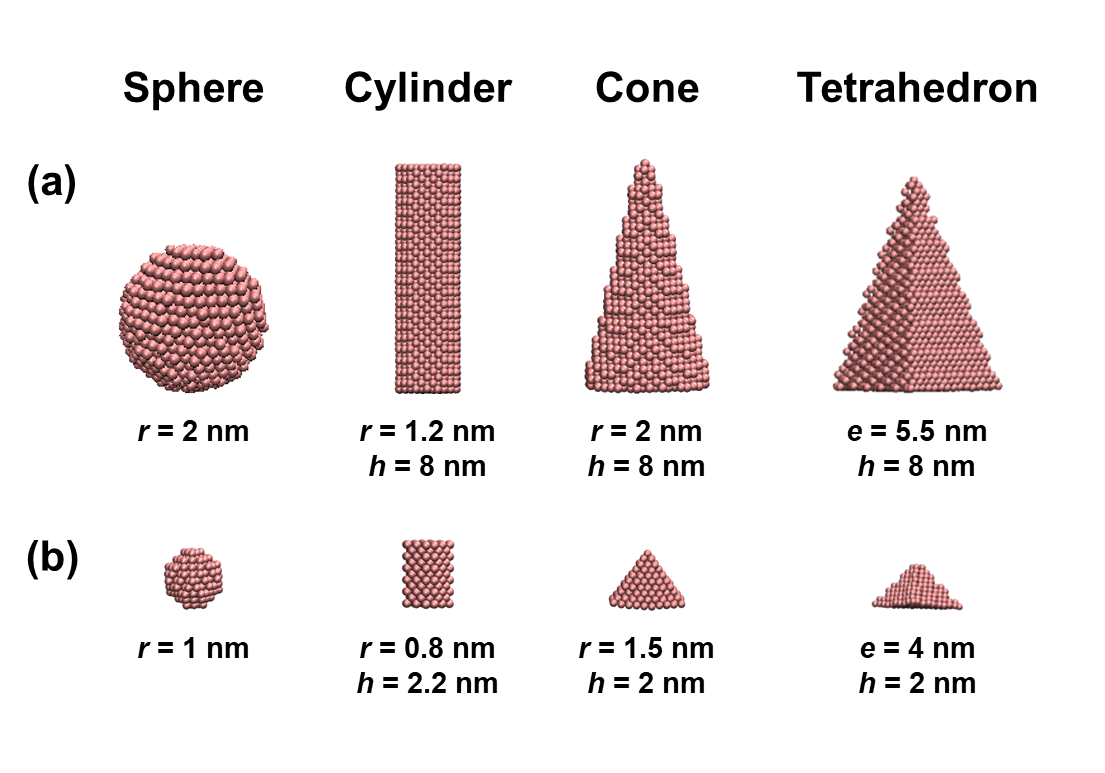


**Figure S22.** Snapshots of four different shapes of nanoparticles. The (a) top and (b) bottom rows contain side-view of the nanoparticles with a volume ~35 and 4.5 nm^3^, respectively. Here, *r*, *h* and *e* represent radius, height and edge, respectively.


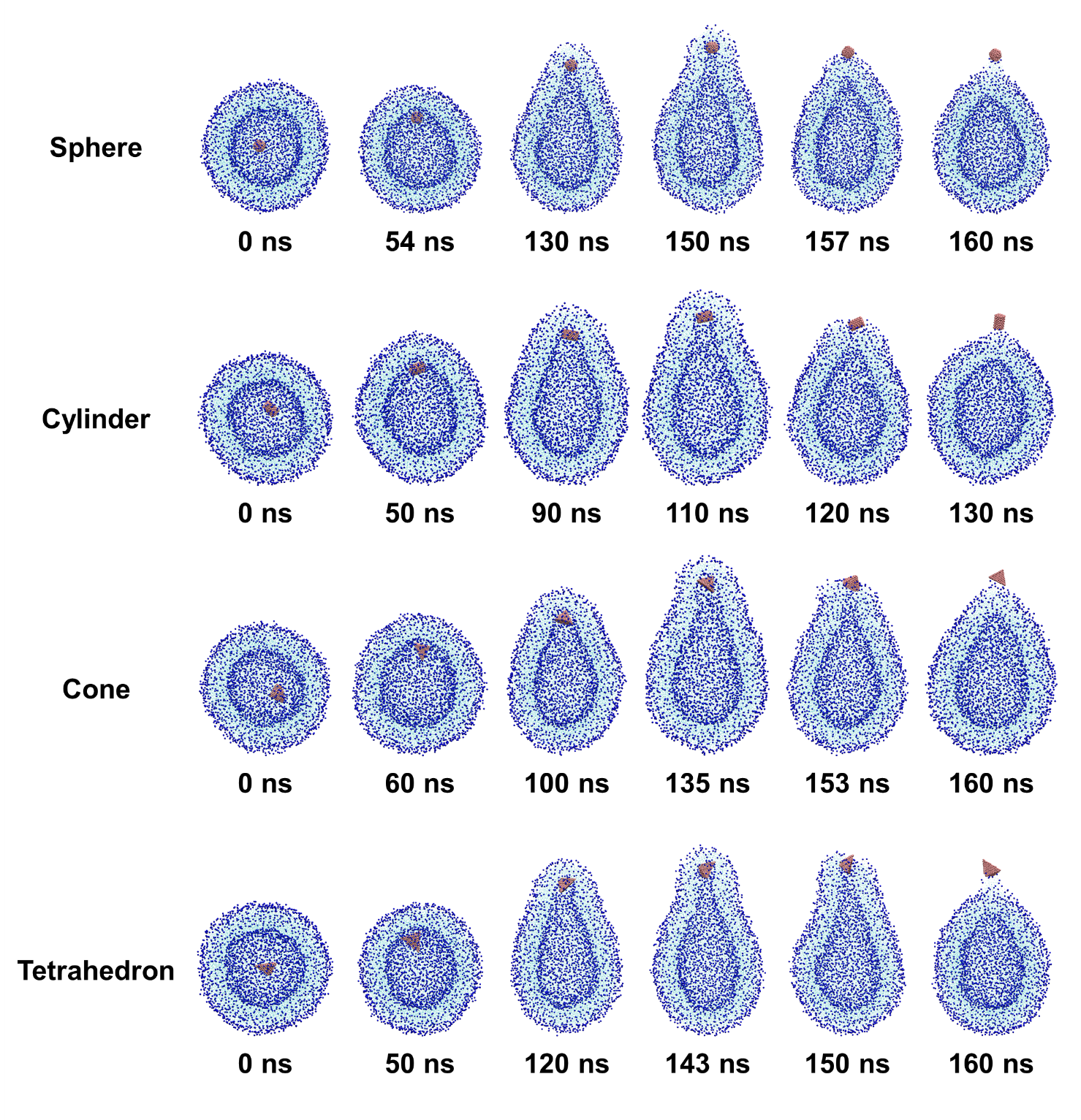


**Figure S23.** Snapshots of the translocation of small-sized NPs across the DPPC lipid membrane. Four different shapes of NPs with an equivalent volume of 4.5 nm^3^ were evaluated.


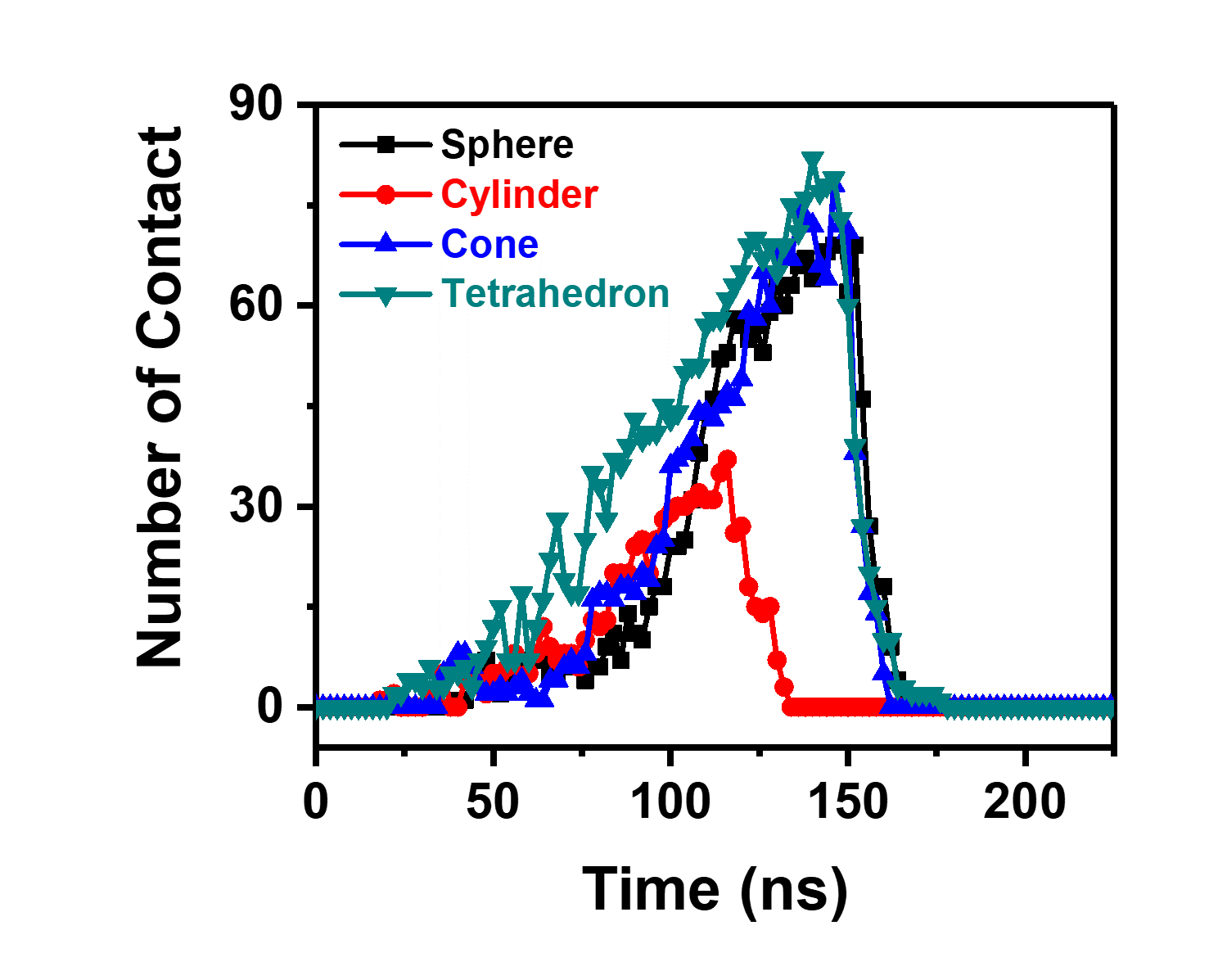


**Figure S24.** The number of contacts between the small-sized NPs with four different shapes and membrane composed of DPPC lipid as a function of time showing that cylinder shaped NPs had the least interaction with membrane and faster transcytosis.


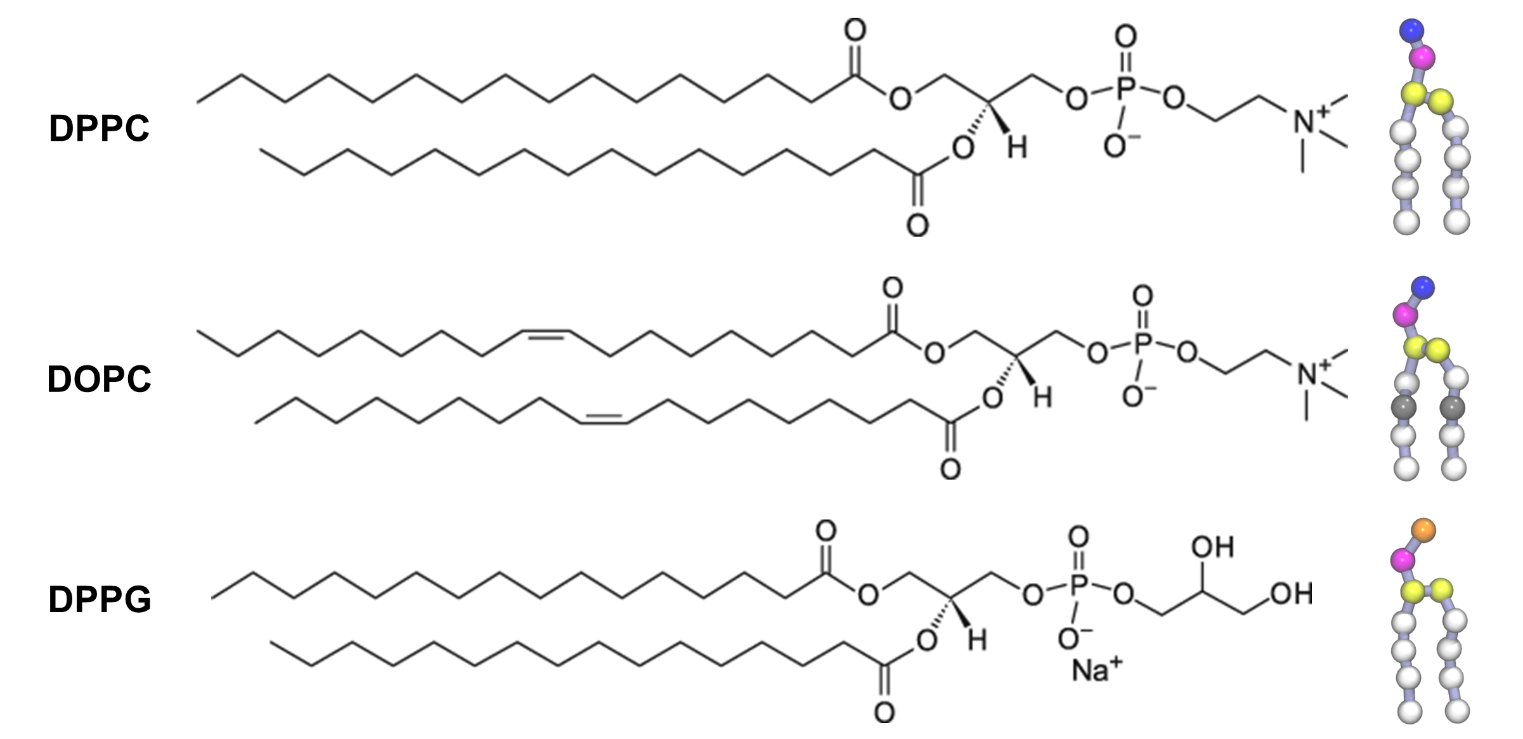


**Figure S25.** Chemical structures of DPPC, DOPC and DPPG lipids depicting the different membranes in our simulations.


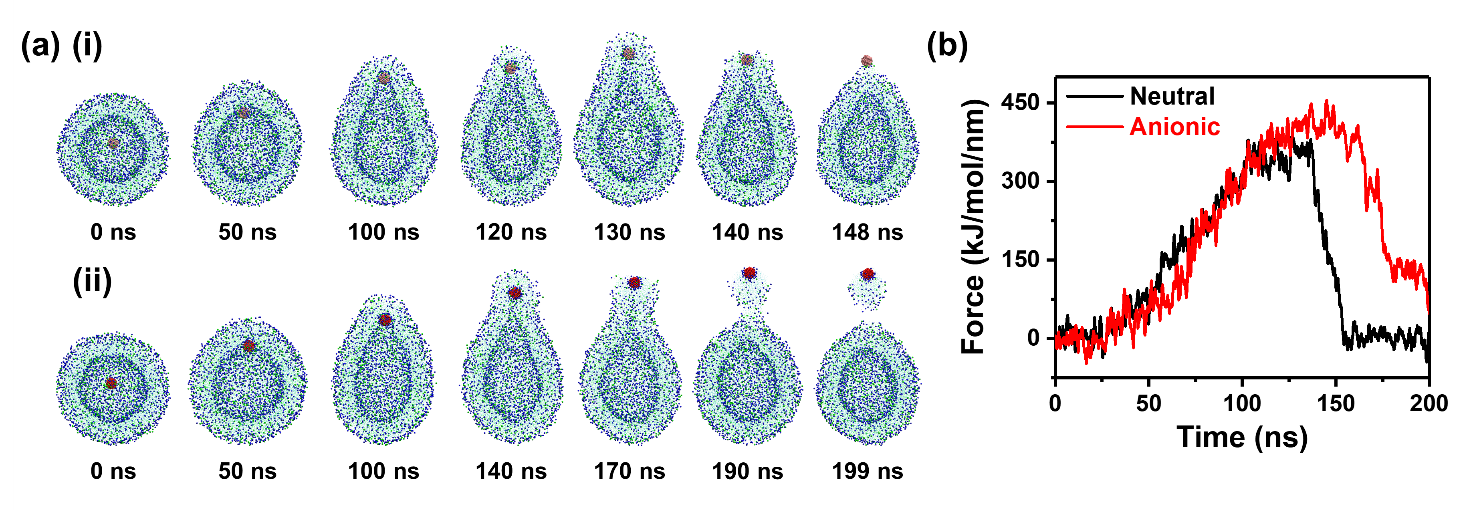


**Figure S26.** Effect of lipid composition on transcytosis. (a) Snapshots of translocation of (i) neutral and (ii) anionic spherical NPs (volume ≈ 4.5 nm^3^) across the model cell membrane composed of DPPC mixed with DPPG lipids at a ratio of 3:1 for cellular transcytosis. (b) The interaction force between the neutral or anionic spherical NPs and mixed DPPC-DPPG lipid membranes as a function of time.


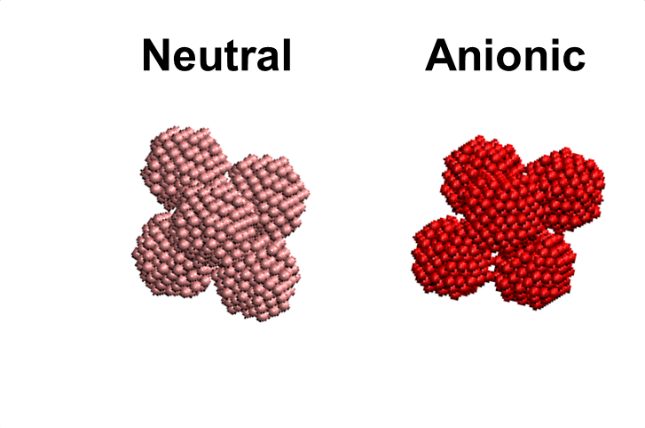


**Figure S27.** Snapshots of aggregated AuNPs with neutral and anionic surface charges and with an overall diameter of ~4 nm.


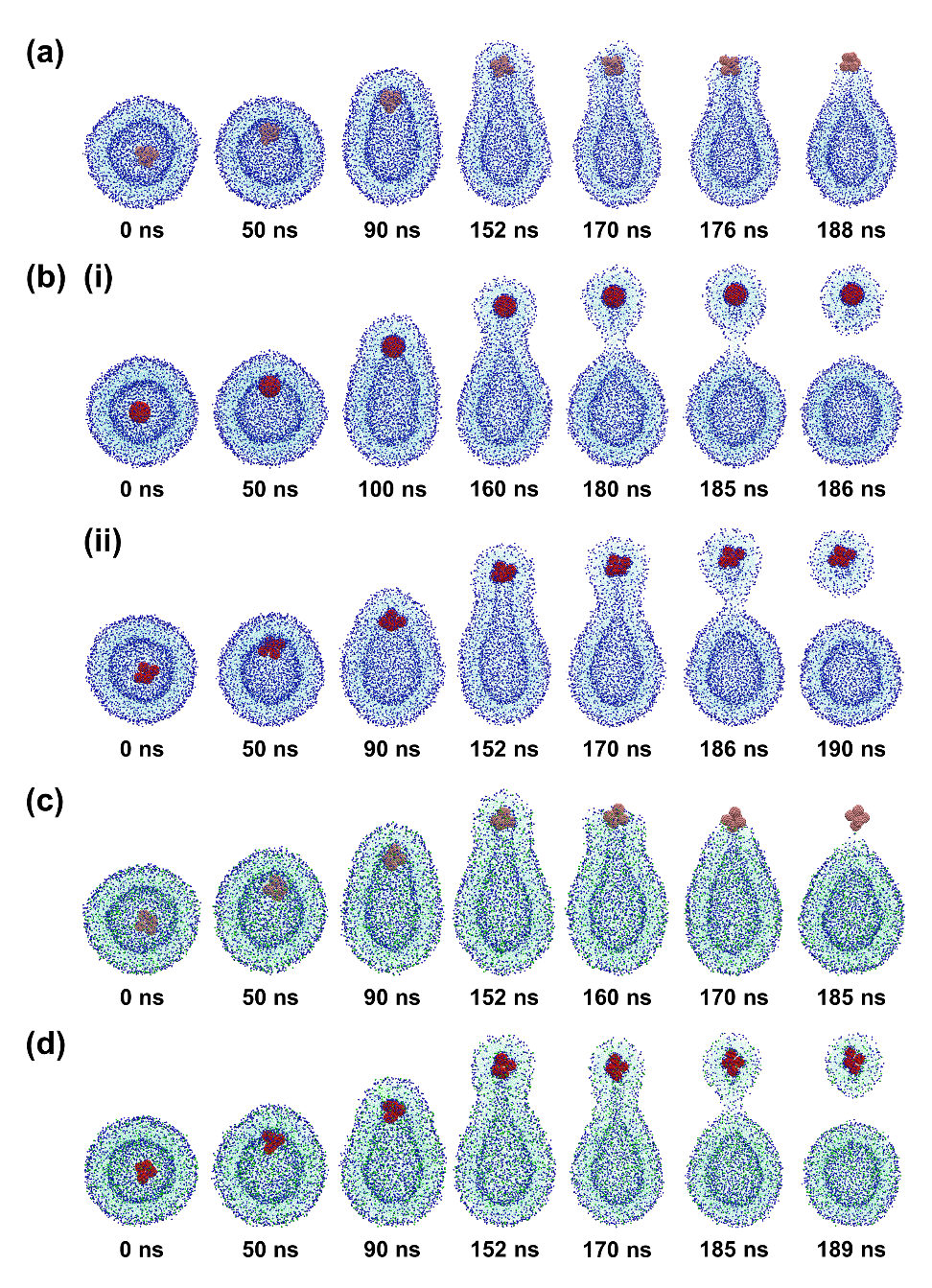


**Figure S28.** Effect of aggregation on transcytosis. (a) Snapshots of translocation of neutral aggregated NPs across the DPPC lipid membrane. (b) Snapshots of translocation of anionic (i) monodisperse single NP and (ii) aggregated NPs across the DPPC lipid membrane. The aggregated NP has similar size and volume as the monodisperse single spherical NP (4 nm diameter, and ~35 nm^3^ volume). Snapshots of translocation of (c) neutral and (d) anionic aggregated NPs across the model cell membrane composed of DPPC mixed with DPPG lipids at a ratio of 3:1 for cellular transcytosis.


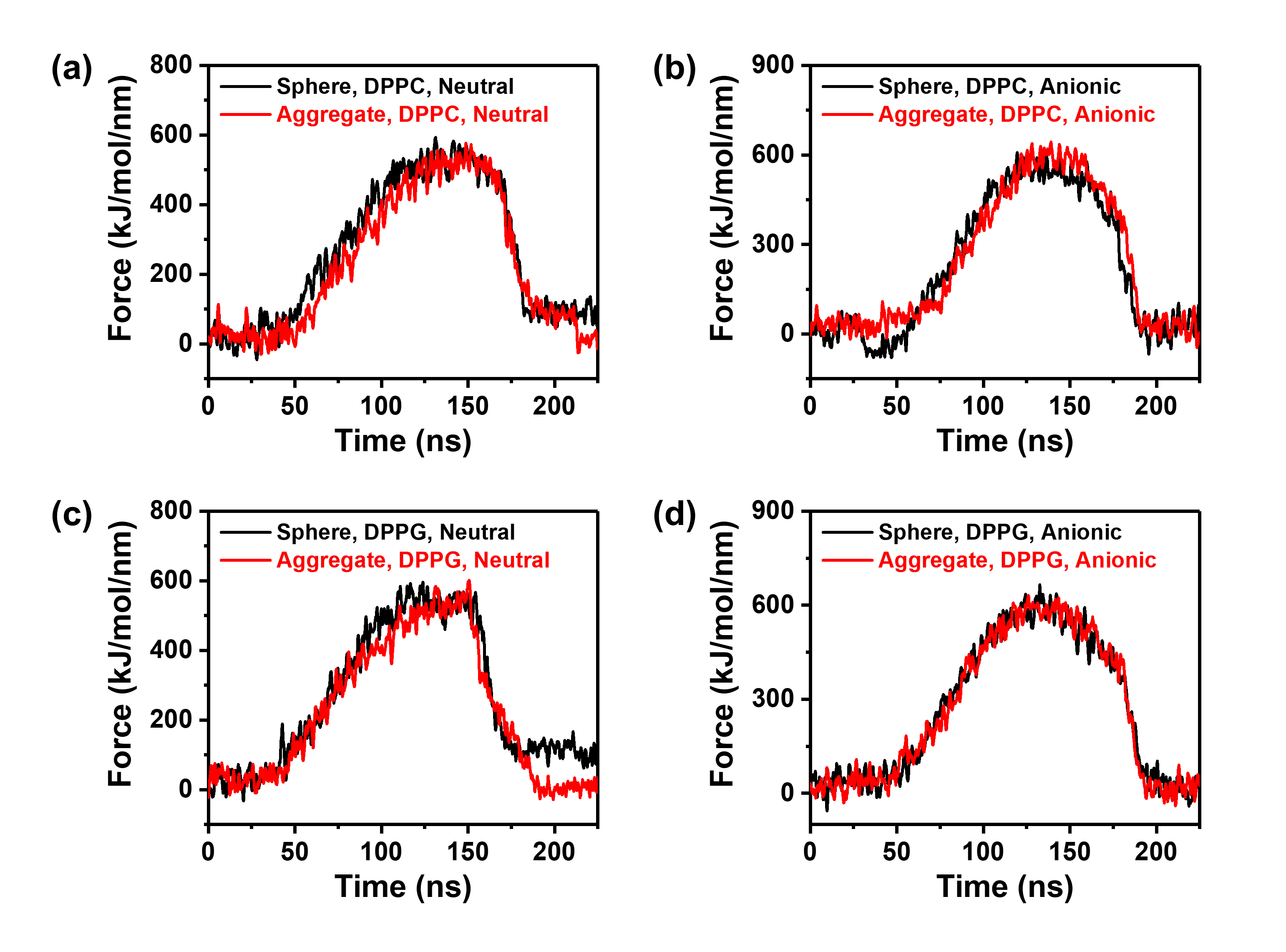


**Figure S29.** Effect of aggregation on transcytosis. (a) The interaction force between the neutral aggregated or single spherical NPs and DPPC lipid membranes as a function of time. (b) The interaction force between the anionic aggregated or single spherical NPs and DPPC lipid membranes as a function of time. (c) The interaction force between the neutral aggregated or single spherical NPs and mixed DPPC-DPPG lipid membranes as a function of time. (d) The interaction force between the anionic aggregated or single spherical NPs and mixed DPPC-DPPG lipid membranes as a function of time.
